# Prediction of Chemical-Protein Interactions Network with Weighted Network-Based Inference Method

**DOI:** 10.1371/journal.pone.0041064

**Published:** 2012-07-16

**Authors:** Feixiong Cheng, Yadi Zhou, Weihua Li, Guixia Liu, Yun Tang

**Affiliations:** Shanghai Key Laboratory of New Drug Design, School of Pharmacy, East China University of Science and Technology, Shanghai, China; Russian Academy of Sciences, Institute for Biological Instrumentation, Russian Federation

## Abstract

Chemical-protein interaction (CPI) is the central topic of target identification and drug discovery. However, large scale determination of CPI is a big challenge for *in vitro* or *in vivo* experiments, while *in silico* prediction shows great advantages due to low cost and high accuracy. On the basis of our previous drug-target interaction prediction *via* network-based inference (NBI) method, we further developed node- and edge-weighted NBI methods for CPI prediction here. Two comprehensive CPI bipartite networks extracted from ChEMBL database were used to evaluate the methods, one containing 17,111 CPI pairs between 4,741 compounds and 97 G protein-coupled receptors, the other including 13,648 CPI pairs between 2,827 compounds and 206 kinases. The range of the area under receiver operating characteristic curves was 0.73 to 0.83 for the external validation sets, which confirmed the reliability of the prediction. The weak-interaction hypothesis in CPI network was identified by the edge-weighted NBI method. Moreover, to validate the methods, several candidate targets were predicted for five approved drugs, namely imatinib, dasatinib, sertindole, olanzapine and ziprasidone. The molecular hypotheses and experimental evidence for these predictions were further provided. These results confirmed that our methods have potential values in understanding molecular basis of drug polypharmacology and would be helpful for drug repositioning.

## Introduction

Over the past decade, the productivity of drug research and development (R&D) seems to be decreasing [1]. Richard *et al*. stated that at present more than 800 drugs are in clinical development for cancer indications and the current success rate in bringing drugs to the markets remains only in the range of 5–8% [2]. One reason about R&D decrease might be due to the domination of “one-disease-one-drug-one-target' paradigm [1]. Several clinical investigations confirmed that most drugs act on multiple targets rather than one target, that is, drug polypharmacology [1], [3]. For example, tamoxifen, which is an approved drug used to treat breast cancer for more than 30 years, has been found to be effective in experimental models of cutaneous and visceral leishmaniasis [4]. Several well-known drugs such as thalidomide, sildenafil, bupropion and fluoxetine were found new uses beyond their original approved therapeutic indications [5].

Study of chemical-protein interactions (CPI) network is an important topic toward elucidation of protein functions, understanding of molecular mechanisms inside the cell and drug repositioning. It is both time-consuming and costly to identify CPI by experiments alone. As a complement, *in silico* method could provide us with very useful information in a predictable, reliable, less costly and timely manner. Various *in silico* methods have been proposed to address the CPI prediction. The classical methods can be classified into ligand-based and target-based ones. For example, Humberto *et* *al*. developed a multi-target QSAR classifier and built a web server for CPI prediction [6]. Another widely used target-based method is reverse molecular docking. Several web servers, such as TarFisDock [7], DRAR-CPI [8] for drug discovery and CPI prediction have been developed. However, this method cannot be applied to targets whose three-dimensional (3D) structures are unresolved, especially for membrane proteins like G protein-coupled receptors (GPCRs), and were limited usage due to time-costly and the inaccuracy of the scoring functions.

Recently, several new methods, such as computational chemogenomics, phenotype-based and network-based diffusion methods were successful proposed for CPI prediction [9]–[23]. Yamanishi *et al*. developed a bipartite graph learning method for drug-target interaction (DTI) prediction [9]. Recently, Yamanishi *et al*. further developed DTI prediction method by integrating chemical, genomic and pharmacological spaces [21]. Though high overall predictive accuracy was yielded in the Yamanishi's work, the sensitivity was anomaly low and the method was not validated experimentally. Wang *et al*. developed a computational chemogenomics method from protein primary sequences and used it to identify several new ligands for four targets (i.e., GPR40, SIRT1, p38, and GSK-3β) validated by experimental assays [11]. The drawback of chemogenomics method is that there are a huge number of samples to be classified, which increase the computational complexity. Another bottleneck is the lack of the benchmark negative CPI pairs and it easily results in high false positive rate. Our recent work found that there is high false positive rate in computational chemogenmics method, and the performance of chemogenmics method was influenced by data set bias and features selection methods [23].

In our previous paper, we reported three supervised inference methods: drug-based similarity inference (DBSI), target-based similarity inference (TBSI) and network-based inference (NBI) methods for DTI prediction and drug repositioning derived from complex network theory [24], [25]. With the methods, five known drugs were predicted and experimentally validated to have novel indications on estrogen receptors and dipeptidyl peptidase-IV [26]. However, the methods are only suitable for drugs having known links to targets in the training set and the unweighted DTI network among drug and target nodes was used. Whether the weighted DTI network could improve the predictive accuracy or the method could be extended to general CPI prediction has not been investigated yet.

In this paper, the above-mentioned three methods were further improved. Two new methods, namely node-weighted network-based inference (NWNBI) and edge-weighted network-based inference (EWNBI) were further presented, and four similarity metrics (Tanimoto, Cosine, Forbes and Russell-rao) were explored in the DBSI method systematically, for CPI prediction and drug repositioning. The methods were then examined with two comprehensive CPI databases targeting GPCRs and kinases, respectively. The new targets were further predicted for five example known drugs, and experimental evidences to support the predictions were provided.

## Materials and Methods

### Data Preparation

Two comprehensive CPI data sets were collected from the ChEMBL database (https://www.ebi.ac.uk/chembl/, accessed in May. 2010) [27]. The initial database includes 1,195,368 compounds and more than 8,000 targets from various species. Here, we only focused on two pharmacologically important families, GPCRs and kinases. The data sets were refined with the following criteria: (1) only human test data were selected; (2) only those with K_i_ or IC_50_ values less than 10 μM were extracted; (3) proteins connected with less than three active compounds were excluded; (4) proteins with non-standard amino acids, DNA, RNA, or sequence length less than 100 residues were removed; and (5) nonorganic chemicals and chemicals with molecular weight less than 100 Dalton or more than 600 Dalton were also excluded. All compounds in SMILES format and proteins sequence in FASTA format were extracted from ChEMBL.

### Network Construction

The methods adopted in this paper are to prioritize unconnected candidate proteins for a given chemical, or prioritize unconnected candidate chemicals for a given protein, which derived from the recommendation algorithms of complex network theory [26]. We constructed two comprehensive CPI bipartite networks (or graphs) to represent the data in chemical nodes, protein nodes and their physical interactions. Denoting the chemical set as 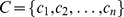
and the protein set as 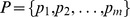, the CPI binary pairs can be described as a bipartite CPI graph 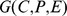, where 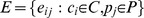. A link is drawn between *c_i_* and *p_j_* only if the K_i_ or IC_50_ was less than 10 μM between *c_i_* and *p_j_*. The CPI bipartite graph can be presented by an 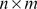 adjacent matrix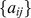, where 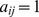 when K_i_ or IC_50_ value less than 10 μM, otherwise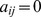.

### Methods Development

**Figure 1:**
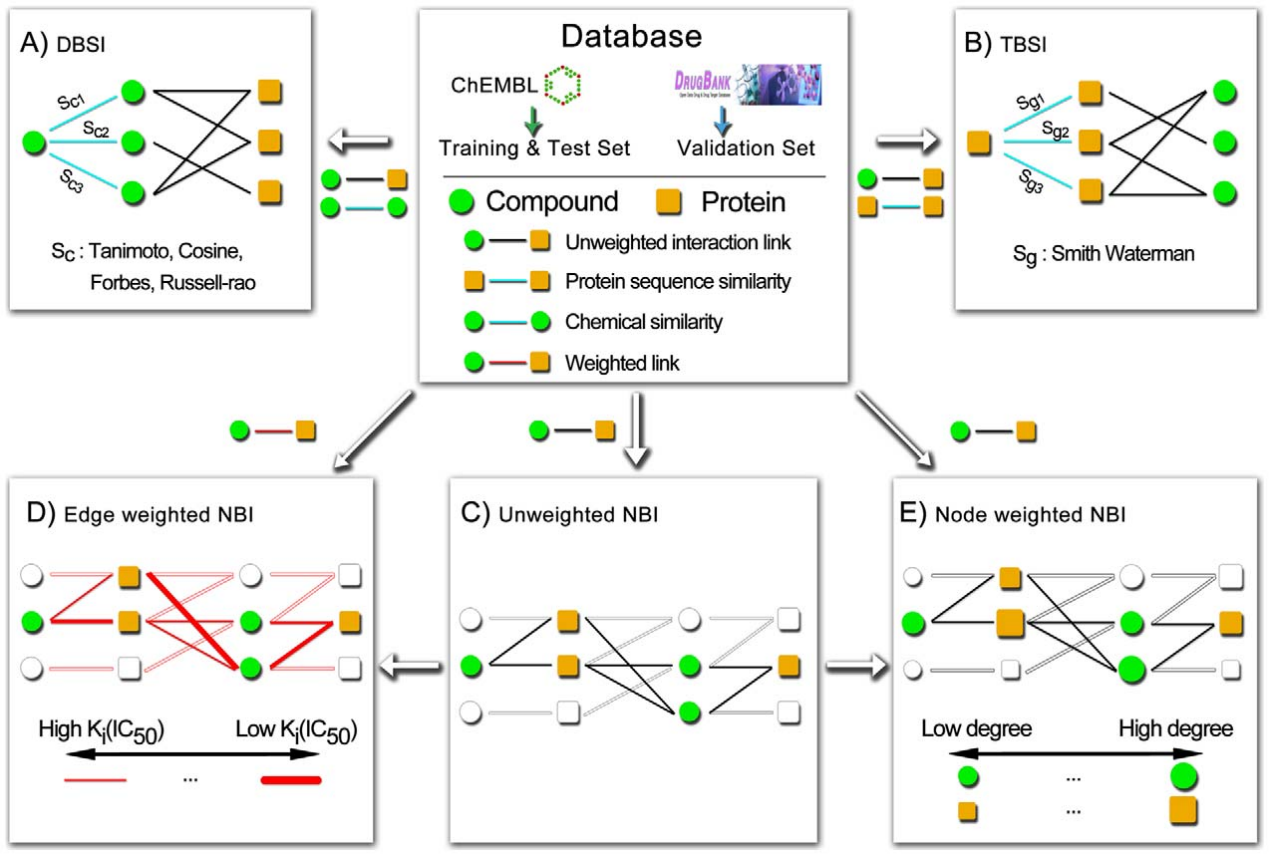
Schematic diagram of our proposed method. (**A**) The drug-based similarity inference (DBSI), (**B**) the target-based similarity inference (TBSI) and (**C**) the unweighted network-based inference (NBI), (**D**) the edge-weighted NBI (EWNBI) and (**E**) the node-weighted NBI (NWNBI). Green circle: chemical node, gold square: protein node, black line: unweighted interaction link, cyan line: chemical-chemical two-dimensional structural similarity (S_c_) or protein-protein Smith Waterman genomic similarity (S_g_), red line: weighted edges (thick red line denotes the strong edge with high potency and thin red line denotes the weak edge with low potency).

In our previous work, we proposed three inference methods, i.e. DBSI, TBSI and NBI, to predict DTI. In this study, we managed to improve the NBI method with weighted nodes or edges. The entire workflow was illustrated in Figure 1.

#### Drug-Based Similarity Inference (DBSI)

The DBSI method was designed based on the hypothesis that two chemicals with similar chemical structures may exhibit similar bioactivities (Figure 1A), which was described in our previous work [26]. For a CPI pair 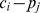, if *c_i_* has not interacted with *p_j_* yet, the predicted score by this method is given as:
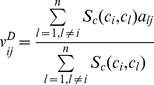
(1)



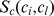
 indicates two-dimensional (2D) chemical structural similarity between chemicals 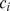 and 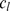. In this study, four different chemical structure similarity metrics, namely Tanimoto, Cosine, Forbes and Russell-rao were systemically evaluated using MACCS keys, freely available from OpenBabel (version 2.3.0) [28]. The further descriptions about four similarity metrics were given in the work of Willett *et al*. [29].

#### Target-Based Similarity Inference (TBSI)

The TBSI method was designed based on the hypothesis that two proteins with similar genomic space may exhibit similar biology function (Figure 1B). For any CPI pair 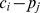, if *c_i_* does not connect with *p_j_* in the bipartite graph, the predicted score by this method is given as:
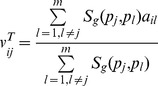
(2)



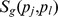
 indicates the genomic sequence similarity between two proteins 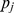 and 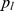. The sequence similarity between protein 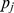 and 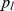 was computed by the Smith-Waterman scores [30].

#### Unweighted Network-based Inference (NBI)

Considering the bipartite graph 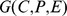, we applied a mass diffusion-based method to obtain the predicted list. For a given chemical *c_i_*, supposing that a kind of resource is initially located in the proteins which are interacted with *c_i_*, the resource will diffuse to all the proteins in the network after the network-based resource allocation process [24], [25]. Each protein node averagely distributes its resource to all neighboring chemical nodes and then each chemical redistribute the received resource to all neighboring protein nodes. The finial resource on the proteins that are not connected with the chemical *c_i_* in 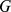 could be considered as the score of each protein, and the proteins with high score are more likely to interact with *c_i_*. Figure 1C gives a simple example to illustrate the network-based resource allocation process. It shows the initial resource of *a_ij_* between *c_i_* (green cycle) and 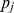 (orange square) followed as:
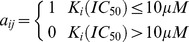
(3)


Denoting 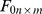 as the initial resource matrix (adjacency matrix) and 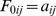, 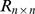 as the total resource (degree) of each chemical and 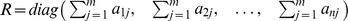, 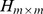 as the total resource (degree) of each protein and 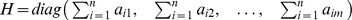, the final resource matrix will be obtained as 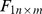, and 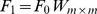 or 
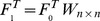
, where transfer matrix 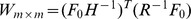 or 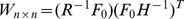.

#### Edge Weighted Network-based Inference (EWNBI)

In the above unweighted NBI method, we only consider the binary CPI pairs among nodes. However, the edges among chemicals and proteins are naturally weighted in the real biology world. For the EWNBI method, each edge of CPI network was weighted by the potency (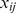 = −log_10_(*K_i_* (or *IC_50_*)/100 μM)) of binding affinity (*K_i_*) or inhibitory activity (*IC_50_*) of the physical interactions between the chemical node 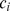 and protein node 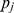.

Figure 1D gives a simple example to illustrate the edges weighted network-based resource allocation process. The initial resource of 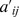 between 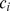 (green cycle) and 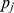 (orange square) were defined as follows:
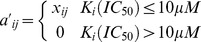
(4)


Denoting 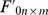 as the initial resource matrix and 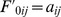, 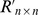 as the total resource of each chemical and 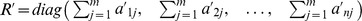, 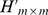 as the total resource of each protein and 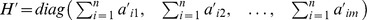, the final resource matrix will be obtained as 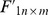, and 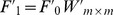 or 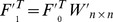, where transfer matrix 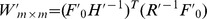 or 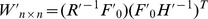.

#### Node Weighted Network-based Inference (NWNBI)

Compared to the earlier unweighted NBI method, we use a new expression of initial resource distribution of nodes and take into account the influence of resources associated with the receiver nodes in CPI bipartite network proposed by Jia *et al*. [31]. This method is based on the general knowledge that the hub node with more resources is more difficult to be influenced. Figure 1E illustrates the NWNBI method. For the initial resource matrix, the resources of each chemical and protein node are the same to the unweighted NBI method. The final resource matrix were calculated as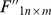, and 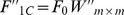 for chemicals and 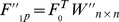 for proteins, where transfer matrix 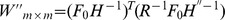, where 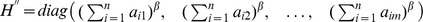 for chemical or 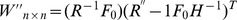, where 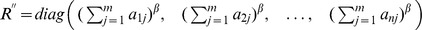 for protein, β is a tunable parameter which was used to control the influence. Compared with uniform case, *β* = 0, a positive *β* value strengthens the influence of hub nodes, while a negative *β* value weakens the influence of hub nodes. The detailed description can be found in Jia's work [31].

### Performance Assessment

All performance was assessed based on 10-fold cross validation techniques. In 10-fold cross validation, the entire compound-protein pairs were equally divided into ten cross splits. In each step of cross validation, the model was trained on a set of nine cross validation splits together. The tenth sub-sample set was used as an internal validation set (test set). In order to eliminate the error caused by dividing the data set, all the results were obtained by independent simulation 10 times test. With the randomly splitting, some proteins (or chemicals) maybe just in the test set and the corresponding links couldn't be predicted with our methods, because of no links for these proteins or chemicals in the training set. Such links were not considered in the performance assessment. Mathematically speaking, all methods provide each given chemical with an candidate queue of all its unconnected proteins (C_i_ (P_a_, P_b_, …P_m_)) or provide each given protein with an candidate queue of all its unconnected chemicals (P_j_ (C_a_, C_b_, …C_n_)). For each predicted list, we consider the topside links as the most possible candidate CPI. The CPI pairs that were predicted correctly are termed true positive, and the predicted interactions that are not in the test set are referred to as false positive. The area under the receiver operating characteristic curve (AUC) and recall (*R*) were calculated to assess the performance [26]. In addition, we also calculated the recall enhancement (ER) metric [24].

## Results

### Network Topology Analysis

Based on the criteria described above, 17,111 CPI among 4,741 unique compounds and 97 GPCRs with K_i_ less than 10 μM, and 13,648 CPI among 2,827 unique compounds and 206 kinases with IC_50_ less than 10 μM were collected (Table 1). The K_i_ values, IC_50_ values, SMILES, FASTA, compound ID of all compounds and targeted proteins were given in Tables S1 and S2. The CPI networks of GPCRs and kinases were constructed using a bipartite graph. Figure S1 gives the degree distributions of each chemical and protein. The degree of protein node is the number of chemicals that the protein links with. The degree of chemical node is the number of proteins that the chemical links with. The numbers of chemicals and proteins, the average degree and the sparsity values were given in Table 1. Sparsity is the proportion of the known CPI over all the possible interactions. There is a manifest ligand polypharmacology in GPCRs and kinases (Figure S1).

**Table 1:** Statistics of all known chemical-protein interaction pairs of the training set and validation set used in this study.

Data Sets	Targets	N_c_	N_p_	N_i_	N_dc_	N_dp_	Sparsity (%)
Training Set	GPCRs	4,741	97	17,111	3.61	176.4	3.72
	Kinases	2,827	206	13,648	4.83	66.3	2.34
Validation Set	GPCRs	92	46	271	2.95	5.89	6.40
	Kinases	188	28	202	1.07	7.21	3.84

N_c_: The number of compounds, N_p_: The number of proteins, N_i_: The number of chemical-protein interactions, N_dc_: The average degree of compound nodes, N_dp_: The average degree of protein nodes.

### Relationships among Several Similarities

The heat maps of the compound-compound 2D structural similarity (Tanimoto-scores) and protein sequence similarity (Smith-Waterman scores) were given in Figure S2. The mean values of 2D Tanimoto-scores were 0.458 and 0.444 for ligands of GPCR and kinase, respectively. The range of Smith-Waterman scores of GPCRs was from 0.423 to 0.914 with a mean of 0.517. The range of Smith-Waterman scores of kinases was from 0.383 to 0.996 with a mean of 0.517. From Figure S2, there was diverse ligand chemical space and target coverage of GPCRs and kinases.

**Figure 2:**
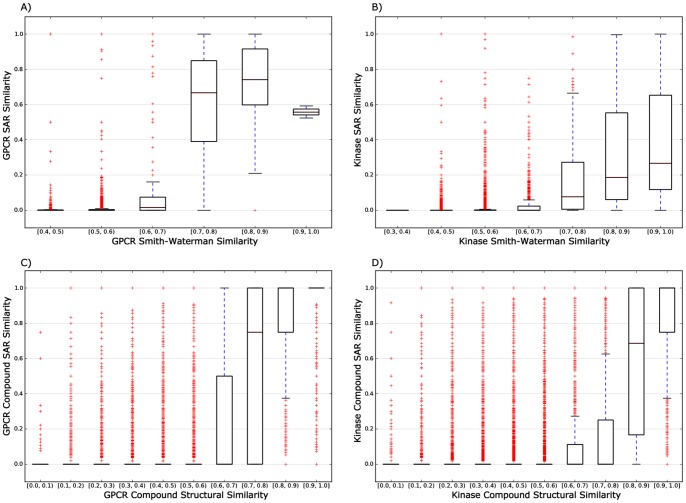
Box plots of compound-compound and protein-protein similarities against compound or protein structure activity-relationship (SAR) similarities. (**A**) protein-protein (GPCRs) sequence similarity (Smith-Waterman scores) against GPCRs SAR similarity, (**B**) protein-protein (kinases) sequence similarities (Smith-Waterman scores) against kinases SAR similarity, (**C**) compound-compound (GPCR ligands) structural similarities (Tanimoto scores) against the GPCR ligands SAR similarities and (**D**) compound-compound (kinase ligands) structural similarity (Tanimoto scores) against kinase ligands SAR similarities.

We also calculated compound structural activity-relationships (SAR) similarity scores 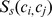 and protein SAR scores 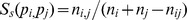 using the Tanimoto-score metric, where 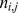 is the number of proteins or chemicals that interact with both 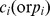 and 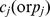, 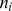 is the degree of 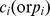, and 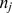 is the degree of 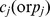. The further description about SAR similarity scores was given in Bamborough *et al*. [32]. Figure 2 gave the distribution of compound 2D structural similarities and protein domain sequence similarities against compound and protein SAR similarities. From the box plots, we found two useful features. First, when the compound-compound pairs have the higher Tanimoto scores, they have the higher compound SAR similarities. This feature confirmed the common hypothesis that two ligands with similar structure have the similar biological spectrum [33]. Second, when the protein-protein pairs have the higher Smith-Waterman scores, they have the higher protein SAR similarities. The second feature confirmed the common hypothesis that two proteins with similar structural, functional or evolutionary features will have the similar biological function and bind with similar ligands [34].

### Performance of the Proposed Methods

#### Unweighted Network-based Inference (NBI)

The unweighted NBI method only considers the binary CPI pairs among chemical and protein nodes (Figure 1C). The performance for the test set by 10 simulation times test was summarized in Table 2. The high AUC value of 0.981±0.001 and 0.976±0.002 were yielded for test sets of GPCRs and kinases, respectively. The performance of NBI method was significantly higher than that of the DBSI and TBSI methods, which is in agreement with our previous work [26]. In this study, four similarity metrics, namely Tanimoto, Cosine, Forbes and Russell-rao were systematically investigated. The overall performance of Tanimoto was marginally higher than that of Cosine, Forbes and Russell-rao. Comparing the DBSI and TBSI method (Table 2), the performance of the TBSI method was better than that of the DBSI method, when prioritizing new candidate proteins to a given chemical. The performance of the DBSI method was better than that of the TBSI method, when prioritizing new candidate chemicals to a given protein.

**Table 2:** The performance of the test set of GPCRs and kinases using different methods by 10 simulation times test of 10-fold cross validation.

Target	Methods	C_i_ (P_a_, P_b_, …, P_m_)			P_j_ (C_a_, C_b_, …, C_n_)		
		R	ER	AUC	R	ER	AUC
GPCRs	NBI	0.969±0.004*	18.8±0.086	0.981±0.001	0.285±0.022	270.5±21.1	0.972±0.002
	NWNBI	0.974±0.004	18.9±0.070	0.981±0.001	0.285±0.022	270.5±21.1	0.972±0.002
	EWNBI	0.970±0.004	18.8±0.072	0.981±0.001	0.283±0.028	268.4±26.6	0.972±0.002
	DBSI-T	0.488±0.014	9.48±0.263	0.885±0.002	0.215±0.037	203.7±35.1	0.885±0.004
	DBSI-C	0.458±0.011	8.88±0.213	0.879±0.002	0.159±0.042	151.0±39.6	0.874±0.004
	DBSI-F	0.476±0.013	9.23±0.260	0.880±0.002	0.158±0.040	149.9±37.9	0.874±0.004
	DBSI-R	0.427±0.011	8.27±0.222	0.879±0.002	0.169±0.035	160.1±33.1	0.874±0.004
	TBSI	0.907±0.003	17.6±0.064	0.969±0.001	0.035±0.014	33.51±13.1	0.570±0.007
Kinases	NBI	0.863±0.007	35.5±0.302	0.976±0.002	0.380±0.022	215.0±12.4	0.958±0.001
	NWNBI	0.877±0.007	36.1±0.294	0.977±0.002	0.380±0.022	215.0±12.4	0.958±0.001
	EWNBI	0.866±0.010	35.7±0.397	0.976±0.002	0.360±0.025	203.8±13.9	0.955±0.002
	DBSI-T	0.326±0.015	13.4±0.627	0.878±0.003	0.205±0.022	115.8±12.7	0.846±0.006
	DBSI-C	0.303±0.016	12.5±0.642	0.872±0.003	0.141±0.016	79.8±9.3	0.826±0.007
	DBSI-F	0.273±0.011	11.3±0.465	0.872±0.003	0.137±0.016	77.7±9.0	0.825±0.007
	DBSI-R	0.280±0.014	11.5±0.587	0.872±0.003	0.140±0.016	78.9±9.3	0.827±0.007
	TBSI	0.645±0.007	26.6±0.289	0.908±0.005	0.061±0.011	34.5±6.0	0.660±0.008

All performances were evaluated based on top 5 predicted lists. NBI, network-based inference; NWNBI, node weighted network-based inference; EWNBI, edge weighted network-based inference; DBSI-T, drug-based similarity inference with Tanimoto similarity score; DBSI-C, DBSI with Cosine similarity score; DBSI-F, DBSI with Forbes similarity score; DBSI-R, DBSI with Russell-rao similarity score; TBSI, target-based similarity inference; R, recall; ER, recall enhancement; AUC, the area under the receiver operating characteristic curve; C_i_ (P_a_, P_b_, …, P_m_) represents the prioritization of new targets for a given chemical; P_j_ (C_a_, C_b_, …, C_n_) represents the prioritization of new chemicals for a given protein. *The standard deviation of the performance measured by 10 independent simulation times test of 10-fold cross validation.

#### Node Weighted Network-based Inference (NWNBI)

**Figure 3:**
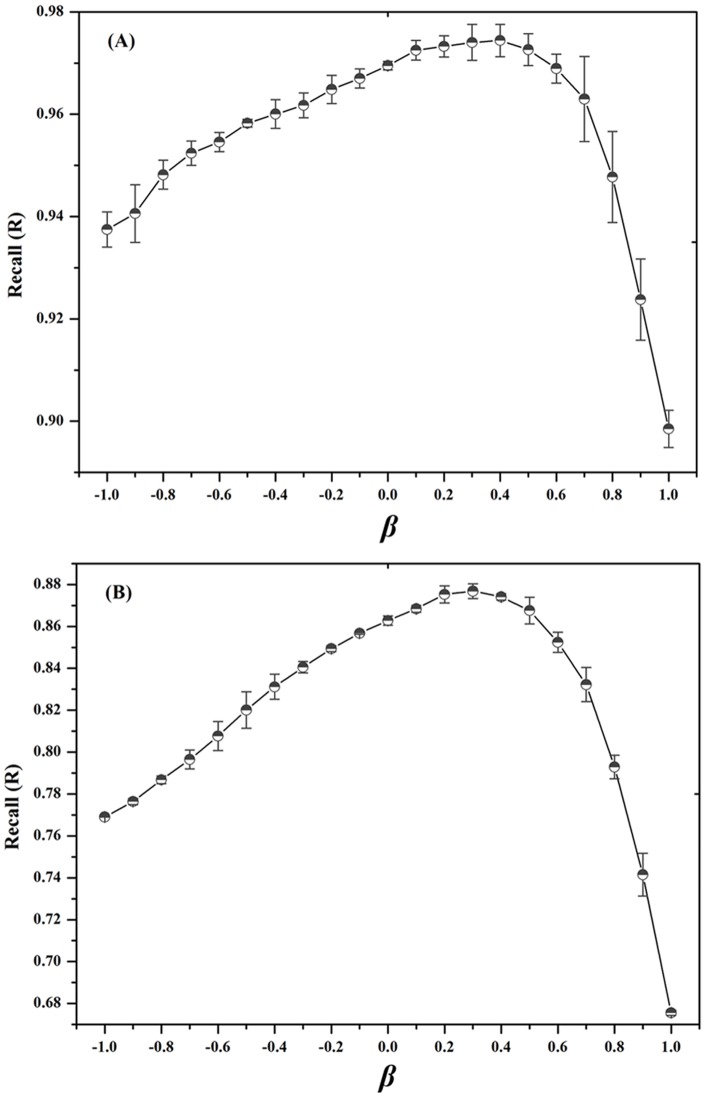
Recall metric of the parameter *β* on the node weighted network-based inference method for test set when assessed the top five predicted candidate lists. The recall reaches its maximum value at about 0.4 and 0.3 for GPCRs (**A**) and kinases (**B**), respectively. The error bars denote the standard deviation by 10 times independent simulation test.

As shown in Table 2, the performance of NWNBI was marginally higher than that of the unweighted NBI method. For example, the R value of 0.974 using NWNBI method was marginally higher than 0.969 using unweighted NBI method evaluated on top 5 predicted lists. Figure 3 showed that the performance of test set by simulation 10 times reaches its maximum value at about *β* = 0.3 for both GPCRs and kinases. Compared with the uniform case of *β* = 0, a positive *β* strengthens the influence of hub nodes of chemical or protein, while a negative *β* weakens the influence of hub nodes. The results indicated that an appropriate increase of the initial resource located on popular proteins can marginally improve the predictive accuracy of NBI method.

#### Edge Weighted Network-based Inference (EWNBI)

In this study, the edges of CPI network are weighted by the potency (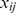) of binding affinity (*K_i_*) or inhibitory activity (*IC_50_*) of the real physical interactions among the chemical and protein nodes (Figure 1D). As given in Table 2, the performance of EWNBI was marginally worse than the unweighted NBI, which is broadly consistent with the strength of the weak ties hypothesis in biochemical network [35].

#### Role of Weak Chemical-Protein Interactions

To further explore the role of weak interactions in CPI bipartite network, we introduce an exponent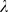:
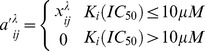
(5)


In EWNBI, when a node *i* allocates its resource to two nodes *p* and *q*, the ratio of resource *p* and *q* received is 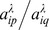. When 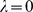, it is the unweighted NBI method; when 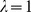, it is the EWNBI method. When 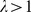, it positively strengthens the weighted value of strong CPI edges (high potency between chemical and protein nodes), while 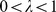 positively strengthens the weighted value of weak CPI edges (low potency between chemical and protein nodes). Otherwise, a negative 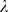 gives the negative effects.

**Figure 4:**
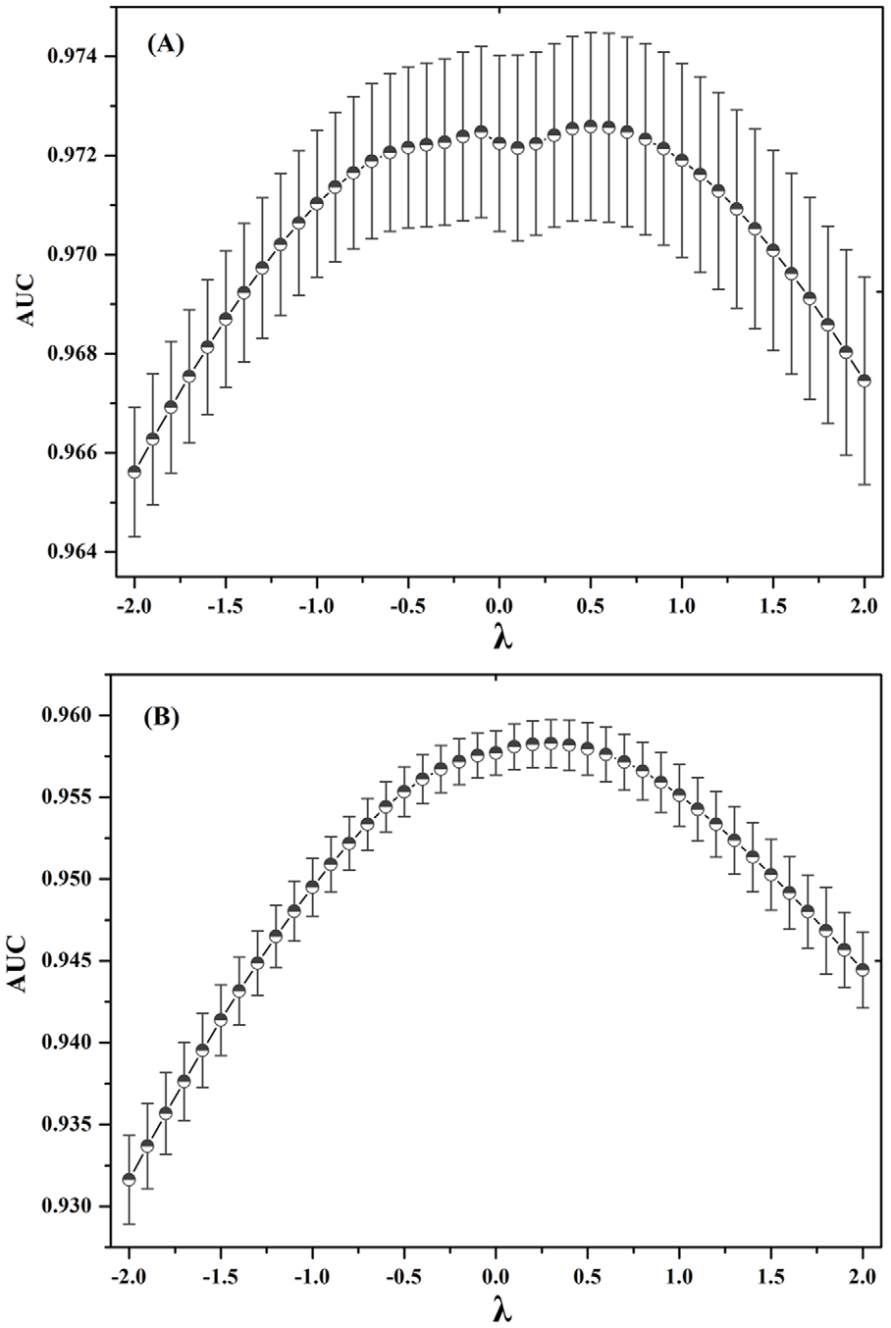
Analysis of the role of weak chemical-protein interactions by exponent *λ*. When 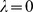, it is unweighted NBI method; when 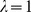, it is the EWNBI method. When 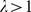, it positively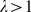 strengthens the weighted value of strong CPI edges, while 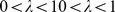 positively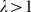 strengthens the weighted value of weak CPI edges. Otherwise, a negative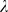 will give the negative effects. The area under receiver operating characteristic curve (AUC) was yielded for test set by simulation 10 times test, the error bar denotes the standard deviation. GPCRs (**A**) and kinases (**B**).

As shown in Figure 4, the AUC increases by λ increasing when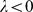. The AUC decreases by λ increasing when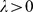. The highest AUC were yielded when λ = 0.50 and 0.25 for GPCRs and kinases, respectively. The results indicated that the weak interactions could play an important role in CPI prediction in the real weighted CPI network, which is in agreement with the weak ties hypothesis in some real network, such as US air transportation network [36], the neural network of the nematode worm C. elegans [36], the co-authorship network [36], social networks [37] and biochemical network [35] etc. Although it is well-known that weak links hypothesis is very important for complex network, this result is the first confirmation in the real CPI network.

### Prediction of Novel Chemical-Protein Interactions

Although the NBI method can yield high predictive performance, there was a defect that the NBI method cannot predict general chemicals or proteins which did not have any initial links in the training set [26]. In this study, we resolved this bottleneck by integrating the NBI and DBSI methods. Ninety-two novel FDA approved and experimental drugs targeting 46 known GPCRs, and 188 novel approved and experimental drugs targeting 28 known kinases (designated as the external validation sets in Table S3) were collected from DrugBank [38] and KEGG [39], which CPI pairs did not include in the training set (Table 1). Before prioritizing new candidate proteins for a novel chemical (designated compound **A**) using NBI method, we constructed a new initial virtual resources for compound **A** as follows: (i) Calculate the Tanimoto similarity between compound **A** and each compound in the training set; (ii) Displace the topology CPI links of a compound with the highest Tanimoto similarity score in the training set for compound **A**; (iii) Then the candidate proteins were prioritized for compound **A** using the new constructed virtual CPI bipartite network. As summarized in Table 3, the reasonable predictive accuracies were yielded. For GPCRs, the AUC value of 0.77 was yielded when prioritizing candidate targets to a given novel drug using the NBI method, which higher than 0.74 using DBSI method. For 188 drugs of kinase, the AUC value of NBI method was about 0.83, which was marginally lower than DBSI method. The possible reason is that the CPI network of kinases was too sparse, as the average degree of 188 drugs of kinase was only 1.07 (Table 1). In order to assess the reliability of the gold standard data to determine whether the good results might be based on very similar homologous relationships between compounds and similar compounds, we re-evaluated the generalization ability of our methods based on the new validation set after removing 50% high similar compounds with top Tanimoto scores using MACCS keys on the original external validation set (Table S3). As showed in Table S4, the reasonable high performance was also yielded for the new validation set after removing high similar compounds.

**Table 3:** The performance of difference inference methods in the external validation set of GPCRs and kinases.

Tragets	Methods	C_i_ (P_a_, P_b_, …, P_m_)			P_j_ (C_a_, C_b_, …, C_n_)		
		R	ER	AUC	R	ER	AUC
GPCRs	NBI	0.535	2.60	0.769	0.684	3.15	0.693
	NWNBI	0.559	2.72	0.756	0.684	3.15	0.693
	EWNBI	0.561	2.72	0.764	0.697	3.21	0.691
	DBSI-T	0.470	2.28	0.743	0.603	2.77	0.685
	DBSI-C	0.472	2.29	0.739	0.604	2.78	0.684
	DBSI-F	0.473	2.29	0.739	0.612	2.82	0.686
	DBSI-R	0.473	2.30	0.741	0.610	2.81	0.683
	TBSI	0.342	1.66	0.639	0.361	1.66	0.593
Kinases	NBI	0.502	5.17	0.828	0.222	2.09	0.607
	NWNBI	0.427	4.40	0.812	0.222	2.09	0.607
	EWNBI	0.459	4.73	0.821	0.159	1.50	0.597
	DBSI-T	0.594	6.11	0.847	0.188	1.77	0.573
	DBSI-C	0.588	6.06	0.847	0.148	1.39	0.564
	DBSI-F	0.595	6.12	0.846	0.142	1.33	0.563
	DBSI-R	0.583	6.00	0.846	0.146	1.38	0.563
	TBSI	0.061	0.62	0.326	0.082	0.775	0.510

All performances were evaluated based on top 20 predicted lists. NBI, network-based inference; NWNBI, node weighted network-based inference; EWNBI, edge weighted network-based inference; DBSI-T, drug-based similarity inference with Tanimoto similarity score; DBSI-C, DBSI with Cosine similarity score; DBSI-F, DBSI with Forbes similarity score; DBSI-R, DBSI with Russell-rao similarity score; TBSI, target-based similarity inference; R, recall; ER, recall enhancement; AUC, the area under the receiver operating characteristic curve; C_i_ (P_a_, P_b_, …, P_m_) represents the prioritization of new targets for a given chemical; P_j_ (C_a_, C_b_, …, C_n_) represents the prioritization of new chemicals for a given protein.

### Cast Studies

**Figure 5:**
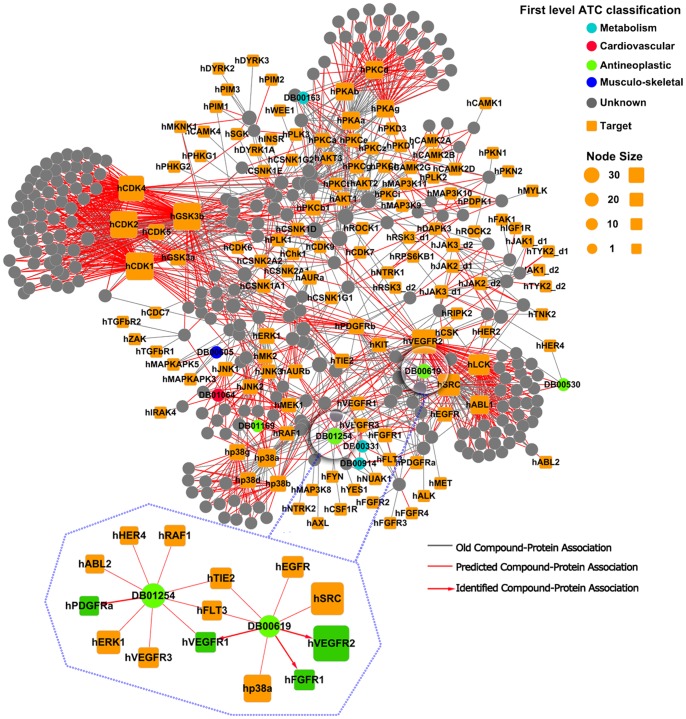
Discovered chemical-protein interactions (CPI) bipartite networks among 267 FDA approved or experimental drugs and 130 kinases. Circle and square nodes correspond to drugs and kinases, respectively. A gray line represents the old CPI annotated in the DrugBank and KEGG. The red line represents the predicted CPI. The red arrow line represents the new predicted CPI which is validated by literatures. The size of the drug node is the fraction of the number of targets that the drug linked. The size of the target node is the fraction of the number of drugs that the target linked. Color codes are given in the legend. Drug nodes (circles) are colored according to their Anatomical Therapeutic Chemical Classification. This graph and Figure 6 were prepared by Cytoscape (http://www.cytoscape.org/).

**Figure 6:**
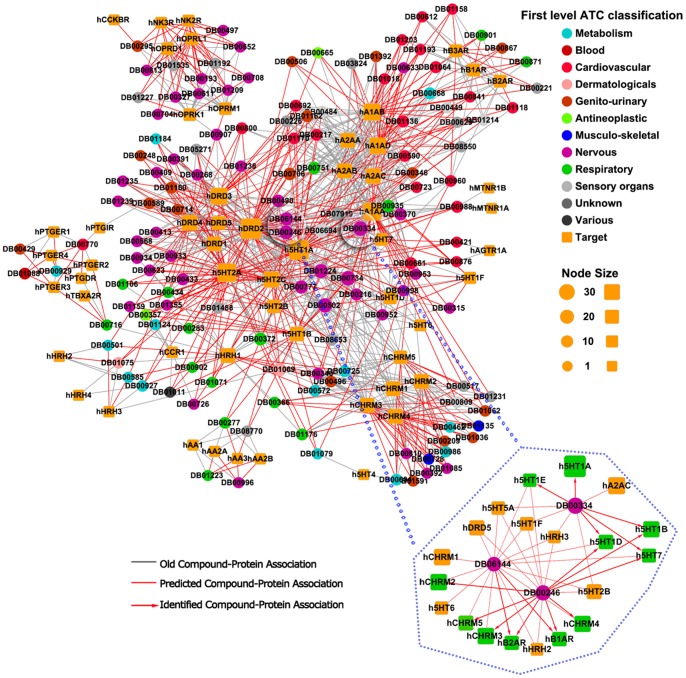
Discovered chemical-protein interaction (CPI) bipartite network among 139 FDA approved or experimental drugs and 55 GPCRs (Table S4). Circle and square nodes correspond to drugs and GPCRs, respectively. The definition of nodes and edges were given in the caption of Figure 5.

In order to test the real predictive ability of our method, we prioritized all candidate CPIs for known ligands or proteins using the unweighted NBI method by combining the training sets and external validation sets. About 183 thousands of candidate CPI pairs among 4833 known ligands (including 139 FDA approved or experimental drugs) and 97 GPCRs were predicted. About 415 thousands of candidate CPI pairs among 3015 known ligands (including 267 FDA approved or experimental drugs) and 206 kinases were predicted. All predicted CPI lists can be downloaded from web sites: http://www.lmmd.org/database/cpi/ for further experimental investigation. Two known and predicted CPI bipartite networks were constructed using Cytoscape (http://www.cytoscape.org/) in Figures 5 and 6. Due to space limit, we only investigated the predicted targets for five known drugs, namely imatinib, dasatinib, sertindole, olanzapine and ziprasidone. And the molecular hypotheses and experimental evidences of predictions were provided (Table S5).

**Imatinib** (DB00619) is an ATP-competitive selective inhibitor of Bcr-Abl used to treat chronic myelogenous leukemia (CML), gastrointestinal stromal tumors and a number of other malignancies. In ChEMBL, imatinib (Compound_ID 7083) targeted 9 known kinases, namely hABL_1_, hABL_2_, hPDGFR_a_, hPDGFR_b_, hLCK, hKIT, hLYN, hCSF_1R_ and hSYK with IC_50_ less than 10 μM (Figure 5) [27]. As given in Table S5, among top 16 predicted kinases of imatinib, 9 ones were predicted correctly with a successful hit rate of 56.3%. Interestingly, seven new targets, namely hVEGFR_1_, hVEGFR_2_, hSRC, hEGFR, hFGFR_1_, hFLT_3_, and hTIE_2_, were also predicted for imatinib with high scores. Among them, hVEGFR_2_ was predicted with the highest score at 0.812. Deininger *et al*. reported that the IC_50_ values of imatinib were 31.2 μM, 19.5 μM and 10.7 μM for hFGFR_1_, hVEGFR_1_ and hVEGFR_2_, respectively [40]. Our prediction was consistent with literatures.

**Dasatinib** (DB01254) is a novel oral dual, multi-target tyrosine kinase inhibitor, which was approved for chronic myelogenous leukemia treatment. In ChEMBL, dasatinib (Compound_ID 12304) targeted 19 known kinases, namely hVEGFR_2_, hp38a, hp38b, hp38d, hp38g, hSRC, hEGFR, hPDGFR_b_, hFGFR_1_, hLCK, hKIT, hABL_1_, hHER_2_, hMEK_1_, hMEK_2_, hFYN, hYES_1_, hCSF_1R_ and hEPHA_2_ with the IC_50_ value less than 10 μM. As given in Table S5, on top 27 predicted kinases of dasatinib, 19 targets were predicted correctly with a hit successful rate of 70.4%. Seven new kinases of hVEGFR_1_, hVEGFR_3_, hTIE_2_, hFLT_3_, hPDGFR_a_, hRAF_1R_, hABL_2_, and hHER_4_ were predicted to bind with dasatinib with high scores. Lombardo *et al.* demonstrated that dasatinib inhibit PDGFR *in vitro* with an IC_50_ of 28 nM [41]. Chen *et al.* reported that dasatinib is a potent inhibitor of PDGFR *via* cell-based assay [42]. Quintas-Cardama *et al.* reported that dasatinib effectively inhibited several SRC family kinases, including SRC (IC_50_ = 0.55 nM), LCK ((IC_50_ = 1.1 nM), FYN (IC_50_ = 0.2 nM) and YES (IC_50_ = 0.41 nM) [43]. The data indicated that our predicted results are in agreement with literatures.

**Sertindole** (DB06144) is an oral antipsychotic drug targeted with dopamine D_2_, serotonin 5-HT_2A_ and 5-HT_2C_, and α_1_-adrenoreceptors. The clinical trails have confirmed that sertindole is effective at a low dopamine D_2_ occupancy level. In the ChEMBL, sertindole (Compound_ID 85092) targeted 15 known GPCRs, namely DRD_1_, DRD_2_, DRD_3_, DRD_4_, A_1AA_, A_1AB_, A_1AD_, A_2AA_, A_2AC_, 5HT_1A_, 5HT_1B_, 5HT_2A_, 5HT_2C_, A_2AB_ and HRH_1_ with K_i_ value less than 10 μM (Figure 6). As given in Table S5, in top 16 predicted GPCRs of sertindole, 15 receptors were predicted correctly with a hit successful rate of 93.8%. Sertindole was first marketed in 1996 in several European countries. However, it was withdraw two years later because of numerous cardiac adverse effects such as QTc prolongation and the UK database adverse drug reactions information tracking reported that the rate of arrhythmias or sudden death was almost 10-times greater for sertindole than for olanzapine and risperidone [44]. The molecular mechanism of side effects of sertindole was unknown. As given in Figure 6, sertindole was predicted to bind with hB_1AR_ and hB_2AR_, which were consistent with literature [45].

**Olanzapine** (DB00334) approved in 1996, is an atypical antipsychotic agent, which is used to treat both negative and positive symptoms of schizophrenia, acute mania with bipolar disorder, agitation, and psychotic symptoms in dementia [38], [46]. Olanzapine mainly targeted with dopamine, histamine H_1_, muscarinic, 5-HT_2_ and α_1_-adrenoreceptors with high binding affinities. In the ChEMBL, olanzapine promiscuously targeted 22 known GPCRs, namely hDRD_1_, hDRD_2_, hDRD_3_, hDRD_4_, hDRD_5_, hCHRM_1_, hCHRM_2_, hCHRM_3_, hCHRM_4_, hCHRM_5_, hA_1AB_, hA_1AA_, hA_1AD_, hA_2AA_, hA_2AB_, hB_1AR_, hB_2AR_, hB_3AR_, h5HT_2A_, h5HT_2C_, h5HT_6_ and hHRH_1_ (Figure 6). As given in Table S5, in top 26 predicted GPCRs of olanzapine, 22 targets were predicted correctly with a hit successful rate of 84.6%. The receptors of hA_2AC_, h5HT_1A_, h5HT_1B_, h5HT_1D_, h5HT_1E_, h5HT_2B_, h5HT_7_, hHRH_2_ and hHRH_3_ were predicted to have novel interactions with olanzapine (Figure 6). Recently, several results reported that olanzapine can bind with the receptors of h5HT_1A_, h5HT_1B_, h5HT_1D_, h5HT_1E_ and h5HT_7_ with high binding affinities [45], [47].

**Ziprasidone** (DB00246) is a selective monoaminergic antagonist with high affinity for the serotonin Type (5HT_1A_, 5HT_2_), dopamine D_2_ and H_1_ histaminergic receptors. It is a psychotropic agent indicated for the treatment of schizophrenia. In the ChEMBL, ziprasidone (Compound_ID 89351) targeted 16 known GPCRs, namely hDRD_1_, hDRD_2_, hDRD_3_, hDRD_4_, hA_1AA_, hA_1AD_, hA_2AA_, hA_2AB_, hA_2AC_, h5HT_1A_, h5HT_2A_, h5HT_2B_, h5HT_2C_, h5HT_6_, hHRH_1_ and hCHRM_1_ with K_i_ value less than 10 μM (Figure 6). As given in Table S5, in top 20 predicted GPCRs of ziprasidone, 16 targets were predicted correctly with a hit successful rate of 80%. The receptors of hDRD_5_, h5HT_1B_, h5HT_1D_, h5HT_7_, hCHRM_2_, hCHRM_3_, hCHRM_4_, hCHRM_6_, hB_1AR_ and hB_2AR_ were predicted to have new indications with ziprasidone. Recently, several results reported that ziprasidone can bind with the receptors of h5HT_1B_, h5HT_1D_, h5HT_7_, hCHRM_2_, hCHRM_3_, hCHRM_4_, hCHRM_6_, hB_1AR_ and hB_2AR_ with the high binding affinities [45], [47], which demonstrated the feasibility of our methods to prioritize new target to known drugs.

## Discussion

### Potential Application of Our Methods

Herein, we systematically investigated the utility of unweighted and weighted network-based inference method in prediction of new targets for old drugs or general ligands. The proposed method achieved the AUC was about 0.98 and 0.83 for the test set and the external validation set, respectively. Today, the increased availability of large scale open access resources on bioactivities of small molecules has a significant impact on pharmacology facilitated [48]. Therefore, our methods could provide a fast and effective strategy to digest the vast amounts of data for CPI prediction and drug repositioning.

The method proposed in this study fall within the scope of the emerging field of systems pharmacology [1]. Recently, systems pharmacology approaches have been applied successfully to various problems, such as drug repositioning [1], [26]. Herein, we extended our previous work on developing two different weighted NBI, namely EWNBI and NWNBI for CPI prediction and drug repositioning. We found that NWNBI method was marginally higher than NBI with an appropriate parameter optimization. And the weak interactions hypothesis was first proposed in CPI network by EWNBI method. To our knowledge, our method could be used in several biological relevant directions, such as gene-disease association prediction [49], drug-diseases association prediction (drug adverse events prediction) [50] etc. by integrating meta-biochemical networks in the further.

### Polypharmacology of Ligands

The resistance of anti-cancer drugs is a large challenge for cancer therapeutics [51]. Overcoming the resistance mechanisms may require targeting tumor cells at promiscuous levels, through either single drugs binding with the multi-targets or cocktails of several highly selective inhibitors [52]. A big bottleneck for the cancer research community is how to decipher chemical-protein interactome, how to optimize the best combinations of targets and then prioritize those combinations for clinical testing. The polypharmacology of kinase ligands was encouraged by our results (Figure 5) that the emerging class of well-tolerated kinase inhibitors of imatinib and dasatinib, exhibit the multi-target on kinases and are less selective than initially findings [53]. Today, more than 800 drugs are in clinical development for cancer indications and the current success rate in bringing drugs to the markets remains only in the range of 5–8% [2]. Most of drugs which selectively targeted kinases with high potency *in vitro* models are failure in the late stage of clinical trails due to side effects and lacking *in vivo* activities [54], [55]. As shown in Figure 5, most of experimental drugs of kinases (gray circle nodes) in DrugBank were predicted to have a significant promiscuity. These results could be useful for finding new usages of some failure of drugs and multi-target anticancer drugs design. For example, imatinib was initially approved for the treatment of CML, but it was tested in five patients with hypereosinophilic syndrome [56].

The features of polypharmacology are not restricted to the kinases inhibitors. Anti-psychotics drugs, such as sertindole, olanzapine and ziprasidone also promiscuously targeted with GPCRs rather than individual one. As given in Figure 6, the therapeutic effects of sertindole, olanzapine and ziprasidone mainly targeted three dopaminergic (D_1_, D_2_ and D_3_) and three serotoninergic (5-HT_1A_, 5-HT_1D_ and 5-HT_2A_) receptors. But they often lead to several side effects by binding with adrenergic and histaminergic receptors, such as QTc prolongation [45]. In this study, it is worth acknowledged that NBI methods can effectively help to prioritize the new candidate CPI, decipher potential molecular mechanism of off-targets and drug repositioning.

### Weak Interactions between Chemicals and Proteins

Herein, for the first time we identified the evidence of weak interactions in CPI network. In fact, multiple weak interactions cannot be ignored in polypharmacological actions [57]. It is estimated that most (more than 80%) of the cellular proteins, signaling and transcriptional networks are in a low-affinity or transient “weak linkage” with each other [58]. Weak physical interactions with low binding affinity play critical roles in molecular recognition among biological systems, from the classic example of protein folding to recent discoveries in metabolism, gene regulation and signal transduction [59]. For example, the binding affinity between enzyme and the alternative substrate is usually low [60]. The hypothesis of weak interactions for drug therapeutics had been applied for more than two thousand years in Chinese Traditional Medicine [61]. A drug with low affinity and multi-target may have high therapeutic value with fewer side effects than one with high affinity and single target. For example, sorafenib was designed as a potent nanomolar (nM) inhibitor of BRAF which a protein implicated in the survival of melanoma cells. Unfortunately, it failed in clinical trial due to its low anti-melanoma efficacy [62]. In contrast to, low affinity and multi-target noncompetitive NMDA receptor antagonists developed for treatment of Alzheimer's disease, may have fewer side effects than some high affinity and single target drugs [63]. The detailed assessment of weak CPI interaction is a hot topic in drug discovery and complex network, but it was beyond the range of this article. Our groups are actively investigating this important issue.

### Conclusions

In summary, we proposed two different weighted NBI methods for CPI prediction. The high performance was yielded using our methods. Comparing with conventional ligand and receptor-based methods, NBI method only used CPI network topology similarity by simultaneously exploiting both topological and functional modularity to prioritize new targets for a given drug or prioritize new drugs for a given target, which did not need any 2D or 3D structural information of targets and drugs. Our methods will generate a set of predicted candidate miss linked CPI. The biologist can then follow up on the new high scoring CPI for further experimental assay. Therefore, our methods open new avenue for CPI identification.

The weak links hypothesis had been proposed in several biochemical networks and social network etc. In this study, the weak links hypothesis in CPI network was first proposed by EWNBI method. Enhance and diminish stronger or weaker CPI edges all decreased the predict accuracy. The maximum predictive accuracy was yielded when stronger and weaker CPI edges achieved a balance. These computational polypharmacology perspectives could let people beef up efforts for CPI prediction and drug repositioning.

## Supporting Information

Figure S1 **The degree distributions of chemical and protein (GPCRs and Kinases) nodes in two comprehensive chemical-protein interactions bipartite networks.**
(TIF)

Figure S2 **The heat maps of the chemical similarities and protein sequence similarities.** **A**) Tanimoto similarity of 4,741 GPCRs ligands, **B**) Tanimoto similarity of 2,827 kinase ligands, **C**) Genomic sequence Smith-Waterman similarity of 97 GPCRs, **D**) Genomic sequence Smith-Waterman similarity of 206 kinases.
(TIF)

Table S1 **The detailed description of data sets using in this study.** The Compound ID, Target ID, SIMLES, K_i_ and IC_50_ value of 17,100 chemical-protein interaction pairs among 4,741 compounds and 97 G protein-coupled receptors (GPCRs), and 13,600 CPI pairs among 2,827 compounds and 206 kinases.
(XLS)

Table S2 **The sequences in FASTA format of 97 G protein-coupled receptors and 206 kinases extracted from ChEMBL database.**
(DOC)

Table S3 **The detailed description of the external validation set.** The external validation set of 92 novel FDA approved and experimental drugs targeting 46 GPCRs, and 188 novel approved and experimental drugs targeting 28 kinases collected from DrugBank and KEGG.
(XLS)

Table S4 **The performance of difference inference methods on the new external validation set.** The new external validation set was constructed after removing 50% high similar compounds with top Tanimoto similarity using MACCS keys on the original external validation set (Table S3) of GPCRs and kinases.
(PDF)

Table S5 **The molecular hypothesis, experimental evidence and predicted target list for five known drugs, namely imatinib, dasatinib, sertindole, olanzapine and ziprasidone.**
(XLS)
