# Supplementary figures and images for: Prediction of Chemical-Protein Interactions Network with Weighted Network-Based Inference Method
Source: PLoS One. 2012 Jul 16;7(7):e41064. doi: 10.1371/journal.pone.0041064 (PMC3397956; doi:10.1371/journal.pone.0041064)

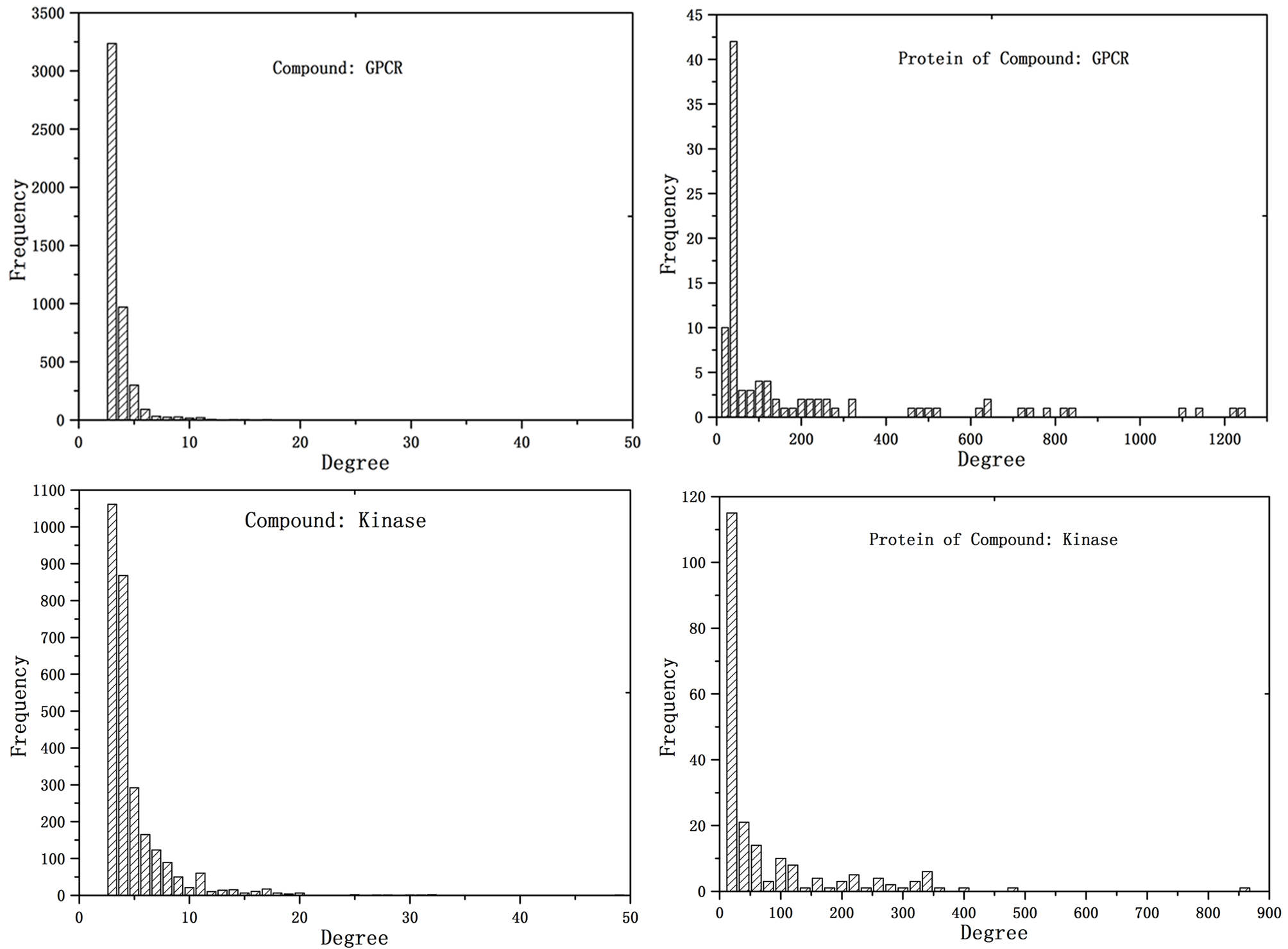

Supplement: Figure S1 — The degree distributions of chemical and protein (GPCRs and Kinases) nodes in two comprehensive chemical-protein interactions bipartite networks. (TIF) [file pone.0041064.s001.tif]

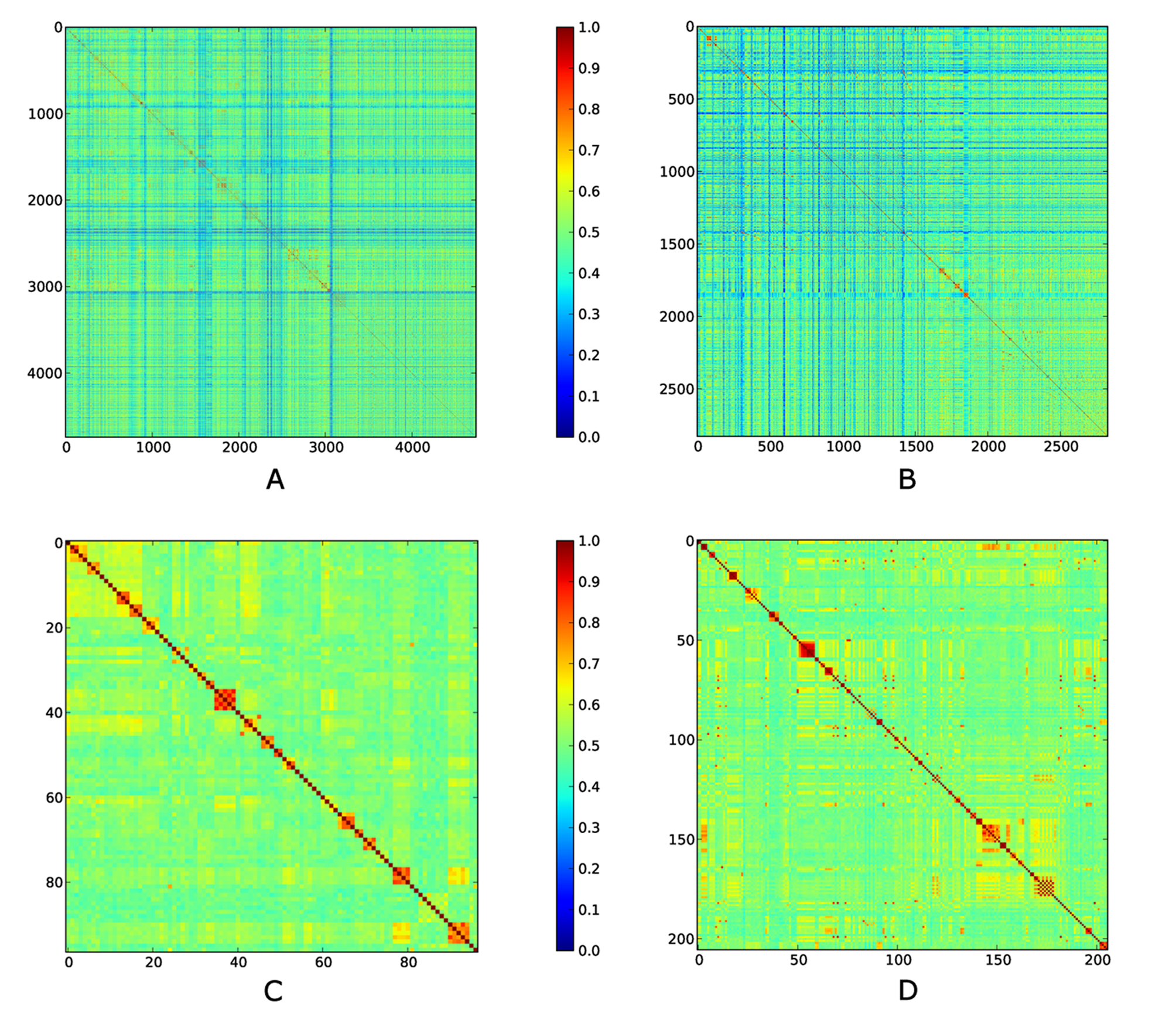

Supplement: Figure S2 — The heat maps of the chemical similarities and protein sequence similarities. A) Tanimoto similarity of 4,741 GPCRs ligands, B) Tanimoto similarity of 2,827 kinase ligands, C) Genomic sequence Smith-Waterman similarity of 97 GPCRs, D) Genomic sequence Smith-Waterman similarity of 206 kinases. (TIF) [file pone.0041064.s002.tif]
