# Supplementary material for: Prediction of Chemical-Protein Interactions Network with Weighted Network-Based Inference Method
Source: PLoS One. 2012 Jul 16;7(7):e41064. doi: 10.1371/journal.pone.0041064 (PMC3397956; doi:10.1371/journal.pone.0041064)
Supplement: Table S2 — The sequences in FASTA format of 97 G protein-coupled receptors and 206 kinases extracted from ChEMBL database. (DOC) [file pone.0041064.s004.doc]

Table S2. The sequences in FASTA format of 97 G protein-coupled receptors and 206 kinases extracted from ChEMBL database.

**GPCRs**

>h5HT1A_89

TGISDVTVSYQVITSLLLGTLIFCAVLGNACVVAAIALERSLQNVANYLIGSLAVTDLMV

SVLVLPMAALYQVLNKWTLGQVTCDLFIALDVLCCTSSILHLCAIALDRYWAITDPIDYV

NKRTPRRAAALISLTWLIGFLISIPPMLGWRTPEDRSDPDACTISKDHGYTIYSTFGAFY

IPLLLMLVLYGRIFRAARFRIRKTVKKVEKTGADTRHGASPAPAEAKRKMALARERKTVK

TLGIIMGTFILCWLPFFIVALVLPFCESSCHMPTLLGAIINWLGYSNSLLNPVIYAYFNK

DFQNAFKKIIKCKFCRQ

>h5HT1B_124

IYQDSISLPWKVLLVMLLALITLATTLSNAFVIATVYRTRKLHTPANYLIASLAVTDLLV

SILVMPISTMYTVTGRWTLGQVVCDFWLSSDITCCTASILHLCVIALDRYWAITDAVEYS

AKRTPKRAAVMIALVWVFSISISLPPFFWRQAKAEEEVSECVVNTDHILYTVYSTVGAFY

FPTLLLIALYGRIYVEARSRILKQTPNRTGKRLTRAQLITDSPGSTSSVTSINSRVPDVP

SESGSPVYVNQVKVRVSDALLEKKKLMAARERKATKTLGIILGAFIVCWLPFFIISLVMP

ICKDACWFHLAIFDFFTWLGYLNSLINPIIYTMSNEDFKQAFHKLIRFKCTS

>h5HT1D_123

AWDPRTLQALKISLAVVLSVITLATVLSNAFVLTTILLTRKLHTPANYLIGSLATTDLLV

SILVMPISIAYTITHTWNFGQILCDIWLSSDITCCTASILHLCVIALDRYWAITDALEYS

KRRTAGHAATMIAIVWAISICISIPPLFWRQAKAQEEMSDCLVNTSQISYTIYSTCGAFY

IPSVLLIILYGRIYRAARNRILNPPSLYGKRFTTAHLITGSAGSSLCSLNSSLHEGHSHS

AGSPLFFNHVKIKLADSALERKRISAARERKATKILGIILGAFIICWLPFFVVSLVLPIC

RDSCWIHPALFDFFTWLGYLNSLINPIIYTVFNEEFRQAFQKIVPFRKAS

>h5HT1E_128

MNITNCTTEASMAIRPKTITEKMLICMTLVVITTLTTLLNLAVIMAIGTTKKLHQPANYL

ICSLAVTDLLVAVLVMPLSIIYIVMDRWKLGYFLCEVWLSVDMTCCTCSILHLCVIALDR

YWAITNAIEYARKRTAKRAALMILTVWTISIFISMPPLFWRSHRRLSPPPSQCTIQHDHV

IYTIYSTLGAFYIPLTLILILYYRIYHAAKSLYQKRGSSRHLSNRSTDSQNSFASCKLTQ

TFCVSDFSTSDPTTEFEKFHASIRIPPFDNDLDHPGERQQISSTRERKAARILGLILGAF

ILSWLPFFIKELIVGLSIYTVSSEVADFLTWLGYVNSLINPLLYTSFNEDFKLAFKKLIR

CREHT

>h5HT1F_141

MDFLNSSDQNLTSEELLNRMPSKILVSLTLSGLALMTTTINSLVIAAIIVTRKLHHPANY

LICSLAVTDFLVAVLVMPFSIVYIVRESWIMGQVVCDIWLSVDITCCTCSILHLSAIALD

RYRAITDAVEYARKRTPKHAGIMITIVWIISVFISMPPLFWRHQGTSRDDECIIKHDHIV

STIYSTFGAFYIPLALILILYYKIYRAAKTLYHKRQASRIAKEEVNGQVLLESGEKSTKS

VSTSYVLEKSLSDPSTDFDKIHSTVRSLRSEFKHEKSWRRQKISGTRERKAATTLGLILG

AFVICWLPFFVKELVVNVCDKCKISEEMSNFLAWLGYLNSLINPLIYTIFNEDFKKAFQK

LVRCRC

>h5HT2A_125

SCLSLLHLQEKNWSALLTAVVIILTIAGNILVIMAVSLEKKLQNATNYFLMSLAIADMLL

GFLVMPVSMLTILYGYRWPLPSKLCAVWIYLDVLFSTASIMHLCAISLDRYVAIQNPIHH

SRFNSRTKAFLKIIAVWTISVGISMPIPVFGLQDDSKVFKEGSCLLADDNFVLIGSFVSF

FIPLTIMVITYFLTIKSLQKEATLCVSDLGTRAKLASFSFLPQSSLSSEKLFQRSIHREP

GSYTGRRTMQSISNEQKACKVLGIVFFLFVVMWCPFFITNIMAVICKESCNEDVIGALLN

VFVWIGYLSSAVNPLVYTLFNKTYRSAFSRYIQCQYKENKK

>h5HT2B_176

KQIVEEQGNKLHWAALLILMVIIPTIGGNTLVILAVSLEKKLQYATNYFLMSLAVADLLV

GLFVMPIALLTIMFEAMWPLPLVLCPAWLFLDVLFSTASIMHLCAISVDRYIAIKKPIQA

NQYNSRATAFIKITVVWLISIGIAIPVPIKGIETDVDNPNNITCVLTKERFGDFMLFGSL

AAFFTPLAIMIVTYFLTIHALQKKAYLVKNKPPQRLTWLTVSTVFQRDETPCSSPEKVAM

LDGSRKDKALPNSGDETLMRRTSTIGKKSVQTISNEQRASKVLGIVFFLFLLMWCPFFIT

NITLVLCDSCNQTTLQMLLEIFVWIGYVSSGVNPLVYTLFNKTFRDAFGRYITCNYRATK

S

>h5HT2C_126

GGRFKFPDGVQNWPALSIVIIIIMTIGGNILVIMAVSMEKKLHNATNYFLMSLAIADMLV

GLLVMPLSLLAILYDYVWPLPRYLCPVWISLDVLFSTASIMHLCAISLDRYVAIRNPIEH

SRFNSRTKAIMKIAIVWAISIGVSVPIPVIGLRDEEKVFVNNTTCVLNDPNFVLIGSFVA

FFIPLTIMVITYCLTIYVLRRQALMLLHGHTEEPPGLSLDFLKCCKRNTAEEENSANPNQ

DQNARRRKKKERRPRGTMQAINNERKASKVLGIVFFVFLIMWCPFFITNILSVLCEKSCN

QKLMEKLLNVFVWIGYVCSGINPLVYTLFNKIYRRAFSNYLRCNYKVEKK

>h5HT4_223

MDKLDANVSSEEGFGSVEKVVLLTFLSTVILMAILGNLLVMVAVCWDRQLRKIKTNYFIV

SLAFADLLVSVLVMPFGAIELVQDIWIYGEVFCLVRTSLDVLLTTASIFHLCCISLDRYY

AICCQPLVYRNKMTPLRIALMLGGCWVIPTFISFLPIMQGWNNIGIIDLIEKRKFNQNSN

STYCVFMVNKPYAITCSVVAFYIPFLLMVLAYYRIYVTAKEHAHQIQMLQRAGASSESRP

QSADQHSTHRMRTETKAAKTLCIIMGCFCLCWAPFFVTNIVDPFIDYTVPGQVWTAFLWL

GYINSGLNPFLYAFLNKSFRRAFLIILCCDDERYRR

>h5HT5A_194

RPSSPLLSVFGVLILTLLGFLVAATFAWNLLVLATILRVRTFHRVPHNLVASMAVSDVLV

AALVMPLSLVHELSGRRWQLGRRLCQLWIACDVLCCTASIWNVTAIALDRYWSITRHMEY

TLRTRKCVSNVMIALTWALSAVISLAPLLFGWGETYSEGSEECQVSREPSYAVFSTVGAF

YLPLCVVLFVYWKIYKAAKFRVGSRKTNSVSPISEAVEVKDSAKQPQMVFTVRHATVTFQ

PEGDTWREQKEQRAALMVGILIGVFVLCWIPFFLTELISPLCSCDIPAIWKSIFLWLGYS

NSFFNPLIYTAFNKNYNSAFKNFFSRQH

>h5HT6_209

MVPEPGPTANSTPAWGAGPPSAPGGSGWVAAALCVVIALTAAANSLLIALICTQPALRNT

SNFFLVSLFTSDLMVGLVVMPPAMLNALYGRWVLARGLCLLWTAFDVMCCSASILNLCLI

SLDRYLLILSPLRYKLRMTPLRALALVLGAWSLAALASFLPLLLGWHELGHARPPVPGQC

RLLASLPFVLVASGLTFFLPSGAICFTYCRILLAARKQAVQVASLTTGMASQASETLQVP

RTPRPGVESADSRRLATKHSRKALKASLTLGILLGMFFVTWLPFFVANIVQAVCDCISPG

LFDVLTWLGYCNSTMNPIIYPLFMRDFKRALGRFLPCPRCPRER

>h5HT7_157

GEQINYGRVEKVVIGSILTLITLLTIAGNCLVVISVCFVKKLRQPSNYLIVSLALADLSV

AVAVMPFVSVTDLIGGKWIFGHFFCNVFIAMDVMCCTASIMTLCVISIDRYLGITRPLTY

PVRQNGKCMAKMILSVWLLSASITLPPLFGWAQNVNDDKVCLISQDFGYTIYSTAVAFYI

PMSVMLFMYYQIYKAARKSAAKHKFPGFPRVEPDSVIALNGIVKLQKEVEECANLSRLLK

HERKNISIFKREQKAATTLGIIVGAFTVCWLPFFLLSTARPFICGTSCSCIPLWVERTFL

WLGYANSLINPFIYAFFNRDLRTTYRSLLQCQYRNINR

>hA1AA_162

MVFLSGNASDSSNCTQPPAPVNISKAILLGVILGGLILFGVLGNILVILSVACHRHLHSV

THYYIVNLAVADLLLTSTVLPFSAIFEVLGYWAFGRVFCNIWAAVDVLCCTASIMGLCII

SIDRYIGVSYPLRYPTIVTQRRGLMALLCVWALSLVISIGPLFGWRQPAPEDETICQINE

EPGYVLFSALGSFYLPLAIILVMYCRVYVVAKRESRGLKSGLKTDKSDSEQVTLRIHRKN

APAGGSGMASAKTKTHFSVRLLKFSREKKAAKTLGIVVGCFVLCWLPFFLVMPIGSFFPD

FKPSETVFKIVFWLGYLNSCINPIIYPCSSQEFKKAFQNVLRIQCLCRKQSSKHALGYTL

HPPSQAVEG

>hA1AB_164

LPQLDITRAISVGLVLGAFILFAIVGNILVILSVACNRHLRTPTNYFIVNLAMADLLLSF

TVLPFSAALEVLGYWVLGRIFCDIWAAVDVLCCTASILSLCAISIDRYIGVRYSLQYPTL

VTRRKAILALLSVWVLSTVISIGPLLGWKEPAPNDDKECGVTEEPFYALFSSLGSFYIPL

AVILVMYCRVYIVAKRTTKNLEAGVMKEMSNSKELTLRIHSKNFHEDTLSSTKAKGHNPR

SSIAVKLFKFSREKKAAKTLGIVVGMFILCWLPFFIALPLGSLFSTLKPPDAVFKVVFWL

GYFNSCLNPIIYPCSSKEFKRAFVRILGCQCRGRGRRRRRRRRRLGGCAYTYRPWT

>hA1AD_116

GGLVVSAQGVGVGVFLAAFILMAVAGNLLVILSVACNRHLQTVTNYFIVNLAVADLLLSA

TVLPFSATMEVLGFWAFGRAFCDVWAAVDVLCCTASILSLCTISVDRYVGVRHSLKYPAI

MTERKAAAILALLWVVALVVSVGPLLGWKEPVPPDERFCGITEEAGYAVFSSVCSFYLPM

AVIVVMYCRVYVVARSTTRSLEAGVKRERGKASEVVLRIHCRGAATGADGAHGMRSAKGH

TFRSSLSVRLLKFSREKKAAKTLAIVVGVFVLCWFPFFFVLPLGSLFPQLKPSEGVFKVI

FWLGYFNSCVNPLIYPCSSREFKRAFLRLLRCQCRRRRRRRPLWRVYGHHWRASTSGL

>hA2AA_91

MGSLQPDAGNASWNGTEAPGGGARATPYSLQVTLTLVCLAGLLMLLTVFGNVLVIIAVFT

SRALKAPQNLFLVSLASADILVATLVIPFSLANEVMGYWYFGKAWCEIYLALDVLFCTSS

IVHLCAISLDRYWSITQAIEYNLKRTPRRIKAIIITVWVISAVISFPPLISIEKKGGGGG

PQPAEPRCEINDQKWYVISSCIGSFFAPCLIMILVYVRIYQIAKRRTRVPPSRRGPDAVA

APPGGTKASRWRGRQNREKRFTFVLAVVIGVFVVCWFPFFFTYTLTAVGCSVPRTLFKFF

FWFGYCNSSLNPVIYTIFNHDFRRAFKKILCRGDRKRIV

>hA2AB_96

MDHQDPYSVQATAAIAAAITFLILFTIFGNALVILAVLTSRSLRAPQNLFLVSLAAADIL

VATLIIPFSLANELLGYWYFRRTWCEVYLALDVLFCTSSIVHLCAISLDRYWAVSRALEY

NSKRTPRRIKCIILTVWLIAAVISLPPLIYKGDQGPQPRGRPQCKLNQEAWYILASSIGS

FFAPCLIMILVYLRIYLIAKRSNRRGPRAKGGPGQGESKQPRPDRVLATLRGQVLLGRGV

GAIGGQWWRRRAQLTREKRFTFVLAVVIGVFVLCWFPFFFSYSLGAICPKHCKVPHGLFQ

FFFWIGYCNSSLNPVIYTIFNQDFRRAFRRILCRPWTQTAW

>hA2AC_97

RGQYSAGAVAGLAAVVGFLIVFTVVGNVLVVIAVLTSRALRAPQNLFLVSLASADILVAT

LVMPFSLANELMAYWYFGQVWCGVYLALDVLFCTSSIVHLCAISLDRYWSVTQAVEYNLK

RTPRRVKATIVAVWLISAVISFPPLVSLYRQPDGAAYPQCGLNDETWYILSSCIGSFFAP

CLIMGLVYARIYRVAKLRTRTLSEKRAPVGPDGASPTTENRRRRARSSVCRRKVAQAREK

RFTFVLAVVMGVFVLCWFPFFFSYSLYGICREACQVPGPLFKFFFWIGYCNSSLNPVIYT

VFNQDFRRSFKHILFRRRRRGFRQ

>hAA1_135

MPPSISAFQAAYIGIEVLIALVSVPGNVLVIWAVKVNQALRDATFCFIVSLAVADVAVGA

LVIPLAILINIGPQTYFHTCLMVACPVLILTQSSILALLAIAVDRYLRVKIPLRYKMVVT

PRRAAVAIAGCWILSFVVGLTPMFGWNNLSAVERAWAANGSMGEPVIKCEFEKVISMEYM

VYFNFFVWVLPPLLLMVLIYLEVFYLIRKQLNKKVSASSGDPQKYYGKELKIAKSLALIL

FLFALSWLPLHILNCITLFCPSCHKPSILTYIAIFLTHGNSAMNPIVYAFRIQKFRVTFL

KIWNDHFRCQPAPPIDE

>hAA2A_129

MPIMGSSVYITVELAIAVLAILGNVLVCWAVWLNSNLQNVTNYFVVSLAAADIAVGVLAI

PFAITISTGFCAACHGCLFIACFVLVLTQSSIFSLLAIAIDRYIAIRIPLRYNGLVTGTR

AKGIIAICWVLSFAIGLTPMLGWNNCGQPKEGKNHSQGCGEGQVACLFEDVVPMNYMVYF

NFFACVLVPLLLMLGVYLRIFLAARRQLKQMESQPLPGERARSTLQKEVHAAKSLAIIVG

LFALCWLPLHIINCFTFFCPDCSHAPLWLMYLAIVLSHTNSVVNPFIYAYRIREFRQTFR

KIIRSHVLRQQEPFKAA

>hAA2B_130

MLLETQDALYVALELVIAALSVAGNVLVCAAVGTANTLQTPTNYFLVSLAAADVAVGLFA

IPFAITISLGFCTDFYGCLFLACFVLVLTQSSIFSLLAVAVDRYLAICVPLRYKSLVTGT

RARGVIAVLWVLAFGIGLTPFLGWNSKDSATNNCTEPWDGTTNESCCLVKCLFENVVPMS

YMVYFNFFGCVLPPLLIMLVIYIKIFLVACRQLQRTELMDHSRTTLQREIHAAKSLAMIV

GIFALCWLPVHAVNCVTLFQPAQGKNKPKWAMNMAILLSHANSVVNPIVYAYRNRDFRYT

FHKIISRYLLCQADVKSGN

>hAA3_156

MPNNSTALSLANVTYITMEIFIGLCAIVGNVLVICVVKLNPSLQTTTFYFIVSLALADIA

VGVLVMPLAIVVSLGITIHFYSCLFMTCLLLIFTHASIMSLLAIAVDRYLRVKLTVRYKR

VTTHRRIWLALGLCWLVSFLVGLTPMFGWNMKLTSEYHRNVTFLSCQFVSVMRMDYMVYF

SFLTWIFIPLVVMCAIYLDIFYIIRNKLSLNLSNSKETGAFYGREFKTAKSLFLVLFLFA

LSWLPLSIINCIIYFNGEVPQLVLYMGILLSHANSMMNPIVYAYKIKKFKETYLLILKAC

VVCHPSDSLDT

>hAGTR1A_137

DCPKAGRHNYIFVMIPTLYSIIFVVGIFGNSLVVIVIYFYMKLKTVASVFLLNLALADLC

FLLTLPLWAVYTAMEYRWPFGNYLCKIASASVSFNLYASVFLLTCLSIDRYLAIVHPMKS

RLRRTMLVAKVTCIIIWLLAGLASLPAIIHRNVFFIENTNITVCAFHYESQNSTLPIGLG

LTKNILGFLFPFLIILTSYTLIWKALKKAYEIQKNKPRNDDIFKIIMAIVLFFFFSWIPH

QIFTFLDVLIQLGIIRDCRIADIVDTAMPITICIAYFNNCLNPLFYGFLGKKFKRYFLQL

LKYIPPKAKS

>hAGTR2_207

LNCSQKPSDKHLDAIPILYYIIFVIGFLVNIVVVTLFCCQKGPKKVSSIYIFNLAVADLL

LLATLPLWATYYSYRYDWLFGPVMCKVFGSFLTLNMFASIFFITCMSVDRYQSVIYPFLS

QRRNPWQASYIVPLVWCMACLSSLPTFYFRDVRTIEYLGVNACIMAFPPEKYAQWSAGIA

LMKNILGFIIPLIFIATCYFGIRKHLLKTNSYGKNRITRDQVLKMAAAVVLAFIICWLPF

HVLTFLDALAWMGVINSCEVIAVIDLALPFAILLGFTNSCVNPFLYCFVGNRFQQKLRSV

FRVPITWLQG

>hAVPR1A_171

PRDVRNEELAKLEIAVLAVTFAVAVLGNSSVLLALHRTPRKTSRMHLFIRHLSLADLAVA

FFQVLPQMCWDITYRFRGPDWLCRVVKHLQVFGMFASAYMLVVMTADRYIAVCHPLKTLQ

QPARRSRLMIAAAWVLSFVLSTPQYFVFSMIEVNNVTKARDCWATFIQPWGSRAYVTWMT

GGIFVAPVVILGTCYGFICYNIWCNVRGKTASRQSKGAEQAGVAFQKGFLLAPCVSSVKS

ISRAKIRTVKMTFVIVTAYIVCWAPFFIIQMWSVWDPMSVWTESENPTITITALLGSLNS

CCNPWIYMFFSGHLLQDCVQSFPCCQNMKEKF

>hB1AR_88

SPEPLSQQWTAGMGLLMALIVLLIVAGNVLVIVAIAKTPRLQTLTNLFIMSLASADLVMG

LLVVPFGATIVVWGRWEYGSFFCELWTSVDVLCVTASIETLCVIALDRYLAITSPFRYQS

LLTRARARGLVCTVWAISALVSFLPILMHWWRAESDEARRCYNDPKCCDFVTNRAYAIAS

SVVSFYVPLCIMAFVYLRVFREAQKQVKKIDSCERRFLGGPARPPSPSPSPVPAPAPPPG

PPRPAAAAATAPLANGRAGKRRPSRLVALREQKALKTLGIIMGVFTLCWLPFFLANVVKA

FHRELVPDRLFVFFNWLGYANSAFNPIIYCRSPDFRKAFQRLLCCARRAARRRHATHGDR

PRASGCLARPG

>hB2AR_84

QQRDEVWVVGMGIVMSLIVLAIVFGNVLVITAIAKFERLQTVTNYFITSLACADLVMGLA

VVPFGAAHILMKMWTFGNFWCEFWTSIDVLCVTASIETLCVIAVDRYFAITSPFKYQSLL

TKNKARVIILMVWIVSGLTSFLPIQMHWYRATHQEAINCYANETCCDFFTNQAYAIASSI

VSFYVPLVIMVFVYSRVFQEAKRQLQKIDKSEGRFHVQNLSQVEQDGRTGHGLRRSSKFC

LKEHKALKTLGIIMGTFTLCWLPFFIVNIVHVIQDNLIRKEVYILLNWIGYVNSGFNPLI

YCRSPDFRIAFQELLCLRRSSLKAYGNGYSSNGNTGEQSGYHVE

>hB2R_133

SKCPQVEWLGWLNTIQPPFLWVLFVLATLENIFVLSVFCLHKSSCTVAEIYLGNLAAADL

ILACGLPFWAITISNNFDWLFGETLCRVVNAIISMNLYSSICFLMLVSIDRYLALVKTMS

MGRMRGVRWAKLYSLVIWGCTLLLSSPMLVFRTMKEYSDEGHNVTACVISYPSLIWEVFT

NMLLNVVGFLLPLSVITFCTMQIMQVLRNNEMQKFKEIQTERRATVLVLVVLLLFIICWL

PFQISTFLDTLHRLGILSSCQDERIIDVITQIASFMAYSNSCLNPLVYVIVGKRFRKKSW

EVYQGVCQKGGCRSEPIQMENSMGTLRTSI

>hB3AR_93

VPWEAALAGALLALAVLATVGGNLLVIVAIAWTPRLQTMTNVFVTSLAAADLVMGLLVVP

PAATLALTGHWPLGATGCELWTSVDVLCVTASIETLCALAVDRYLAVTNPLRYGALVTKR

CARTAVVLVWVVSAAVSFAPIMSQWWRVGADAEAQRCHSNPRCCAFASNMPYVLLSSSVS

FYLPLLVMLFVYARVFVVATRQLRLLRGELGRFPPEESPPAPSRSLAPAPVGTCAPPEGV

PACGRRPARLLPLREHRALCTLGLIMGTFTLCWLPFFLANVLRALGGPSLVPGPAFLALN

WLGYANSAFNPLIYCRSPDFRSAFRRLLCRCGRRLPPE

>hCB1R_102

CGENFMDIECFMVLNPSQQLAIAVLSLTLGTFTVLENLLVLCVILHSRSLRCRPSYHFIG

SLAVADLLGSVIFVYSFIDFHVFHRKDSRNVFLFKLGGVTASFTASVGSLFLTAIDRYIS

IHRPLAYKRIVTRPKAVVAFCLMWTIAIVIAVLPLLGWNCEKLQSVCSDIFPHIDETYLM

FWIGVTSVLLLFIVYAYMYILWKAHSHAVRMIQRGTQKSIIIHTSEDGKVQVTRPDQARM

DIRLAKTLVLILVVLIICWGPLLAIMVYDVFGKMNKLIKTVFAFCSMLCLLNSTVNPIIY

ALRSKDLRHAFRSMFPSCEGTAQP

>hCB2R_158

MEECWVTEIANGSKDGLDSNPMKDYMILSGPQKTAVAVLCTLLGLLSALENVAVLYLILS

SHQLRRKPSYLFIGSLAGADFLASVVFACSFVNFHVFHGVDSKAVFLLKIGSVTMTFTAS

VGSLLLTAIDRYLCLRYPPSYKALLTRGRALVTLGIMWVLSALVSYLPLMGWTCCPRPCS

ELFPLIPNDYLLSWLLFIAFLFSGIIYTYGHVLWKAHQHVASLSGHQDRQVPGMARMRLD

VRLAKTLGLVLAVLLICWFPVLALMAHSLATTLSDQVKKAFAFCSMLCLINSMVNPVIYA

LRSGEIRSSAHHCLAHWKKCVRG

>hCCKAR_146

PCELGLENETLFCLDQPRPSKEWQPAVQILLYSLIFLLSVLGNTLVITVLIRNKRMRTVT

NIFLLSLAVSDLMLCLFCMPFNLIPNLLKDFIFGSAVCKTTTYFMGTSVSVSTFNLVAIS

LERYGAICKPLQSRVWQTKSHALKVIAATWCLSFTIMTPYPIYSNLVPFTKNNNQTANMC

RFLLPNDVMQQSWHTFLLLILFLIPGIVMMVAYGLISLELYQGIKFEASQKKSAKERKPS

TLMAKKRVIRMLIVIVVLFFLCWMPIFSANAWRAYDTASAERRLSGTPISFILLLSYTSS

CVNPIIYCFMNKRFRLGFMATFPCCPNPGPPGARG

>hCCKBR_147

GASLCRPGAPLLNSSSVGNLSCEPPRIRGAGTRELELAIRITLYAVIFLMSVGGNMLIIV

VLGLSRRLRTVTNAFLLSLAVSDLLLAVACMPFTLLPNLMGTFIFGTVICKAVSYLMGVS

VSVSTLSLVAIALERYSAICRPLQARVWQTRSHAARVIVATWLLSGLLMVPYPVYTVVQP

VGPRVLQCVHRWPSARVRQTWSVLLLLLLFFIPGVVMAVAYGLISRELYLGLRFDGDSDS

DSQSRVRNQGGLPGGPGSGSRPTQAKLLAKKRVVRMLLVIVVLFFLCWLPVYSANTWRAF

DGPGAHRALSGAPISFIHLLSYASACVNPLVYCFMHRRFRQACLETCARCCPRPPRARPR

A

>hCCR1_149

CQKVNERAFGAQLLPPLYSLVFVIGLVGNILVVLVLVQYKRLKNMTSIYLLNLAISDLLF

LFTLPFWIDYKLKDDWVFGDAMCKILSGFYYTGLYSEIFFIILLTIDRYLAIVHAVFALR

ARTVTFGVITSIIIWALAILASMPGLYFSKTQWEFTHHTCSLHFPHESLREWKLFQALKL

NLFGLVLPLLVMIICYTGIIKILLRRPNEKKSKAVRLIFVIMIIFFLFWTPYNLTILISV

FQDFLFTHECEQSRHLDLAVQVTEVIAYTHCCVNPVIYAFVGERFRKYLRQLFHRRVAV

>hCCR5_213

CQKINVKQIAARLLPPLYSLVFIFGFVGNMLVILILINCKRLKSMTDIYLLNLAISDLFF

LLTVPFWAHYAAAQWDFGNTMCQLLTGLYFIGFFSGIFFIILLTIDRYLAVVHAVFALKA

RTVTFGVVTSVITWVVAVFASLPGIIFTRSQKEGLHYTCSSHFPYSQYQFWKNFQTLKIV

ILGLVLPLLVMVICYSGILKTLLRCRNEKKRHRAVRLIFTIMIVYFLFWAPYNIVLLLNT

FQEFFGLNNCSSSNRLDQAMQVTETLGMTHCCINPIIYAFVGEKFRNYLLVFFQKHIAK

>hCHRM1_92

MNTSAPPAVSPNITVLAPGKGPWQVAFIGITTGLLSLATVTGNLLVLISFKVNTELKTVN

NYFLLSLACADLIIGTFSMNLYTTYLLMGHWALGTLACDLWLALDYVASNASVMNLLLIS

FDRYFSVTRPLSYRAKRTPRRAALMIGLAWLVSFVLWAPAILFWQYLVGERTVLAGQCYI

QFLSQPIITFGTAMAAFYLPVTVMCTLYWRIYRETENRARELAALQLVKEKKAARTLSAI

LLAFILTWTPYNIMVLVSTFCKDCVPETLWELGYWLCYVNSTINPMCYALCNKAFRDTFR

LLLLCRWDKRRW

>hCHRM2_86

MNNSTNSSNNSLALTSPYKTFEVVFIVLVAGSLSLVTIIGNILVMVSIKVNRHLQTVNNY

FLFSLACADLIIGVFSMNLYTLYTVIGYWPLGPVVCDLWLALDYVVSNASVMNLLIISFD

RYFCVTKPLTYPVKRTTKMAGMMIAAAWVLSFILWAPAILFWQFIVGVRTVEDGECYIQF

FSNAAVTFGTAIAAFYLPVIIMTVLYWHISRASKSRIKKDKKEPPSREKKVTRTILAILL

AFIITWAPYNVMVLINTFCAPCIPNTVWTIGYWLCYINSTINPACYALCNATFKKTFKHL

LMCHYKNIGATR

>hCHRM3_98

DPLGGHTVWQVVFIAFLTGILALVTIIGNILVIVSFKVNKQLKTVNNYFLLSLACADLII

GVISMNLFTTYIIMNRWALGNLACDLWLAIDYVASNASVMNLLVISFDRYFSITRPLTYR

AKRTTKRAGVMIGLAWVISFVLWAPAILFWQYFVGKRTVPPGECFIQFLSEPTITFGTAI

AAFYMPVTIMTILYWRIYKETEKRTKELAGLQKRKRMSLVKEKKAAQTLSAILLAFIITW

TPYNIMVLVNTFCDSCIPKTFWNLGYWLCYINSTVNPVCYALCNKTFRTTFKMLLLCQCD

KKKR

>hCHRM4_87

MANFTPVNGSSGNQSVRLVTSSSHNRYETVEMVFIATVTGSLSLVTVVGNILVMLSIKVN

RQLQTVNNYFLFSLACADLIIGAFSMNLYTVYIIKGYWPLGAVVCDLWLALDYVVSNASV

MNLLIISFDRYFCVTKPLTYPARRTTKMAGLMIAAAWVLSFVLWAPAILFWQFVVGKRTV

PDNQCFIQFLSNPAVTFGTAIAAFYLPVVIMTVLYIHISLASRSRVHKHRPEGAARERKV

TRTIFAILLAFILTWTPYNVMVLVNTFCQSCIPDTVWSIGYWLCYVNSTINPACYALCNA

TFKKTFRHLLLCQYRNIGTAR

>hCHRM5_90

MEGDSYHNATTVNGTPVNHQPLERHRLWEVITIAAVTAVVSLITIVGNVLVMISFKVNSQ

LKTVNNYYLLSLACADLIIGIFSMNLYTTYILMGRWALGSLACDLWLALDYVASNASVMN

LLVISFDRYFSITRPLTYRAKRTPKRAGIMIGLAWLISFILWAPAILCWQYLVGKRTVPL

DECQIQFLSEPTITFGTAIAAFYIPVSVMTILYCRIYRETEKRTKDLADLQKRKRVVLVK

ERKAAQTLSAILLAFIITWTPYNIMVLVSTFCDKCVPVTLWHLGYWLCYVNSTVNPICYA

LCNRTFRKTFKMLLLCRWKKKKV

>hCXCR3_204

PCPQDFSLNFDRAFLPALYSLLFLLGLLGNGAVAAVLLSRRTALSSTDTFLLHLAVADTL

LVLTLPLWAVDAAVQWVFGSGLCKVAGALFNINFYAGALLLACISFDRYLNIVHATQLYR

RGPPARVTLTCLAVWGLCLLFALPDFIFLSAHHDERLNATHCQYNFPQVGRTALRVLQLV

AGFLLPLLVMAYCYAHILAVLLVSRGQRRLRAMRLVVVVVVAFALCWTPYHLVVLVDILM

DLGALARNCGRESRVDVAKSVTSGLGYMHCCLNPLLYAFVGVKFRERMWMLLLRLGCPNQ

R

>hDRD1_103

MRTLNTSAMDGTGLVVERDFSVRILTACFLSLLILSTLLGNTLVCAAVIRFRHLRSKVTN

FFVISLAVSDLLVAVLVMPWKAVAEIAGFWPFGSFCNIWVAFDIMCSTASILNLCVISVD

RYWAISSPFRYERKMTPKAAFILISVAWTLSVLISFIPVQLSWHKAKPTSPSDGNATSLA

ETIDNCDSSLSRTYAISSSVISFYIPVAIMIVTYTRIYRIAQKQIRRIAALERAAVHAKN

CQTTTGNGKPVECSQPESSFKMSFKRETKVLKTLSVIMGVFVCCWLPFFILNCILPFCGS

GETQPFCIDSNTFDVFVWFGWANSSLNPIIYAFNADFRKAFSTLLGCYRLCPA

>hDRD2_94

SDGKADRPHYNYYATLLTLLIAVIVFGNVLVCMAVSREKALQTTTNYLIVSLAVADLLVA

TLVMPWVVYLEVVGEWKFSRIHCDIFVTLDVMMCTASILNLCAISIDRYTAVAMPMLYNT

RYSSKRRVTVMISIVWVLSFTISCPLLFGLNNADQNECIIANPAFVVYSSIVSFYVPFIV

TLLVYIKIYIVLRRRRKRVNTKRSSRAFRAHLRAPLTMSRRKLSQQKEKKATQMLAIVLG

VFIICWLPFFITHILNIHCDCNIPPVLYSAFTWLGYVNSAVNPIIYTTFNIEFRKAFLKI

LHC

>hDRD3_170

STGASQARPHAYYALSYCALILAIVFGNGLVCMAVLKERALQTTTNYLVVSLAVADLLVA

TLVMPWVVYLEVTGGVWNFSRICCDVFVTLDVMMCTASILNLCAISIDRYTAVVMPVHYQ

HGTGQSSCRRVALMITAVWVLAFAVSCPLLFGFNTTGDPTVCSISNPDFVIYSSVVSFYL

PFGVTVLVYARIYVVLKQRRRKRILTRQNSQCNSVRPGFPPLREKKATQMVAIVLGAFIV

CWLPFFLTHVLNTHCQTCHVSPELYSATTWLGYVNSALNPVIYTTFNIEFRKAFLKILSC

>hDRD4_106

GASAGLAGQGAAALVGGVLLIGAVLAGNSLVCVSVATERALQTPTNSFIVSLAAADLLLA

LLVLPLFVYSEVQGGAWLLSPRLCDALMAMDVMLCTASIFNLCAISVDRFVAVAVPLRYN

RQGGSRRQLLLIGATWLLSAAVAAPVLCGLNDVRGRDPAVCRLEDRDYVVYSSVCSFFLP

CPLMLLLYWATFRGLQRWEVARRAKLHGRAPRRPSGPGPTRRRRRAKITGRERKAMRVLP

VVVGAFLLCWTPFFVVHITQALCPACSVPPRLVSAVTWLGYVNSALNPVIYTVFNAEFRN

VFRKALRACC

>hDRD5_107

AGAPPLGPSQVVTACLLTLLIIWTLLGNVLVCAAIVRSRHLRANMTNVFIVSLAVSDLFV

ALLVMPWKAVAEVAGYWPFGAFCDVWVAFDIMCSTASILNLCVISVDRYWAISRPFRYKR

KMTQRMALVMVGLAWTLSILISFIPVQLNWHRDQAASWGGLDLPNNLANWTPWEEDFWEP

DVNAENCDSSLNRTYAISSSLISFYIPVAIMIVTYTRIYRIAQVQIRRISSLERAAEHAQ

SCRSSAACAPDTSLRASIKKETKVLKTLSVIMGVFVCCWLPFFILNCMVPFCSGHPEGPP

AGFPCVSETTFDVFVWFGWANSSLNPVIYAFNADFQKVFAQLLGCSHFCSR

>hEDG2_237

GKHLATEWNTVSKLVMGLGITVCIFIMLANLLVMVAIYVNRRFHFPIYYLMANLAAADFF

AGLAYFYLMFNTGPNTRRLTVSTWLLRQGLIDTSLTASVANLLAIAIERHITVFRMQLHT

RMSNRRVVVVIVVIWTMAIVMGAIPSVGWNCICDIENCSNMAPLYSDSYLVFWAIFNLVT

FVVMVVLYAHIFGYVRQRTMRMSRHSSGPRRNRDTMMSLLKTVVIVLGAFIICWTPGLVL

LLLDVCCPQCDVLAYEKFFLLLAEFNSAMNPIIYSYRDKEMSATFRQILCCQRSENPT

>hEDG4_53

GKELSSHWRPKDVVVVALGLTVSVLVLLTNLLVIAAIASNRRFHQPIYYLLGNLAAADLF

AGVAYLFLMFHTGPRTARLSLEGWFLRQGLLDTSLTASVATLLAIAVERHRSVMAVQLHS

RLPRGRVVMLIVGVWVAALGLGLLPAHSWHCLCALDRCSRMAPLLSRSYLAVWALSSLLV

FLLMVAVYTRIFFYVRRRVQRMAEHVSCHPRYRETTLSLVKTVVIILGAFVVCWTPGQVV

LLLDGLGCESCNVLAVEKYFLLLAEANSLVNAAVYSCRDAEMRRTFRRLLCCACLRQST

>hEDG7_35

TDTVDDWTGTKLVIVLCVGTFFCLFIFFSNSLVIAAVIKNRKFHFPFYYLLANLAAADFF

AGIAYVFLMFNTGPVSKTLTVNRWFLRQGLLDSSLTASLTNLLVIAVERHMSIMRMRVHS

NLTKKRVTLLILLVWAIAIFMGAVPTLGWNCLCNISACSSLAPIYSRSYLVFWTVSNLMA

FLIMVVVYLRIYVYVKRKTNVLSPHTSGSISRRRTPMKLMKTVMTVLGAFVVCWTPGLVV

LLLDGLNCRQCGVQHVKRWFLLLALLNSVVNPIIYSYKDEDMYGTMKKMICCFSQENPE

>hETA_117

CPQQTKITSAFKYINTVISCTIFIVGMVGNATLLRIIYQNKCMRNGPNALIASLALGDLI

YVVIDLPINVFKLLAGRWPFDHNDFGVFLCKLFPFLQKSSVGITVLNLCALSVDRYRAVA

SWSRVQGIGIPLVTAIEIVSIWILSFILAIPEAIGFVMVPFEYRGEQHKTCMLNATSKFM

EFYQDVKDWWLFGFYFCMPLVCTAIFYTLMTCEMLNRRNGSLRIALSEHLKQRREVAKTV

FCLVVIFALCWFPLHLSRILKKTVYNEMDKNRCELLSFLLLMDYIGINLATMNSCINPIA

LYFVSKKFKNCFQSCLCCCCYQSKSLMTSV

>hETBR_110

CQGPIEIKETFKYINTVVSCLVFVLGIIGNSTLLRIIYKNKCMRNGPNILIASLALGDLL

HIVIDIPINVYKLLAEDWPFGAEMCKLVPFIQKASVGITVLSLCALSIDRYRAVASWSRI

KGIGVPKWTAVEIVLIWVVSVVLAVPEAIGFDIITMDYKGSYLRICLLHPVQKTAFMQFY

KTAKDWWLFSFYFCLPLAITAFFYTLMTCEMLRKKSGMQIALNDHLKQRREVAKTVFCLV

LVFALCWLPLHLSRILKLTLYNQNDPNRCELLSFLLVLDYIGINMASLNSCINPIALYLV

SKRFKNCFKSCLCCWCQSFEEKQSLE

>hGALR1_191

MELAVGNLSEGNASCPEPPAPEPGPLFGIGVENFVTLVVFGLIFALGVLGNSLVITVLAR

SKPGKPRSTTNLFILNLSIADLAYLLFCIPFQATVYALPTWVLGAFICKFIHYFFTVSML

VSIFTLAAMSVDRYVAIVHSRRSSSLRVSRNALLGVGCIWALSIAMASPVAYHQGLFHPR

ASNQTFCWEQWPDPRHKKAYVVCTFVFGYLLPLLLICFCYAKVLNHLHKKLKNMSKKSEA

SKKKTAQTVLVVVVVFGISWLPHHIIHLWAEFGVFPLTPASFLFRITAHCLAYSNSSVNP

IIYAFLSENFRKAYKQVFKCHIRKDSHLSDT

>hGALR2_68

MNVSGCPGAGNASQAGGGGGWHPEAVIVPLLFALIFLVGTVGNTLVLAVLLRGGQAVSTT

NLFILNLGVADLCFILCCVPFQATIYTLDGWVFGSLLCKAVHFLIFLTMHASSFTLAAVS

LDRYLAIRYPLHSRELRTPRNALAAIGLIWGLSLLFSGPYLSYYRQSQLANLTVCHPAWS

APRRRAMDICTFVFSYLLPVLVLGLTYARTLRYLWRAVDPVAAGSGARRAKRKVTRMILI

VAALFCLCWMPHHALILCVWFGQFPLTRATYALRILSHLVSYANSCVNPIVYALVSKHFR

KGFRTICAGLLGRAPG

>hGALR3_72

MADAQNISLDSPGSVGAVAVPVVFALIFLLGTVGNGLVLAVLLQPGPSAWQEPGSTTDLF

ILNLAVADLCFILCCVPFQATIYTLDAWLFGALVCKAVHLLIYLTMYASSFTLAAVSVDR

YLAVRHPLRSRALRTPRNARAAVGLVWLLAALFSAPYLSYYGTVRYGALELCVPAWEDAR

RRALDVATFAAGYLLPVAVVSLAYGRTLRFLWAAVGPAGAAAAEARRRATGRAGRAMLAV

AALYALCWGPHHALILCFWYGRFAFSPATYACRLASHCLAYANSCLNPLVYALASRHFRA

RFRRLWPCGRRRRHR

>hGHSR_238

LQLFPAPLLAGVTATCVALFVVGIAGNLLTMLVVSRFRELRTTTNLYLSSMAFSDLLIFL

CMPLDLVRLWQYRPWNFGDLLCKLFQFVSESCTYATVLTITALSVERYFAICFPLRAKVV

VTKGRVKLVIFVIWAVAFCSAGPIFVLVGVEHENGTDPWDTNECRPTEFAVRSGLLTVMV

WVSSIFFFLPVFCLTVLYSLIGRKLWRRRRGDAVVGASLRDQNHKQTVKMLAVVVFAFIL

CWLPFHVGRYLFSKSFEPGSLEIAQISQYCNLVSFVLFYLSAAINPILYNIMSKKYRVAV

FRLLGFEPFSQR

>hGNRHR_142

NHCSAINNSIPLMQGNLPTLTLSGKIRVTVTFFLFLLSATFNASFLLKLQKWTQKKEKGK

KLSRMKLLLKHLTLANLLETLIVMPLDGMWNITVQWYAGELLCKVLSYLKLFSMYAPAFM

MVVISLDRSLAITRPLALKSNSKVGQSMVGLAWILSSVFAGPQLYIFRMIHLADSSGQTK

VFSQCVTHCSFSQWWHQAFYNFFTFSCLFIIPLFIMLICNAKIIFTLTRVLHQDPHELQL

NQSKNNIPRARLKTLKMTVAFATSFTVCWTPYYVLGIWYWFDPEMLNRLSDPVNHFFFLF

AFLNPCFDPLIYGYFSL

>hGPR14_283

PSSLEDLVATGTIGTLLSAMGVVGVVGNAYTLVVTCRSLRAVASMYVYVVNLALADLLYL

LSIPFIVATYVTKEWHFGDVGCRVLFGLDFLTMHASIFTLTVMSSERYAAVLRPLDTVQR

PKGYRKLLALGTWLLALLLTLPVMLAMRLVRRGPKSLCLPAWGPRAHRAYLTLLFATSIA

GPGLLIGLLYARLARAYRRSQRASFKRARRPGARALRLVLGIVLLFWACFLPFWLWQLLA

QYHQAPLAPRTARIVNYLTTCLTYGNSCANPFLYTLLTRNYRDHLRGRVRGPGSGGGR

>hGPR24_245

ISYINIIMPSVFGTICLLGIIGNSTVIFAVVKKSKLHWCNNVPDIFIINLSVVDLLFLLG

MPFMIHQLMGNGVWHFGETMCTLITAMDANSQFTSTYILTAMAIDRYLATVHPISSTKFR

KPSVATLVICLLWALSFISITPVWLYARLIPFPGGAVGCGIRLPNPDTDLYWFTLYQFFL

AFALPFVVITAAYVRILQRMTSSVAPASQRSIRLRTKRVTRTAIAICLVFFVCWAPYYVL

QLTQLSISRPTLTFVYLYNAAISLGYANSCLNPFVYIVLCETFRKRLVLSVKPAAQGQLR

AVSNAQTADEERTESKGT

>hGPR44_293

TSIRYIDHAAVLLHGLASLLGLVENGVILFVVGCRMRQTVVTTWVLHLALSDLLASASLP

FFTYFLAVGHSWELGTTFCKLHSSIFFLNMFASGFLLSAISLDRCLQVVRPVWAQNHRTV

AAAHKVCLVLWALAVLNTVPYFVFRDTISRLDGRIMCYYNVLLLNPGPDRDATCNSRQAA

LAVSKFLLAFLVPLAIIASSHAAVSLRLQHRGRRRPGRFVRLVAAVVAAFALCWGPYHVF

SLLEARAHANPGLRPLVWRGLPFVTSLAFFNSVANPVLYVLTCPDMLRKLRRSLRTVLES

VLVDDSELGGAGS

>hGPR92_260

PDYRPTHRLHLVVYSLVLAAGLPLNALALWVFLRALRVHSVVSVYMCNLAASDLLFTLSL

PVRLSYYALHHWPFPDLLCQTTGAIFQMNMYGSCIFLMLINVDRYAAIVHPLRLRHLRRP

RVARLLCLGVWALILVFAVPAARVHRPSRCRYRDLEVRLCFESFSDELWKGRLLPLVLLA

EALGFLLPLAAVVYSSGRVFWTLARPDATQSQRRRKTVRLLLANLVIFLLCFVPYNSTLA

VYGLLRSKLVAASVPARDRVRGVLMVMVLLAGANCVLDPLVYYFSAEGFRNTLRGLGTPH

RARTSATNGTRAALAQ

>hHRH1_163

KTTMASPQLMPLVVVLSTICLVTVGLNLLVLYAVRSERKLHTVGNLYIVSLSVADLIVGA

VVMPMNILYLLMSKWSLGRPLCLFWLSMDYVASTASIFSVFILCIDRYRSVQQPLRYLKY

RTKTRASATILGAWFLSFLWVIPILGWNHFMQQTSVRREDKCETDFYDVTWFKVMTAIIN

FYLPTLLMLWFYAKIYKAVRQHCQHRELINRSLPSFSRQYVSGLHMNRERKAAKQLGFIM

AAFILCWIPYFIFFMVIAFCKNCCNEHLHMFTIWLGYINSTLNPLIYPLCNENFKKTFKR

ILHIRS

>hHRH2_111

CLDSTACKITITVVLAVLILITVAGNVVVCLAVGLNRRLRNLTNCFIVSLAITDLLLGLL

VLPFSAIYQLSCKWSFGKVFCNIYTSLDVMLCTASILNLFMISLDRYCAVMDPLRYPVLV

TPVRVAISLVLIWVISITLSFLSIHLGWNSRNETSKGNHTTSKCKVQVNEVYGLVDGLVT

FYLPLLIMCITYYRIFKVARDQAKRINHISSWKAATIREHKATVTLAAVMGAFIICWFPY

FTAFVYRGLRGDDAINEVLEAIVLWLGYANSALNPILYAALNRDFRTGYQQLFCCRLANR

NSH

>hHRH3_290

ARGFSAAWTAVLAALMALLIVATVLGNALVMLAFVADSSLRTQNNFFLLNLAISDFLVGA

FCIPLYVPYVLTGRWTFGRGLCKLWLVVDYLLCTSSAFNIVLISYDRFLSVTRAVSYRAQ

QGDTRRAVRKMLLVWVLAFLLYGPAILSWEYLSGGSSIPEGHCYAEFFYNWYFLITASTL

EFFTPFLSVTFFNLSIYLNIQRRTRLRLDGAREAAGLSRDRKVAKSLAVIVSIFGLCWAP

YTLLMIIRAACHGHCVPDYWYETSFWLLWANSAVNPVLYPLCHHSFRRAFTKLLCPQKLK

IQPH

>hHRH4_264

MPDTNSTINLSLSTRVTLAFFMSLVAFAIMLGNALVILAFVVDKNLRHRSSYFFLNLAIS

DFFVGVISIPLYIPHTLFEWDFGKEICVFWLTTDYLLCTASVYNIVLISYDRYLSVSNAV

SYRTQHTGVLKIVTLMVAVWVLAFLVNGPMILVSESWKDEGSECEPGFFSEWYILAITSF

LEFVIPVILVAYFNMNIYWSLWKRDHLSRCQSHPGLTLLRARRLAKSLAILLGVFAVCWA

PYSLFTIVLSFYSSATGPKSVWYRIAFWLQWFNSFVNPLLYPLCHKRFQKAFLKIFCIKK

QPLPSQ

>hMC1R_219

QTGARCLEVSISDGLFLSLGLVSLVENALVVATIAKNRNLHSPMYCFICCLALSDLLVSG

SNVLETAVILLLEAGALVARAAVLQQLDNVIDVITCSSMLSSLCFLGAIAVDRYISIFYA

LRYHSIVTLPRARRAVAAIWVASVVFSTLFIAYYDHVAVLLCLVVFFLAMLVLMAVLYVH

MLARACQHAQGIARLHKRQRPVHQGFGLKGAVTLTILLGIFFLCWGPFFLHLTLIVLCPE

HPTCGCIFKNFNLFLALIICNAIIDPLIYAFHSQELRRTLKEVLTCSW

>hMC3R_178

SSSAFCEQVFIKPEVFLSLGIVSLLENILVILAVVRNGNLHSPMYFFLCSLAVADMLVSV

SNALETIMIAIVHSDYLTFEDQFIQHMDNIFDSMICISLVASICNLLAIAVDRYVTIFYA

LRYHSIMTVRKALTLIVAIWVCCGVCGVVFIVYSESKMVIVCLITMFFAMMLLMGTLYVH

MFLFARLHVKRIAALPPADGVAPQQHSCMKGAVTITILLGVFIFCWAPFFLHLVLIITCP

TNPYCICYTAHFNTYLVLIMCNSVIDPLIYAFRSLELRNTFREILCGCNGMNLG

>hMC4R_148

SDGGCYEQLFVSPEVFVTLGVISLLENILVIVAIAKNKNLHSPMYFFICSLAVADMLVSV

SNGSETIVITLLNSTDTDAQSFTVNIDNVIDSVICSSLLASICSLLSIAVDRYFTIFYAL

QYHNIMTVKRVGIIISCIWAACTVSGILFIIYSDSSAVIICLITMFFTMLALMASLYVHM

FLMARLHIKRIAVLPGTGAIRQGANMKGAITLTILIGVFVVCWAPFFLHLIFYISCPQNP

YCVCFMSHFNLYLILIMCNSIIDPLIYALRSQELRKTFKEIICCYPLGGLCDLSSRY

>hMC5R_155

NKSSPCEDMGIAVEVFLTLGVISLLENILVIGAIVKNKNLHSPMYFFVCSLAVADMLVSM

SSAWETITIYLLNNKHLVIADAFVRHIDNVFDSMICISVVASMCSLLAIAVDRYVTIFYA

LRYHHIMTARRSGAIIAGIWAFCTGCGIVFILYSESTYVILCLISMFFAMLFLLVSLYIH

MFLLARTHVKRIAALPGASSARQRTSMQGAVTVTMLLGVFTVCWAPFFLHLTLMLSCPQN

LYCSRFMSHFNMYLILIMCNSVMDPLIYAFRSQEMRKTFKEIICCRGFRIACSFPRRD

>hMTNR1A_197

MQGNGSALPNASQPVLRGDGARPSWLASALACVLIFTIVVDILGNLLVILSVYRNKKLRN

AGNIFVVSLAVADLVVAIYPYPLVLMSIFNNGWNLGYLHCQVSGFLMGLSVIGSIFNITG

IAINRYCYICHSLKYDKLYSSKNSLCYVLLIWLLTLAAVLPNLRAGTLQYDPRIYSCTFA

QSVSSAYTIAVVVFHFLVPMIIVIFCYLRIWILVLQVRQRVKPDRKPKLKPQDFRNFVTM

FVVFVLFAICWAPLNFIGLAVASDPASMVPRIPEWLFVASYYMAYFNSCLNAIIYGLLNQ

NFRKEYRRIIVSLCTARVFFVDSSNDVADRVKWKPSP

>hMTNR1B_203

NGSFANCCEAGGWAVRPGWSGAGSARPSRTPRPPWVAPALSAVLIVTTAVDVVGNLLVIL

SVLRNRKLRNAGNLFLVSLALADLVVAFYPYPLILVAIFYDGWALGEEHCKASAFVMGLS

VIGSVFNITAIAINRYCYICHSMAYHRIYRRWHTPLHICLIWLLTVVALLPNFFVGSLEY

DPRIYSCTFIQTASTQYTAAVVVIHFLLPIAVVSFCYLRIWVLVLQARRKAKPESRLCLK

PSDLRSFLTMFVVFVIFAICWAPLNCIGLAVAINPQEMAPQIPEGLFVTSYLLAYFNSCL

NAIVYGLLNQNFRREYKRILLALWNPRHCIQDASKGSHAEGLQSPAP

>hNK1R_118

MDNVLPVDSDLSPNISTNTSEPNQFVQPAWQIVLWAAAYTVIVVTSVVGNVVVMWIILAH

KRMRTVTNYFLVNLAFAEASMAAFNTVVNFTYAVHNEWYYGLFYCKFHNFFPIAAVFASI

YSMTAVAFDRYMAIIHPLQPRLSATATKVVICVIWVLALLLAFPQGYYSTTETMPSRVVC

MIEWPEHPNKIYEKVYHICVTVLIYFLPLLVIGYAYTVVGITLWASEIPGDSSDRYHEQV

SAKRKVVKMMIVVVCTFAICWLPFHIFFLLPYINPDLYLKKFIQQVYLAIMWLAMSSTMY

NPIIYCCLNDRFRLGFKHAFRCCPFISAGD

>hNK2R_99

MGTCDIVTEANISSGPESNTTGITAFSMPSWQLALWATAYLALVLVAVTGNAIVIWIILA

HRRMRTVTNYFIVNLALADLCMAAFNAAFNFVYASHNIWYFGRAFCYFQNLFPITAMFVS

IYSMTAIAADRYMAIVHPFQPRLSAPSTKAVIAGIWLVALALASPQCFYSTVTMDQGATK

CVVAWPEDSGGKTLLLYHLVVIALIYFLPLAVMFVAYSVIGLTLWRRAVPGHQAHGANLR

HLQAKKKFVKTMVLVVLTFAICWLPYHLYFILGSFQEDIYCHKFIQQVYLALFWLAMSST

MYNPIIYCCLNHRFRSGFRLAFRCCPWVTPTK

>hNK3R_131

ANLTNQFVQPSWRIALWSLAYGVVVAVAVLGNLIVIWIILAHKRMRTVTNYFLVNLAFSD

ASMAAFNTLVNFIYALHSEWYFGANYCRFQNFFPITAVFASIYSMTAIAVDRYMAIIDPL

KPRLSATATKIVIGSIWILAFLLAFPQCLYSKTKVMPGRTLCFVQWPEGPKQHFTYHIIV

IILVYCFPLLIMGITYTIVGITLWGGEIPGDTCDKYHEQLKAKRKVVKMMIIVVMTFAIC

WLPYHIYFILTAIYQQLNRWKYIQQVYLASFWLAMSSTMYNPIIYCCLNKRFRAGFKRAF

RWCPFIKVSS

>hNPY1R_122

NDDCHLPLAMIFTLALAYGAVIILGVSGNLALIIIILKQKEMRNVTNILIVNLSFSDLLV

AIMCLPFTFVYTLMDHWVFGEAMCKLNPFVQCVSITVSIFSLVLIAVERHQLIINPRGWR

PNNRHAYVGIAVIWVLAVASSLPFLIYQVMTDEPFQNVTLDAYKDKYVCFDQFPSDSHRL

SYTTLLLVLQYFGPLCFIFICYFKIYIRLKRRNNMMDKMRDNKYRSSETKRINIMLLSIV

VAFAVCWLPLTIFNTVFDWNHQIIATCNHNLLFLLCHLTAMISTCVNPIFYGFLNKNFQR

DLQFFFNFCDFRSRDDDYETIAMSTMHTDVSKT

>hNPY2R_201

LIDSTKLIEVQVVLILAYCSIILLGVIGNSLVIHVVIKFKSMRTVTNFFIANLAVADLLV

NTLCLPFTLTYTLMGEWKMGPVLCHLVPYAQGLAVQVSTITLTVIALDRHRCIVYHLESK

ISKRISFLIIGLAWGISALLASPLAIFREYSLIEIIPDFEIVACTEKWPGEEKSIYGTVY

SLSSLLILYVLPLGIISFSYTRIWSKLKNHVSPGAANDHYHQRRQKTTKMLVCVVVVFAV

SWLPLHAFQLAVDIDSQVLDLKEYKLIFTVFHIIAMCSTFANPLLYGWMNSNYRKAFLSA

FRCEQRLDAIHSEVSVTFKAKKNLEVRK

>hNPY5R_231

DDYKSSVDDLQYFLIGLYTFVSLLGFMGNLLILMALMKKRNQKTTVNFLIGNLAFSDILV

VLFCSPFTLTSVLLDQWMFGKVMCHIMPFLQCVSVLVSTLILISIAIVRYHMIKHPISNN

LTANHGYFLIATVWTLGFAICSPLPVFHSLVELQETFGSALLSSRYLCVESWPSDSYRIA

FTISLLLVQYILPLVCLTVSHTSVCRSISCGLSNELRVKRSVTRIKKRSRSVFYRLTILI

LVFAVSWMPLHLFHVVTDFNDNLISNRHFKLVYCICHLLGMMSCCLNPILYGFLNNGIKA

DLVSLIHCLHM

>hNTSR1_143

LDVNTDIYSKVLVTAVYLALFVVGTVGNTVTAFTLARKKSLQSLQSTVHYHLGSLALSDL

LTLLLAMPVELYNFIWVHHPWAFGDAGCRGYYFLRDACTYATALNVASLSVERYLAICHP

FKAKTLMSRSRTKKFISAIWLASALLAVPMLFTMGEQNRSADGQHAGGLVCTPTIHTATV

KVVIQVNTFMSFIFPMVVISVLNTIIANKLTVMVRQAAEQGQVCTVGGEHSTFSMAIEPG

RVQALRHGVRVLRAVVIAFVVCWLPYHVRRLMFCYISDEQWTPFLYDFYHYFYMVTNALF

YVSSTINPILYNLVSANFRHIFLATLACLCPVWRR

>hOPRD1_172

ASSLALAIAITALYSAVCAVGLLGNVLVMFGIVRYTKMKTATNIYIFNLALADALATSTL

PFQSAKYLMETWPFGELLCKAVLSIDYYNMFTSIFTLTMMSVDRYIAVCHPVKALDFRTP

AKAKLINICIWVLASGVGVPIMVMAVTRPRDGAVVCMLQFPSPSWYWDTVTKICVFLFAF

VVPILIITVCYGLMLLRLRSVRLLSGSKEKDRSLRRITRMVLVVVGAFVVCWAPIHIFVI

VWTLVDIDRRDPLVVAALHLCIALGYANSSLNPVLYAFLDENFKRCFRQLCRKPCGRPDP

>hOPRK1_173

PAHISPAIPVIITAVYSVVFVVGLVGNSLVMFVIIRYTKMKTATNIYIFNLALADALVTT

TMPFQSTVYLMNSWPFGDVLCKIVISIDYYNMFTSIFTLTMMSVDRYIAVCHPVKALDFR

TPLKAKIINICIWLLSSSVGISAIVLGGTKVREDVDVIECSLQFPDDDYSWWDLFMKICV

FIFAFVIPVLIIIVCYTLMILRLKSVRLLSGSREKDRNLRRITRLVLVVVAVFVVCWTPI

HIFILVEALGSTSHSTAALSSYYFCIALGYTNSSLNPILYAFLDENFKRCFRDFCFPLKM

RMER

>hOPRL1_174

GAFLPLGLKVTIVGLYLAVCVGGLLGNCLVMYVILRHTKMKTATNIYIFNLALADTLVLL

TLPFQGTDILLGFWPFGNALCKTVIAIDYYNMFTSTFTLTAMSVDRYVAICHPIRALDVR

TSSKAQAVNVAIWALASVVGVPVAIMGSAQVEDEEIECLVEIPTPQDYWGPVFAICIFLF

SFIVPVLVISVCYSLMIRRLRGVRLLSGSREKDRNLRRITRLVLVVVAVFVGCWTPVQVF

VLAQGLGVQPSSETAVAILRFCTALGYVNSCLNPILYAFLDENFKACFRKFCCASALRRD

V

>hOPRM1_166

SPSMITAITIMALYSIVCVVGLFGNFLVMYVIVRYTKMKTATNIYIFNLALADALATSTL

PFQSVNYLMGTWPFGTILCKIVISIDYYNMFTSIFTLCTMSVDRYIAVCHPVKALDFRTP

RNAKIINVCNWILSSAIGLPVMFMATTKYRQGSIDCTLTFSHPTWYWENLLKICVFIFAF

IMPVLIITVCYGLMILRLKSVRMLSGSKEKDRNLRRITRMVLVVVAVFIVCWTPIHIYVI

IKALVTIPETTFQTVSWHFCIALGYTNSCLNPVLYAFLDENFKRCFREFCIPTSSNIEQ

>hOXTR_138

GPPRRNEALARVEVAVLCLILLLALSGNACVLLALRTTRQKHSRLFFFMKHLSIADLVVA

VFQVLPQLLWDITFRFYGPDLLCRLVKYLQVVGMFASTYLLLLMSLDRCLAICQPLRSLR

RRTDRLAVLATWLGCLVASAPQVHIFSLREVADGVFDCWAVFIQPWGPKAYITWITLAVY

IVPVIVLAACYGLISFKIWQNLRLKTAAAAAAEAPEGAAAGDGGRVALARVSSVKLISKA

KIRTVKMTFIIVLAFIVCWTPFFFVQMWSVWDANAPKEASAFIIVMLLASLNSCCNPWIY

MLFTGHLFHELVQRFLCCSASYLKG

>hPAFR_119

MEPHDSSHMDSEFRYTLFPIVYSIIFVLGVIANGYVLWVFARLYPCKKFNEIKIFMVNLT

MADMLFLITLPLWIVYYQNQGNWILPKFLCNVAGCLFFINTYCSVAFLGVITYNRFQAVT

RPIKTAQANTRKRGISLSLVIWVAIVGAASYFLILDSTNTVPDSAGSGNVTRCFEHYEKG

SVPVLIIHIFIVFSFFLVFLIILFCNLVIIRTLLMQPVQQQRNAEVKRRALWMVCTVLAV

FIICFVPHHVVQLPWTLAELGFQDSKFHQAINDAHQVTLCLLSTNCVLDPVIYCFLTKKF

RKHLTEKFYSMRSSRKCS

>hPTGDR_220

MKSPFYRCQNTTSVEKGNSAVMGGVLFSTGLLGNLLALGLLARSGLGWCSRRPLRPLPSV

FYMLVCGLTVTDLLGKCLLSPVVLAAYAQNRSLRVLAPALDNSLCQAFAFFMSFFGLSST

LQLLAMALECWLSLGHPFFYRRHITLRLGALVAPVVSAFSLAFCALPFMGFGKFVQYCPG

TWCFIQMVHEEGSLSVLGYSVLYSSLMALLVLATVLCNLGAMRNLYAMHRRLQRHPRSCT

RDCAEPRADGREASPQPLEELDHLLLLALMTVLFTMCSLPVIYRAYYGAFKDVKEKNRTS

EEAEDLRALRFLSVISIVDPWIFIIFRSPVFRIFFHKIFIRPLR

>hPTGER1_160

MSPCGPLNLSLAGEATTCAAPWVPNTSAVPPSGASPALPIFSMTLGAVSNLLALALLAQA

AGRLRRRRSAATFLLFVASLLATDLAGHVIPGALVLRLYTAGRAPAGGACHFLGGCMVFF

GLCPLLLGCGMAVERCVGVTRPLLHAARVSVARARLALAAVAAVALAVALLPLARVGRYE

LQYPGTWCFIGLGPPGGWRQALLAGLFASLGLVALLAALVCNTLSGLALLRARWRRRSRR

PPPASGPDSRRRWGAHGPRSASASSASSIASASTFFGGSRSSGSARRARAHDVEMVGQLV

GIMVVSCICWSPMLVLVALAVGGWSSTSLQRPLFLAVRLASWNQILDPWVYILLRQAVLR

QLLRLLPPRAGA

>hPTGER2_181

MGNASNDSQSEDCETRQWLPPGESPAISSVMFSAGVLGNLIALALLARRWRGDVGCSAGR

RSSLSLFHVLVTELVFTDLLGTCLISPVVLASYARNQTLVALAPESRACTYFAFAMTFFS

LATMLMLFAMALERYLSIGHPYFYQRRVSRSGGLAVLPVIYAVSLLFCSLPLLDYGQYVQ

YCPGTWCFIRHGRTAYLQLYATLLLLLIVSVLACNFSVILNLIRMHRRSRRSRCGPSLGS

GRGGPGARRRGERVSMAEETDHLILLAIMTITFAVCSLPFTIFAYMNETSSRKEKWDLQA

LRFLSINSIIDPWVFAILRPPVLRLMRSVLCCRISL

>hPTGER3_180

LTRPPGSGEDCGSVSVAFPITMLLTGFVGNALAMLLVSRSYRRRESKRKKSFLLCIGWLA

LTDLVGQLLTTPVVIVVYLSKQRWEHIDPSGRLCTFFGLTMTVFGLSSLFIASAMAVERA

LAIRAPHWYASHMKTRATRAVLLGVWLAVLAFALLPVLGVGQYTVQWPGTWCFISTGRGG

NGTSSSHNWGNLFFASAFAFLGLLALTVTFSCNLATIKALVSRCRAKATASQSSAQWGRI

TTETAIQLMGIMCVLSVCWSPLLIMMLKMIFNQTSVEHCKTHTEKQKECNFFLIAVRLAS

LNQILDPWVYLLLRKILLRKFCQIRYHTNNY

>hPTGER4_167

MSTPGVNSSASLSPDRLNSPVTIPAVMFIFGVVGNLVAIVVLCKSRKEQKETTFYTLVCG

LAVTDLLGTLLVSPVTIATYMKGQWPGGQPLCEYSTFILLFFSLSGLSIICAMSVERYLA

INHAYFYSHYVDKRLAGLTLFAVYASNVLFCALPNMGLGSSRLQYPDTWCFIDWTTNVTA

HAAYSYMYAGFSSFLILATVLCNVLVCGALLRMHRQFMRRTSLGTEQHHAAAAASVASRG

HPAASPALPRLSDFRRRRSFRRIAGAEIQMVILLIATSLVVLICSIPLVVRVFVNQLYQP

SLEREVSKNPDLQAIRIASVNPILDPWIYILLRKTVLSKAIEKIKCLFCR

>hPTGFR_179

MSMNNSKQLVSPAAALLSNTTCQTENRLSVFFSVIFMTVGILSNSLAIAILMKAYQRFRQ

KSKASFLLLASGLVITDFFGHLINGAIAVFVYASDKEWIRFDQSNVLCSIFGICMVFSGL

CPLLLGSVMAIERCIGVTKPIFHSTKITSKHVKMMLSGVCLFAVFIALLPILGHRDYKIQ

ASRTWCFYNTEDIKDWEDRFYLLLFSFLGLLALGVSLLCNAITGITLLRVKFKSQQHRQG

RSHHLEMVIQLLAIMCVSCICWSPFLVTMANIGINGNHSLETCETTLFALRMATWNQILD

PWVYILLRKAVLKNLYKLASQCCGVHV

>hPTGIR_182

MADSCRNLTYVRGSVGPATSTLMFVAGVVGNGLALGILSARRPARPSAFAVLVTGLAATD

LLGTSFLSPAVFVAYARNSSLLGLARGGPALCDAFAFAMTFFGLASMLILFAMAVERCLA

LSHPYLYAQLDGPRCARLALPAIYAFCVLFCALPLLGLGQHQQYCPGSWCFLRMRWAQPG

GAAFSLAYAGLVALLVAAIFLCNGSVTLSLCRMYRQQKRHQGSLGPRPRTGEDEVDHLIL

LALMTVVMAVCSLPLTIRCFTQAVAPDSSSEMGDLLAFRFYAFNPILDPWVFILFRKAVF

QRLKLWVCCLCLG

>hSSTR1_139

QNGTLSEGQGSAILISFIYSVVCLVGLCGNSMVIYVILRYAKMKTATNIYILNLAIADEL

LMLSVPFLVTSTLLRHWPFGALLCRLVLSVDAVNMFTSIYCLTVLSVDRYVAVVHPIKAA

RYRRPTVAKVVNLGVWVLSLLVILPIVVFSRTAANSDGTVACNMLMPEPAQRWLVGFVLY

TFLMGFLLPVGAICLCYVLIIAKMRMVALKAGWQQRKRSERKITLMVMMVVMVFVICWMP

FYVVQLVNVFAEQDDATVSQLSVILGYANSCANPILYGFLSDNFKRSFQRILCLSWMDNA

AEEP

>hSSTR2_140

NQTEPYYDLTSNAVLTFIYFVVCIIGLCGNTLVIYVILRYAKMKTITNIYILNLAIADEL

FMLGLPFLAMQVALVHWPFGKAICRVVMTVDGINQFTSIFCLTVMSIDRYLAVVHPIKSA

KWRRPRTAKMITMAVWGVSLLVILPIMIYAGLRSNQWGRSSCTINWPGESGAWYTGFIIY

TFILGFLVPLTIICLCYLFIIIKVKSSGIRVGSSKRKKSEKKVTRMVSIVVAVFIFCWLP

FYIFNVSSVSMAISPTPALKGMFDFVVVLTYANSCANPILYAFLSDNFKKSFQNVLCLVK

VSGTDDGE

>hSSTR3_154

AGPSPAGLAVSGVLIPLVYLVVCVVGLLGNSLVIYVVLRHTASPSVTNVYILNLALADEL

FMLGLPFLAAQNALSYWPFGSLMCRLVMAVDGINQFTSIFCLTVMSVDRYLAVVHPTRSA

RWRTAPVARTVSAAVWVASAVVVLPVVVFSGVPRGMSTCHMQWPEPAAAWRAGFIIYTAA

LGFFGPLLVICLCYLLIVVKVRSAGRRVWAPSCQRRRRSERRVTRMVVAVVALFVLCWMP

FYVLNIVNVVCPLPEEPAFFGLYFLVVALPYANSCANPILYGFLSYRFKQGFRRVLLRPS

RRVRSQEPTVGPPEKTEEEDEEE

>hSSTR4_145

VAGPGDARAAGMVAIQCIYALVCLVGLVGNALVIFVILRYAKMKTATNIYLLNLAVADEL

FMLSVPFVASSAALRHWPFGSVLCRAVLSVDGLNMFTSVFCLTVLSVDRYVAVVHPLRAA

TYRRPSVAKLINLGVWLASLLVTLPIAIFADTRPARGGQAVACNLQWPHPAWSAVFVVYT

FLLGFLLPVLAIGLCYLLIVGKMRAVALRAGWQQRRRSEKKITRLVLMVVVVFVLCWMPF

YVVQLLNLVVTSLDATVNHVSLILSYANSCANPILYGFLSDNFRRSFQRVLCLRCCLLEG

AGG

>hSSTR5_161

LVGPAPSAGARAVLVPVLYLLVCAAGLGGNTLVIYVVLRFAKMKTVTNIYILNLAVADVL

YMLGLPFLATQNAASFWPFGPVLCRLVMTLDGVNQFTSVFCLTVMSVDRYLAVVHPLSSA

RWRRPRVAKLASAAAWVLSLCMSLPLLVFADVQEGGTCNASWPEPVGLWGAVFIIYTAVL

GFFAPLLVICLCYLLIVVKVRAAGVRVGCVRRRSERKVTRMVLVVVLVFAGCWLPFFTVN

IVNLAVALPQEPASAGLYFFVVILSYANSCANPVLYGFLSDNFRQSFQKVLCLRKGSGAK

DAD

>hTBXA2R_105

MWPNGSSLGPCFRPTNITLEERRLIASPWFAASFCVVGLASNLLALSVLAGARQGGSHTR

SSFLTFLCGLVLTDFLGLLVTGTIVVSQHAALFEWHAVDPGCRLCRFMGVVMIFFGLSPL

LLGAAMASERYLGITRPFSRPAVASQRRAWATVGLVWAAALALGLLPLLGVGRYTVQYPG

SWCFLTLGAESGDVAFGLLFSMLGGLSVGLSFLLNTVSVATLCHVYHGQEAAQQRPRDSE

VEMMAQLLGIMVVASVCWLPLLVFIAQTVLRNPPAMSPAGQLSRTTEKELLIYLRVATWN

QILDPWVYILFRRAVLRRLQPRLSTRPRR

>hV2R_134

PLDTRDPLLARAELALLSIVFVAVALSNGLVLAALARRGRRGHWAPIHVFIGHLCLADLA

VALFQVLPQLAWKATDRFRGPDALCRAVKYLQMVGMYASSYMILAMTLDRHRAICRPMLA

YRHGSGAHWNRPVLVAWAFSLLLSLPQLFIFAQRNVEGGSGVTDCWACFAEPWGRRTYVT

WIALMVFVAPTLGIAACQVLIFREIHASLVPGPSERPGGRRRGRRTGSPGEGAHVSAAVA

KTVRMTLVIVVVYVLCWAPFFLVQLWAAWDPEAPLEGAPFVLLMLLASLNSCTNPWIYAS

FSSSVSSELRSLLCCARGRTPP

**Kinase**

>hABL1_1950

ERTDITMKHKLGGGQYGEVYEGVWKKYSLTVAVKTLKEDTMEVEEFLKEAAVMKEIKHPN

LVQLLGVCTREPPFYIITEFMTYGNLLDYLRECNRQEVNAVVLLYMATQISSAMEYLEKK

NFIHRDLAARNCLVGENHLVKVADFGLSRLMTGDTYTAHAGAKFPIKWTAPESLAYNKFS

IKSDVWAFGVLLWEIATYGMSPYPGIDLSQVYELLEKDYRMERPEGCPEKVYELMRACWQ

WNPSDRPSFAEIHQAFETM

>hABL2_1942

ERTDITMKHKLGGGQYGEVYVGVWKKYSLTVAVKTLKEDTMEVEEFLKEAAVMKEIKHPN

LVQLLGVCTLEPPFYIVTEYMPYGNLLDYLRECNREEVTAVVLLYMATQISSAMEYLEKK

NFIHRDLAARNCLVGENHVVKVADFGLSRLMTGDTYTAHAGAKFPIKWTAPESLAYNTFS

IKSDVWAFGVLLWEIATYGMSPYPGIDLSQVYDLLEKGYRMEQPEGCPPKVYELMRACWK

WSPADRPSFAETHQAFETM

>hAKT1_2014

TMNEFEYLKLLGKGTFGKVILVKEKATGRYYAMKILKKEVIVAKDEVAHTLTENRVLQNS

RHPFLTALKYSFQTHDRLCFVMEYANGGELFFHLSRERVFSEDRARFYGAEIVSALDYLH

SEKNVVYRDLKLENLMLDKDGHIKITDFGLCKEGIKDGATMKTFCGTPEYLAPEVLEDND

YGRAVDWWGLGVVMYEMMCGRLPFYNQDHEKLFELILMEEIRFPRTLGPEAKSLLSGLLK

KDPKQRLGGGSEDAKEIMQ

>hAKT2_2120

TMNDFDYLKLLGKGTFGKVILVREKATGRYYAMKILRKEVIIAKDEVAHTVTESRVLQNT

RHPFLTALKYAFQTHDRLCFVMEYANGGELFFHLSRERVFTEERARFYGAEIVSALEYLH

SRDVVYRDIKLENLMLDKDGHIKITDFGLCKEGISDGATMKTFCGTPEYLAPEVLEDNDY

GRAVDWWGLGVVMYEMMCGRLPFYNQDHERLFELILMEEIRFPRTLSPEAKSLLAGLLKK

DPKQRLGGGPSDAKEVME

>hAKT3_2128

TMNDFDYLKLLGKGTFGKVILVREKASGKYYAMKILKKEVIIAKDEVAHTLTESRVLKNT

RHPFLTSLKYSFQTKDRLCFVMEYVNGGELFFHLSRERVFSEDRTRFYGAEIVSALDYLH

SGKIVYRDLKLENLMLDKDGHIKITDFGLCKEGITDAATMKTFCGTPEYLAPEVLEDNDY

GRAVDWWGLGVVMYEMMCGRLPFYNQDHEKLFELILMEDIKFPRTLSSDAKSLLSGLLIK

DPNKRLGGGPDDAKEIMR

>hALK_949

PRKNITLIRGLGHGAFGEVYEGQVSGMPNDPSPLQVAVKTLPEVCSEQDELDFLMEALII

SKFNHQNIVRCIGVSLQSLPRFILLELMAGGDLKSFLRETRPRPSQPSSLAMLDLLHVAR

DIACGCQYLEENHFIHRDIAARNCLLTCPGPGRVAKIGDFGMARDIYRASYYRKGGCAML

PVKWMPPEAFMEGIFTSKTDTWSFGVLLWEIFSLGYMPYPSKSNQEVLEFVTSGGRMDPP

KNCPGPVYRIMTQCWQHQPEDRPNFAIILERIEYC

>hAURa_2362

ALEDFEIGRPLGKGKFGNVYLAREKQSKFILALKVLFKAQLEKAGVEHQLRREVEIQSHL

RHPNILRLYGYFHDATRVYLILEYAPLGTVYRELQKLSKFDEQRTATYITELANALSYCH

SKRVIHRDIKPENLLLGSAGELKIADFGWSVHAPSSRRTTLCGTLDYLPPEMIEGRMHDE

KVDLWSLGVLCYEFLVGKPPFEANTYQETYKRISRVEFTFPDFVTEGARDLISRLLKHNP

SQRPMLREVLEHPWIT

>hAURb_2346

TIDDFEIGRPLGKGKFGNVYLAREKKSHFIVALKVLFKSQIEKEGVEHQLRREIEIQAHL

HHPNILRLYNYFYDRRRIYLILEYAPRGELYKELQKSCTFDEQRTATIMEELADALMYCH

GKKVIHRDIKPENLLLGLKGELKIADFGWSVHAPSLRRKTMCGTLDYLPPEMIEGRMHNE

KVDLWCIGVLCYELLVGNPPFESASHNETYRRIVKVDLKFPASVPTGAQDLISKLLRHNP

SERLPLAQVSAHPWVR

>hAURc_2382

TVDDFEIGRPLGKGKFGNVYLARLKESHFIVALKVLFKSQIEKEGLEHQLRREIEIQAHL

QHPNILRLYNYFHDARRVYLILEYAPRGELYKELQKSEKLDEQRTATIIEELADALTYCH

DKKVIHRDIKPENLLLGFRGEVKIADFGWSVHTPSLRRKTMCGTLDYLPPEMIEGRTYDE

KVDLWCIGVLCYELLVGYPPFESASHSETYRRILKVDVRFPLSMPLGARDLISRLLRYQP

LERLPLAQILKHPWVQ

>hAXL_929

DRHKVALGKTLGEGEFGAVMEGQLNQDDSILKVAVKTMKIAICTRSELEDFLSEAVCMKE

FDHPNVMRLIGVCFQGSERESFPAPVVILPFMKHGDLHSFLLYSRLGDQPVYLPTQMLVK

FMADIASGMEYLSTKRFIHRDLAARNCMLNENMSVCVADFGLSKKIYNGDYYRQGRIAKM

PVKWIAIESLADRVYTSKSDVWSFGVTMWEIATRGQTPYPGVENSEIYDYLRQGNRLKQP

ADCLDGLYALMSRCWELNPQDRPSFTELREDLENT

>hBLK_2315

PRQSLRLVRKLGSGQFGEVWMGYYKNNMKVAIKTLKEGTMSPEAFLGEANMMKALQHERL

VRLYAVVTKEPIYIVTEYMARGCLLDFLKTDEGSRLSLPRLIDMSAQIAEGMAYIERMNS

IHRDLRAANILVSEALCCKIADFGLARIIDSEYTAQEGAKFPIKWTAPEAIHFGVFTIKA

DVWSFGVLLMEVVTYGRVPYPGMSNPEVIRNLERGYRMPRPDTCPPELYRGVIAECWRSR

PEERPTFEFLQSVLEDF

>hBMX_2265

KREEITLLKELGSGQFGVVQLGKWKGQYDVAVKMIKEGSMSEDEFFQEAQTMMKLSHPKL

VKFYGVCSKEYPIYIVTEYISNGCLLNYLRSHGKGLEPSQLLEMCYDVCEGMAFLESHQF

IHRDLAARNCLVDRDLCVKVSDFGMTRYVLDDQYVSSVGTKFPVKWSAPEVFHYFKYSSK

SDVWAFGILMWEVFSLGKQPYDLYDNSQVVLKVSQGHRLYRPHLASDTIYQIMYSCWHEL

PEKRPTFQQLLSSIEPL

>hBRAF_1421

PDGQITVGQRIGSGSFGTVYKGKWHGDVAVKMLNVTAPTPQQLQAFKNEVGVLRKTRHVN

ILLFMGYSTKPQLAIVTQWCEGSSLYHHLHIIETKFEMIKLIDIARQTAQGMDYLHAKSI

IHRDLKSNNIFLHEDLTVKIGDFGLATVKSRWSGSHQFEQLSGSILWMAPEVIRMQDKNP

YSFQSDVYAFGIVLYELMTGQLPYSNINNRDQIIFMVGRGYLSPDLSKVRSNCPKAMKRL

MAECLKKKRDERPLFPQILASIELL

>hBRSK2_2283

YVGPYRLEKTLGKGQTGLVKLGVHCVTCQKVAIKIVNREKLSESVLMKVEREIAILKLIE

HPHVLKLHDVYENKKYLYLVLEHVSGGELFDYLVKKGRLTPKEARKFFRQIISALDFCHS

HSICHRDLKPENLLLDEKNNIRIADFGMASLQVGDSLLETSCGSPHYACPEVIRGEKYDG

RKADVWSCGVILFALLVGALPFDDDNLRQLLEKVKRGVFHMPHFIPPDCQSLLRGMSEVD

AARRLTLEHIQKHIWYI

>hBTK_2269

DPKDLTFLKELGTGQFGVVKYGKWRGQYDVAIKMIKEGSMSEDEFIEEAKVMMNLSHEKL

VQLYGVCTKQRPIFIITEYMANGCLLNYLREMRHRFQTQQLLEMCKDVCEAMEYLESKQF

LHRDLAARNCLVNDQGVVKVSDFGLSRYVLDDEYTSSVGSKFPVRWSPPEVLMYSKFSSK

SDIWAFGVLMWEIYSLGKMPYERFTNSETAEHIAQGLRLYRPHLASEKVYTIMYSCWHEK

ADERPTFKILLSNILDV

>hCAMK1_1725

IRDIYDFRDVLGTGAFSEVILAEDKRTQKLVAIKCIAKEALEGKEGSMENEIAVLHKIKH

PNIVALDDIYESGGHLYLIMQLVSGGELFDRIVEKGFYTERDASRLIFQVLDAVKYLHDL

GIVHRDLKPENLLYYSLDEDSKIMISDFGLSKMEDPGSVLSTACGTPGYVAPEVLAQKPY

SKAVDCWSIGVIAYILLCGYPPFYDENDAKLFEQILKAEYEFDSPYWDDISDSAKDFIRH

LMEKDPEKRFTCEQALQHPWIA

>hCAMK2A_1489

FTEEYQLFEELGKGAFSVVRRCVKVLAGQEYAAKIINTKKLSARDHQKLEREARICRLLK

HPNIVRLHDSISEEGHHYLIFDLVTGGELFEDIVAREYYSEADASHCIQQILEAVLHCHQ

MGVVHRDLKPENLLLASKLKGAAVKLADFGLAIEVEGEQQAWFGFAGTPGYLSPEVLRKD

PYGKPVDLWACGVILYILLVGYPPFWDEDQHRLYQQIKAGAYDFPSPEWDTVTPEAKDLI

NKMLTINPSKRITAAEALKHPWIS

>hCAMK2B_1515

FTDEYQLYEDIGKGAFSVVRRCVKLCTGHEYAAKIINTKKLSARDHQKLEREARICRLLK

HSNIVRLHDSISEEGFHYLVFDLVTGGELFEDIVAREYYSEADASHCIQQILEAVLHCHQ

MGVVHRDLKPENLLLASKCKGAAVKLADFGLAIEVQGDQQAWFGFAGTPGYLSPEVLRKE

AYGKPVDIWACGVILYILLVGYPPFWDEDQHKLYQQIKAGAYDFPSPEWDTVTPEAKNLI

NQMLTINPAKRITAHEALKHPWVC

>hCAMK2D_1471

FTDEYQLFEELGKGAFSVVRRCMKIPTGQGYAAKIINTKKLSARDHQKLEREARICRLLK

HPNIVRLHDSISEEGFHYLVFDLVTGGELFEDIVAREYYSEADASHCIQQILESVNHCHL

NGIVHRDLKPENLLLASKSKGAAVKLADFGLAIEVQGDQQAWFGFAGTPGYLSPEVLRKD

PYGKPVDMWACGVILYILLVGYPPFWDEDQHRLYQQIKAGAYDFPSPEWDTVTPEAKDLI

NKMLTINPAKRITASEALKHPWIC

>hCAMK2G_1496

FTDDYQLFEELGKGAFSVVRRCVKKTSTQEYAAKIINTKKLSARDHQKLEREARICRLLK

HPNIVRLHDSISEEGFHYLVFDLVTGGELFEDIVAREYYSEADASHCIHQILESVNHIHQ

HDIVHRDLKPENLLLASKCKGAAVKLADFGLAIEVQGEQQAWFGFAGTPGYLSPEVLRKD

PYGKPVDIWACGVILYILLVGYPPFWDEDQHKLYQQIKAGAYDFPSPEWDTVTPEAKNLI

NQMLTINPAKRITADQALKHPWVC

>hCAMK4_1879

LSDFFEVESELGRGATSIVYRCKQKGTQKPYALKVLKKTVDKKIVRTEIGVLLRLSHPNI

IKLKEIFETPTEISLVLELVTGGELFDRIVEKGYYSERDAADAVKQILEAVAYLHENGIV

HRDLKPENLLYATPAPDAPLKIADFGLSKIVEHQVLMKTVCGTPGYCAPEILRGCAYGPE

VDMWSVGIITYILLCGFEPFYDERGDQFMFRRILNCEYYFISPWWDEVSLNAKDLVRKLI

VLDPKKRLTTFQALQHPWVT

>hCAMKK1_655

QLNQYKLQSEIGKGAYGVVRLAYNESEDRHYAMKVLSKKKLLKQYGFPRRPPPRGSQAAQ

GGPAKQLLPLERVYQEIAILKKLDHVNVVKLIEVLDDPAEDNLYLVFDLLRKGPVMEVPC

DKPFSEEQARLYLRDVILGLEYLHCQKIVHRDIKPSNLLLGDDGHVKIADFGVSNQFEGN

DAQLSSTAGTPAFMAPEAISDSGQSFSGKALDVWATGVTLYCFVYGKCPFIDDFILALHR

KIKNEPVVFPEEPEISEELKDLILKMLDKNPETRIGVPDIKLHPWVT

>hCDC42BPA_1153

HREDFEILKVIGRGAFGEVAVVKLKNADKVFAMKILNKWEMLKRAETACFREERDVLVNG

DNKWITTLHYAFQDDNNLYLVMDYYVGGDLLTLLSKFEDRLPEDMARFYLAEMVIAIDSV

HQLHYVHRDIKPDNILMDMNGHIRLADFGSCLKLMEDGTVQSSVAVGTPDYISPEILQAM

EDGKGRYGPECDWWSLGVCMYEMLYGETPFYAESLVETYGKIMNHKERFQFPAQVTDVSE

NAKDLIRRLICSREHRLGQNGIEDFKKHP

>hCDC7_4

LSNVFKIEDKIGEGTFSSVYLATAQLQVGPEEKIALKHLIPTSHPIRIAAELQCLTVAGG

QDNVMGVKYCFRKNDHVVIAMPYLEHESFLDILNSLSFQEVREYMLNLFKALKRIHQFGI

VHRDVKPSNFLYNRRLKKYALVDFGLAQGTHDTKIELLKFVQSEAQQERCSQNKSCPASL

TCDCYATDKVCSICLSRRQQVAPRAGTPGFRAPEVLTKCPNQTTAIDMWSAGVIFLSLLS

GRYPFYKASDDLTALAQIMTIRGSRETIQAAKTFGKSILCSKEVPAQDLRKLCERLRGMD

SSTPKLTSDIQGHASHQPAISEKTDHKASCLVQTPPGQYSGNSFKKGDSNSCEHCFDEYN

TNLEGWNEVPDEAYDLLDKLLDLNPASRITAEEALLHPFFK

>hCDK1_416

MEDYTKIEKIGEGTYGVVYKGRHKTTGQVVAMKKIRLESEEEGVPSTAIREISLLKELRH

PNIVSLQDVLMQDSRLYLIFEFLSMDLKKYLDSIPPGQYMDSSLVKSYLYQILQGIVFCH

SRRVLHRDLKPQNLLIDDKGTIKLADFGLARAFGIPIRVYTHEVVTLWYRSPEVLLGSAR

YSTPVDIWSIGTIFAELATKKPLFHGDSEIDQLFRIFRALGTPNNEVWPEVESLQDYKNT

FPKWKPGSLASHVKNLDENGLDLLSKMLIYDPAKRISGKMALNHPYFN

>hCDK2_583

MENFQKVEKIGEGTYGVVYKARNKLTGEVVALKKIRLDTETEGVPSTAIREISLLKELNH

PNIVKLLDVIHTENKLYLVFEFLHQDLKKFMDASALTGIPLPLIKSYLFQLLQGLAFCHS

HRVLHRDLKPQNLLINTEGAIKLADFGLARAFGVPVRTYTHEVVTLWYRAPEILLGCKYY

STAVDIWSLGCIFAEMVTRRALFPGDSEIDQLFRIFRTLGTPDEVVWPGVTSMPDYKPSF

PKWARQDFSKVVPPLDEDGRSLLSQMLHYDPNKRISAKAALAHPFFQ

>hCDK3_601

MDMFQKVEKIGEGTYGVVYKAKNRETGQLVALKKIRLDLEMEGVPSTAIREISLLKELKH

PNIVRLLDVVHNERKLYLVFEFLSQDLKKYMDSTPGSELPLHLIKSYLFQLLQGVSFCHS

HRVIHRDLKPQNLLINELGAIKLADFGLARAFGVPLRTYTHEVVTLWYRAPEILLGSKFY

TTAVDIWSIGCIFAEMVTRKALFPGDSEIDQLFRIFRMLGTPSEDTWPGVTQLPDYKGSF

PKWTRKGLEEIVPNLEPEGRDLLMQLLQYDPSQRITAKTALAHPYFS

>hCDK4_340

ATSRYEPVAEIGVGAYGTVYKARDPHSGHFVALKSVRVPNGGGGGGGLPISTVREVALLR

RLEAFEHPNVVRLMDVCATSRTDREIKVTLVFEHVDQDLRTYLDKAPPPGLPAETIKDLM

RQFLRGLDFLHANCIVHRDLKPENILVTSGGTVKLADFGLARIYSYQMALTPVVVTLWYR

APEVLLQSTYATPVDMWSVGCIFAEMFRRKPLFCGNSEADQLGKIFDLIGLPPEDDWPRD

VSLPRGAFPPRGPRPVQSVVPEMEESGAQLLLEMLTFNPHKRISAFRALQHSYLH

>hCDK5_607

MQKYEKLEKIGEGTYGTVFKAKNRETHEIVALKRVRLDDDDEGVPSSALREICLLKELKH

KNIVRLHDVLHSDKKLTLVFEFCDQDLKKYFDSCNGDLDPEIVKSFLFQLLKGLGFCHSR

NVLHRDLKPQNLLINRNGELKLADFGLARAFGIPVRCYSAEVVTLWYRPPDVLFGAKLYS

TSIDMWSAGCIFAELANAGRPLFPGNDVDDQLKRIFRLLGTPTEEQWPSMTKLPDYKPYP

MYPATTSLVNVVPKLNATGRDLLQNLLKCNPVQRISAEEALQHPYFS

>hCDK6_421

ADQQYECVAEIGEGAYGKVFKARDLKNGGRFVALKRVRVQTGEEGMPLSTIREVAVLRHL

ETFEHPNVVRLFDVCTVSRTDRETKLTLVFEHVDQDLTTYLDKVPEPGVPTETIKDMMFQ

LLRGLDFLHSHRVVHRDLKPQNILVTSSGQIKLADFGLARIYSFQMALTSVVVTLWYRAP

EVLLQSSYATPVDLWSVGCIFAEMFRRKPLFRGSSDVDQLGKILDVIGLPGEEDWPRDVA

LPRQAFHSKSAQPIEKFVTDIDELGKDLLLKCLTFNPAKRISAYSALSHPYFQ

>hCDK7_564

RAKRYEKLDFLGEGQFATVYKARDKNTNQIVAIKKIKLGHRSEAKDGINRTALREIKLLQ

ELSHPNIIGLLDAFGHKSNISLVFDFMETDLEVIIKDNSLVLTPSHIKAYMLMTLQGLEY

LHQHWILHRDLKPNNLLLDENGVLKLADFGLAKSFGSPNRAYTHQVVTRWYRAPELLFGA

RMYGVGVDMWAVGCILAELLLRVPFLPGDSDLDQLTRIFETLGTPTEEQWPDMCSLPDYV

TFKSFPGIPLHHIFSAAGDDLLDLIQGLFLFNPCARITATQALKMKYFS

>hCDK9_246

EVSKYEKLAKIGQGTFGEVFKARHRKTGQKVALKKVLMENEKEGFPITALREIKILQLLK

HENVVNLIEICRTKASPYNRCKGSIYLVFDFCEHDLAGLLSNVLVKFTLSEIKRVMQMLL

NGLYYIHRNKILHRDMKAANVLITRDGVLKLADFGLARAFSLAKNSQPNRYTNRVVTLWY

RPPELLLGERDYGPPIDLWGAGCIMAEMWTRSPIMQGNTEQHQLALISQLCGSITPEVWP

NVDNYELYEKLELVKGQKRKVKDRLKAYVRDPYALDLIDKLLVLDPAQRIDSDDALNHDF

FW

>hCHUK_513

AGGPWEMRERLGTGGFGNVCLYQHRELDLKIAIKSCRLELSTKNRERWCHEIQIMKKLNH

ANVVKACDVPEELNILIHDVPLLAMEYCSGGDLRKLLNKPENCCGLKESQILSLLSDIGS

GIRYLHENKIIHRDLKPENIVLQDVGGKIIHKIIDLGYAKDVDQGSLCTSFVGTLQYLAP

ELFENKPYTATVDYWSFGTMVFECIAGYRPFLHHLQPFTWHEKIKKKDPKCIFACEEMSG

EVRFSSHLPQPNSLCSLIVEPMENWLQLMLNWDPQQRGGPVDLTLKQPRC

>hCLK1_121

LSARYEIVDTLGEGAFGKVVECIDHKAGGRHVAVKIVKNVDRYCEAARSEIQVLEHLNTT

DPNSTFRCVQMLEWFEHHGHICIVFELLGLSTYDFIKENGFLPFRLDHIRKMAYQICKSV

NFLHSNKLTHTDLKPENILFVQSDYTEAYNPKIKRDERTLINPDIKVVDFGSATYDDEHH

STLVSTRHYRAPEVILALGWSQPCDVWSIGCILIEYYLGFTVFPTHDSKEHLAMMERILG

PLPKHMIQKTRKRKYFHHDRLDWDEHSSAGRYVSRACKPLKEFMLSQDVEHERLFDLIQK

MLEYDPAKRITLREALKHPFFD

>hCSF1R_55

PRNNLQFGKTLGAGAFGKVVEATAFGLGKEDAVLKVAVKMLKSTAHADEKEALMSELKIM

SHLGQHENIVNLLGACTHGGPVLVITEYCCYGDLLNFLRRKAEAMLGPSLSPVSTSSNDS

FSEQDLDKEDGRPLELRDLLHFSSQVAQGMAFLASKNCIHRDVAARNVLLTNGHVAKIGD

FGLARDIMNDSNYIVKGNARLPVKWMAPESIFDCVYTVQSDVWSYGILLWEIFSLGLNPY

PGILVNSKFYKLVKDGYQMAQPAFAPKNIYSIMQACWALEPTHRPTFQQICSFLQEQ

>hCSK_2470

NMKELKLLQTIGKGEFGDVMLGDYRGNKVAVKCIKNDATAQAFLAEASVMTQLRHSNLVQ

LLGVIVEEKGGLYIVTEYMAKGSLVDYLRSRGRSVLGGDCLLKFSLDVCEAMEYLEGNNF

VHRDLAARNVLVSEDNVAKVSDFGLTKEASSTQDTGKLPVKWTAPEALREKKFSTKSDVW

SFGILLWEIYSFGRVPYPRIPLKDVVPRVEKGYKMDAPDGCPPAVYEVMKNCWHLDAAMR

PSFLQLREQLEHI

>hCSNK1A1_1040

VGGKYKLVRKIGSGSFGDIYLAINITNGEEVAVKLESQKARHPQLLYESKLYKILQGGVG

IPHIRWYGQEKDYNVLVMDLLGPSLEDLFNFCSRRFTMKTVLMLADQMISRIEYVHTKNF

IHRDIKPDNFLMGIGRHCNKLFLIDFGLAKKYRDNRTRQHIPYREDKNLTGTARYASINA

HLGIEQSRRDDMESLGYVLMYFNRTSLPWQGLKAATKKQKYEKISEKKMSTPVEVLCKGF

PAEFAMYLNYCRGLRFEEAPDYMYLRQLFRIL

>hCSNK1D_2523

VGNRYRLGRKIGSGSFGDIYLGTDIAAGEEVAIKLECVKTKHPQLHIESKIYKMMQGGVG

IPTIRWCGAEGDYNVMVMELLGPSLEDLFNFCSRKFSLKTVLLLADQMISRIEYIHSKNF

IHRDVKPDNFLMGLGKKGNLVYIIDFGLAKKYRDARTHQHIPYRENKNLTGTARYASINT

HLGIEQSRRDDLESLGYVLMYFNLGSLPWQGLKAATKRQKYERISEKKMSTPIEVL

>hCSNK1E_2527

VGNKYRLGRKIGSGSFGDIYLGANIASGEEVAIKLECVKTKHPQLHIESKFYKMMQGGVG

IPSIKWCGAEGDYNVMVMELLGPSLEDLFNFCSRKFSLKTVLLLADQMISRIEYIHSKNF

IHRDVKPDNFLMGLGKKGNLVYIIDFGLAKKYRDARTHQHIPYRENKNLTGTARYASINT

HLGIEQSRRDDLESLGYVLMYFNLGSLPWQGLKAATKRQKYERISEKKMSTPIEVL

>hCSNK1G1_1165

VGPNFRVGKKIGCGNFGELRLGKNLYTNEYVAIKLEPIKSRAPQLHLEYRFYKQLGSAGE

GLPQVYYFGPCGKYNAMVLELLGPSLEDLFDLCDRTFTLKTVLMIAIQLLSRMEYVHSKN

LIYRDVKPENFLIGRQGNKKEHVIHIIDFGLAKEYIDPETKKHIPYREHKSLTGTARYMS

INTHLGKEQSRRDDLEALGHMFMYFLRGSLPWQGLKADTLKERYQKIGDTKRNTPIEALC

ENFPEEMATYLRYVRRLDFFEKPDYEYLR

>hCSNK1G2_1221

VGPNFRVGKKIGCGNFGELRLGKNLYTNEYVAIKLEPIKSRAPQLHLEYRFYKQLSATEG

VPQVYYFGPCGKYNAMVLELLGPSLEDLFDLCDRTFTLKTVLMIAIQLITRMEYVHTKSL

IYRDVKPENFLVGRPGTKRQHAIHIIDFGLAKEYIDPETKKHIPYREHKSLTGTARYMSI

NTHLGKEQSRRDDLEALGHMFMYFLRGSLPWQGLKADTLKERYQKIGDTKRATPIEVLCE

NFPEEMATYLRYVRRLDFFEKPDYDYLR

>hCSNK2A1_457

NQDDYQLVRKLGRGKYSEVFEAINITNNEKVVVKILKPVKKKKIKREIKILENLRGGPNI

ITLADIVKDPVSRTPALVFEHVNNTDFKQLYQTLTDYDIRFYMYEILKALDYCHSMGIMH

RDVKPHNVMIDHEHRKLRLIDWGLAEFYHPGQEYNVRVASRYFKGPELLVDYQMYDYSLD

MWSLGCMLASMIFRKEPFFHGHDNYDQLVRIAKVLGTEDLYDYIDKYNIELDPRFNDILG

RHSRKRWERFVHSENQHLVSPEALDFLDKLLRYDHQSRLTAREAMEHPYFY

>hCSNK2A2_468

NQDDYQLVRKLGRGKYSEVFEAINITNNERVVVKILKPVKKKKIKREVKILENLRGGTNI

IKLIDTVKDPVSKTPALVFEYINNTDFKQLYQILTDFDIRFYMYELLKALDYCHSKGIMH

RDVKPHNVMIDHQQKKLRLIDWGLAEFYHPAQEYNVRVASRYFKGPELLVDYQMYDYSLD

MWSLGCMLASMIFRREPFFHGQDNYDQLVRIAKVLGTEELYGYLKKYHIDLDPHFNDILG

QHSRKRWENFIHSENRHLVSPEALDLLDKLLRYDHQQRLTAKEAMEHPYFY

>hChk1_1758

FVEDWDLVQTLGEGAYGEVQLAVNRVTEEAVAVKIVDMKRAVDCPENIKKEICINKMLNH

ENVVKFYGHRREGNIQYLFLEYCSGGELFDRIEPDIGMPEPDAQRFFHQLMAGVVYLHGI

GITHRDIKPENLLLDERDNLKISDFGLATVFRYNNRERLLNKMCGTLPYVAPELLKRREF

HAEPVDVWSCGIVLTAMLAGELPWDQPSDSCQEYSDWKEKKTYLNPWKKIDSAPLALLHK

ILVENPSARITIPDIKKDRWYN

>hChk2_1009

LRDEYIMSKTLGSGACGEVKLAFERKTCKKVAIKIISKRKFAIGSAREADPALNVETEIE

ILKKLNHPCIIKIKNFFDAEDYYIVLELMEGGELFDKVVGNKRLKEATCKLYFYQMLLAV

QYLHENGIIHRDLKPENVLLSSQEEDCLIKITDFGHSKILGETSLMRTLCGTPTYLAPEV

LVSVGTAGYNRAVDCWSLGVILFICLSGYPPFSEHRTQVSLKDQITSGKYNFIPEVWAEV

SEKALDLVKKLLVVDPKARFTTEEALRHPWLQ

>hDAPK3_1202

VEDHYEMGEELGSGQFAIVRKCRQKGTGKEYAAKFIKKRRLSSSRRGVSREEIEREVNIL

REIRHPNIITLHDIFENKTDVVLILELVSGGELFDFLAEKESLTEDEATQFLKQILDGVH

YLHSKRIAHFDLKPENIMLLDKNVPNPRIKLIDFGIAHKIEAGNEFKNIFGTPEFVAPEI

VNYEPLGLEADMWSIGVITYILLSGASPFLGETKQETLTNISAVNYDFDEEYFSNTSELA

KDFIRRLLVKDPKRRMTIAQSLEHSWIK

>hDDR2_382

PRKLLTFKEKLGEGQFGEVHLCEVEGMEKFKDKDFALDVSANQPVLVAVKMLRADANKNA

RNDFLKEIKIMSRLKDPNIIHLLSVCITDDPLCMITEYMENGDLNQFLSRHEPPNSSSSD

VRTVSYTNLKFMATQIASGMKYLSSLNFVHRDLATRNCLVGKNYTIKIADFGMSRNLYSG

DYYRIQGRAVLPIRWMSWESILLGKFTTASDVWAFGVTLWETFTFCQEQPYSQLSDEQVI

ENTGEFFRDQGRQTYLPQPAICPDSVYKLMLSCWRRDTKNRPSFQEIHLLLLQQ

>hDYRK1A_113

WMDRYEIDSLIGKGSFGQVVKAYDRVEQEWVAIKIIKNKKAFLNQAQIEVRLLELMNKHD

TEMKYYIVHLKRHFMFRNHLCLVFEMLSYNLYDLLRNTNFRGVSLNLTRKFAQQMCTALL

FLATPELSIIHCDLKPENILLCNPKRSAIKIVDFGSSCQLGQRIYQYIQSRFYRSPEVLL

GMPYDLAIDMWSLGCILVEMHTGEPLFSGANEVDQMNKIVEVLGIPPAHILDQAPKARKF

FEKLPDGTWNLKKTKDGKREYKPPGTRKLHNILGVETGGPGGRRAGESGHTVADYLKFKD

LILRMLDYDPKTRIQPYYALQHSFFK

>hDYRK2_173

VAYRYEVLKVIGKGSFGQVVKAYDHKVHQHVALKMVRNEKRFHRQAAEEIRILEHLRKQD

KDNTMNVIHMLENFTFRNHICMTFELLSMNLYELIKKNKFQGFSLPLVRKFAHSILQCLD

ALHKNRIIHCDLKPENILLKQQGRSGIKVIDFGSSCYEHQRVYTYIQSRFYRAPEVILGA

RYGMPIDMWSLGCILAELLTGYPLLPGEDEGDQLACMIELLGMPSQKLLDASKRAKNFVS

SKGYPRYCTVTTLSDGSVVLNGGRSRRGKLRGPPESREWGNALKGCDDPLFLDFLKQCLE

WDPAVRMTPGQALRHPWLR

>hDYRK3_167

LAYRYEVLKIIGKGSFGQVARVYDHKLRQYVALKMVRNEKRFHRQAAEEIRILEHLKKQD

KTGSMNVIHMLESFTFRNHVCMAFELLSIDLYELIKKNKFQGFSVQLVRKFAQSILQSLD

ALHKNKIIHCDLKPENILLKHHGRSSTKVIDFGSSCFEYQKLYTYIQSRFYRAPEIILGS

RYSTPIDIWSFRCILAELLTGQPLFPGEDEGDQLACMMELLGMPPPKLLEQSKRAKYFIN

SKGIPRYCSVTTQADGRVVLVGGRSRRGKKRGPPGSKDWGTALKGCDDYLFIEFLKRCLH

WDPSARLTPAQALRHPWIS

>hEGFR_1553

KETEFKKIKVLGSGAFGTVYKGLWIPEGEKVKIPVAIKELREATSPKANKEILDEAYVMA

SVDNPHVCRLLGICLTSTVQLITQLMPFGCLLDYVREHKDNIGSQYLLNWCVQIAKGMNY

LEDRRLVHRDLAARNVLVKTPQHVKITDFGLAKLLGAEEKEYHAEGGKVPIKWMALESIL

HRIYTHQSDVWSYGVTVWELMTFGSKPYDGIPASEISSILEKGERLPQPPICTIDVYMIM

VKCWMIDADSRPKFRELIIEFSKM

>hEPHA1_1462

DPAWLMVDTVIGEGEFGEVYRGTLRLPSQDCKTVAIKTLKDTSPGGQWWNFLREATIMGQ

FSHPHILHLEGVVTKRKPIMIITEFMENGALDAFLREREDQLVPGQLVAMLQGIASGMNY

LSNHNYVHRDLAARNILVNQNLCCKVSDFGLTRLLDDFDGTYETQGGKIPIRWTAPEAIA

HRIFTTASDVWSFGIVMWEVLSFGDKPYGEMSNQEVMKSIEDGYRLPPPVDCPAPLYELM

KNCWAYDRARRPHFQKLQAHLEQL

>hEPHA2_1318

HPSCVTRQKVIGAGEFGEVYKGMLKTSSGKKEVPVAIKTLKAGYTEKQRVDFLGEAGIMG

QFSHHNIIRLEGVISKYKPMMIITEYMENGALDKFLREKDGEFSVLQLVGMLRGIAAGMK

YLANMNYVHRDLAARNILVNSNLVCKVSDFGLSRVLEDDPEATYTTSGGKIPIRWTAPEA

ISYRKFTSASDVWSFGIVMWEVMTYGERPYWELSNHEVMKAINDGFRLPTPMDCPSAIYQ

LMMQCWQQERARRPKFADIVSILDKL

>hEPHA3_1400

DATNISIDKVVGAGEFGEVCSGRLKLPSKKEISVAIKTLKVGYTEKQRRDFLGEASIMGQ

FDHPNIIRLEGVVTKSKPVMIVTEYMENGSLDSFLRKHDAQFTVIQLVGMLRGIASGMKY

LSDMGYVHRDLAARNILINSNLVCKVSDFGLSRVLEDDPEAAYTTRGGKIPIRWTSPEAI

AYRKFTSASDVWSYGIVLWEVMSYGERPYWEMSNQDVIKAVDEGYRLPPPMDCPAALYQL

MLDCWQKDRNNRPKFEQIVSILDKL

>hEPHA4_1410

DASCIKIEKVIGVGEFGEVCSGRLKVPGKREICVAIKTLKAGYTDKQRRDFLSEASIMGQ

FDHPNIIHLEGVVTKCKPVMIITEYMENGSLDAFLRKNDGRFTVIQLVGMLRGIGSGMKY

LSDMSYVHRDLAARNILVNSNLVCKVSDFGMSRVLEDDPEAAYTTRGGKIPIRWTAPEAI

AYRKFTSASDVWSYGIVMWEVMSYGERPYWDMSNQDVIKAIEEGYRLPPPMDCPIALHQL

MLDCWQKERSDRPKFGQIVNMLDKL

>hEPHB1_1240

DVSFVKIEEVIGAGEFGEVYKGRLKLPGKREIYVAIKTLKAGYSEKQRRDFLSEASIMGQ

FDHPNIIRLEGVVTKSRPVMIITEFMENGALDSFLRQNDGQFTVIQLVGMLRGIAAGMKY

LAEMNYVHRDLAARNILVNSNLVCKVSDFGLSRYLQDDTSDPTYTSSLGGKIPVRWTAPE

AIAYRKFTSASDVWSYGIVMWEVMSFGERPYWDMSNQDVINAIEQDYRLPPPMDCPAALH

QLMLDCWQKDRNSRPRFAEIVNTLDKM

>hEPHB2_1229

DISCVKIEQVIGAGEFGEVCSGHLKLPGKREIFVAIKTLKSGYTEKQRRDFLSEASIMGQ

FDHPNVIHLEGVVTKSTPVMIITEFMENGSLDSFLRQNDGQFTVIQLVGMLRGIAAGMKY

LADMNYVHRDLAARNILVNSNLVCKVSDFGLSRFLEDDTSDPTYTSALGGKIPIRWTAPE

AIQYRKFTSASDVWSYGIVMWEVMSYGERPYWDMTNQDVINAIEQDYRLPPPMDCPSALH

QLMLDCWQKDRNHRPKFGQIVNTLDKM

>hEPHB3_1256

DVSCVKIEEVIGAGEFGEVCRGRLKQPGRREVFVAIKTLKVGYTERQRRDFLSEASIMGQ

FDHPNIIRLEGVVTKSRPVMILTEFMENCALDSFLRLNDGQFTVIQLVGMLRGIAAGMKY

LSEMNYVHRDLAARNILVNSNLVCKVSDFGLSRFLEDDPSDPTYTSSLGGKIPIRWTAPE

AIAYRKFTSASDVWSYGIVMWEVMSYGERPYWDMSNQDVINAVEQDYRLPPPMDCPTALH

QLMLDCWVRDRNLRPKFSQIVNTLDKL

>hEPHB4_1246

DVSYVKIEEVIGAGEFGEVCRGRLKAPGKKESCVAIKTLKGGYTERQRREFLSEASIMGQ

FEHPNIIRLEGVVTNSMPVMILTEFMENGALDSFLRLNDGQFTVIQLVGMLRGIASGMRY

LAEMSYVHRDLAARNILVNSNLVCKVSDFGLSRFLEENSSDPTYTSSLGGKIPIRWTAPE

AIAFRKFTSASDAWSYGIVMWEVMSFGERPYWDMSNQDVINAIEQDYRLPPPPDCPTSLH

QLMLDCWQKDRNARPRFPQVVSALDKM

>hERK1_388

VGPRYTNLSYIGEGAYGMVCSAYDNVNKVRVAIKKISPFEHQTYCQRTLREIKILLRFRH

ENIIGINDIIRAPTIEQMKDVYIVQDLMETDLYKLLKTQHLSNDHICYFLYQILRGLKYI

HSANVLHRDLKPSNLLLNTTCDLKICDFGLARVADPDHDHTGFLTEYVATRWYRAPEIML

NSKGYTKSIDIWSVGCILAEMLSNRPIFPGKHYLDQLNHILGILGSPSQEDLNCIINLKA

RNYLLSLPHKNKVPWNRLFPNADSKALDLLDKMLTFNPHKRIEVEQALAHPYLE

>hERK1_404

VGPRYTQLQYIGEGAYGMVSSAYDHVRKTRVAIKKISPFEHQTYCQRTLREIQILLRFRH

ENVIGIRDILRASTLEAMRDVYIVQDLMETDLYKLLKSQQLSNDHICYFLYQILRGLKYI

HSANVLHRDLKPSNLLINTTCDLKICDFGLARIADPEHDHTGFLTEYVATRWYRAPEIML

NSKGYTKSIDIWSVGCILAEMLSNRPIFPGKHYLDQLNHILGILGSPSQEDLNCIINMKA

RNYLQSLPSKTKVAWAKLFPKSDSKALDLLDRMLTFNPNKRITVEEALAHPYLE

>hFAK1_1711

QRERIELGRCIGEGQFGDVHQGIYMSPENPALAVAIKTCKNCTSDSVREKFLQEALTMRQ

FDHPHIVKLIGVITENPVWIIMELCTLGELRSFLQVRKYSLDLASLILYAYQLSTALAYL

ESKRFVHRDIAARNVLVSSNDCVKLGDFGLSRYMEDSTYYKASKGKLPIKWMAPESINFR

RFTSASDVWMFGVCMWEILMHGVKPFQGVKNNDVIGRIENGERLPMPPNCPPTLYSLMTK

CWAYDPSRRPRFTELKAQLSTI

>hFER_1983

SHEDVILGELLGKGNFGEVYKGTLKDKTSVAVKTCKEDLPQELKIKFLQEAKILKQYDHP

NIVKLIGVCTQRQPVYIIMELVSGGDFLTFLRRKKDELKLKQLVKFSLDAAAGMLYLESK

NCIHRDLAARNCLVGENNVLKISDFGMSRQEDGGVYSSSGLKQIPIKWTAPEALNYGRYS

SESDVWSFGILLWETFSLGVCPYPGMTNQQAREQVERGYRMSAPQHCPEDISKIMMKCWD

YKPENRPKFSELQKELTII

>hFES_1796

NHEDLVLGEQIGRGNFGEVFSGRLRADNTLVAVKSCRETLPPDLKAKFLQEARILKQYSH

PNIVRLIGVCTQKQPIYIVMELVQGGDFLTFLRTEGARLRVKTLLQMVGDAAAGMEYLES

KCCIHRDLAARNCLVTEKNVLKISDFGMSREEADGVYAASGGSRQVPVKWTAPEALNYGR

YSSESDVWSFGILLWETFSLGASPYPNLSNQQTREFVEKGGRLPCPELCPDAVFRLMEQC

WAYEPGQRPSFSTIYQELQSI

>hFGFR1_708

PRDRLVLGKPLGEGCFGQVVLAEAIGLDKDKPNRVTKVAVKMLKSDATEKDLSDLISEME

MMKMIGKHKNIINLLGACTQDGPLYVIVEYASKGNLREYLQARRPPGLEYCYNPSHNPEE

QLSSKDLVSCAYQVARGMEYLASKKCIHRDLAARNVLVTEDNVMKIADFGLARDIHHIDY

YKKTTNGRLPVKWMAPEALFDRIYTHQSDVWSFGVLLWEIFTLGGSPYPGVPVEELFKLL

KEGHRMDKPSNCTNELYMMMRDCWHAVPSQRPTFKQLVEDLDRI

>hFGFR2_724

PRDKLTLGKPLGEGCFGQVVMAEAVGIDKDKPKEAVTVAVKMLKDDATEKDLSDLVSEME

MMKMIGKHKNIINLLGACTQDGPLYVIVEYASKGNLREYLRARRPPGMEYSYDINRVPEE

QMTFKDLVSCTYQLARGMEYLASQKCIHRDLAARNVLVTENNVMKIADFGLARDINNIDY

YKKTTNGRLPVKWMAPEALFDRVYTHQSDVWSFGVLMWEIFTLGGSPYPGIPVEELFKLL

KEGHRMDKPANCTNELYMMMRDCWHAVPSQRPTFKQLVEDLDRI

>hFGFR3_718

SRARLTLGKPLGEGCFGQVVMAEAIGIDKDRAAKPVTVAVKMLKDDATDKDLSDLVSEME

MMKMIGKHKNIINLLGACTQGGPLYVLVEYAAKGNLREFLRARRPPGLDYSFDTCKPPEE

QLTFKDLVSCAYQVARGMEYLASQKCIHRDLAARNVLVTEDNVMKIADFGLARDVHNLDY

YKKTTNGRLPVKWMAPEALFDRVYTHQSDVWSFGVLLWEIFTLGGSPYPGIPVEELFKLL

KEGHRMDKPANCTHDLYMIMRECWHAAPSQRPTFKQLVEDLDRV

>hFGFR4_699

PRDRLVLGKPLGEGCFGQVVRAEAFGMDPARPDQASTVAVKMLKDNASDKDLADLVSEME

VMKLIGRHKNIINLLGVCTQEGPLYVIVECAAKGNLREFLRARRPPGPDLSPDGPRSSEG

PLSFPVLVSCAYQVARGMQYLESRKCIHRDLAARNVLVTEDNVMKIADFGLARGVHHIDY

YKKTSNGRLPVKWMAPEALFDRVYTHQSDVWSFGILLWEIFTLGGSPYPGIPVEELFSLL

REGHRMDRPPHCPPELYGLMRECWHAAPSQRPTFKQLVEALDKV

>hFGR_2262

SRSSITLERRLGTGCFGDVWLGTWNGSTKVAVKTLKPGTMSPKAFLEEAQVMKLLRHDKL

VQLYAVVSEEPIYIVTEFMCHGSLLDFLKNPEGQDLRLPQLVDMAAQVAEGMAYMERMNY

IHRDLRAANILVGERLACKIADFGLARLIKDDEYNPCQGSKFPIKWTAPEAALFGRFTIK

SDVWSFGILLTELITKGRIPYPGMNKREVLEQVEQGYHMPCPPGCPASLYEAMEQTWRLD

PEERPTFEYLQSFLEDY

>hFLT3_36

PRENLEFGKVLGSGAFGKVMNATAYGISKTGVSIQVAVKMLKEKADSSEREALMSELKMM

TQLGSHENIVNLLGACTLSGPIYLIFEYCCYGDLLNYLRSKREKFHRTWTEHSEDEIEYE

NQKRLEEEEDLNVLTFEDLLCFAYQVAKGMEFLEFKSCVHRDLAARNVLVTHGKVVKICD

FGLARDIMSDSNYVVRGNARLPVKWMAPESLFEGIYTIKSDVWSYGILLWEIFSLGVNPY

PGIPVDANFYKLIQNGFKMDQPFYATEEIYIIMQSCWAFDSRKRPSFPNLTSFLGCQ

>hFYN_2194

PRESLQLIKRLGNGQFGEVWMGTWNGNTKVAIKTLKPGTMSPESFLEEAQIMKKLKHDKL

VQLYAVVSEEPIYIVTEYMNKGSLLDFLKDGEGRALKLPNLVDMAAQVAAGMAYIERMNY

IHRDLRSANILVGNGLICKIADFGLARLIEDNEYTARQGAKFPIKWTAPEAALYGRFTIK

SDVWSFGILLTELVTKGRVPYPGMNNREVLEQVERGYRMPCPQDCPISLHELMIHCWKKD

PEERPTFEYLQSFLEDY

>hGAK_791

GELRLRVRRVLAEGGFAFVYEAQDVGSGREYALKRLLSNEEEKNRAIIQEVCFMKKLSGH

PNIVQFCSAASIGKEESDTGQAEFLLLTELCKGQLVEFLKKMESRGPLSCDTVLKIFYQT

CRAVQHMHRQKPPIIHRDLKVENLLLSNQGTIKLCDFGSATTISHYPDYSWSAQRRALVE

EEITRNTTPMYRTPEIIDLYSNFPIGEKQDIWALGCILYLLCFRQHPFEDGAKLRIVNGK

YSIPPHDTQYTVFHSLIRAMLQVNPEERLSIAEVVHQLQEI

>hGSK3a_519

QEVAYTDIKVIGNGSFGVVYQARLAETRELVAIKKVLQDKRFKNRELQIMRKLDHCNIVR

LRYFFYSSGEKKDELYLNLVLEYVPETVYRVARHFTKAKLTIPILYVKVYMYQLFRSLAY

IHSQGVCHRDIKPQNLLVDPDTAVLKLCDFGSAKQLVRGEPNVSYICSRYYRAPELIFGA

TDYTSSIDVWSAGCVLAELLLGQPIFPGDSGVDQLVEIIKVLGTPTREQIREMNPNYTEF

KFPQIKAHPWTKVFKSRTPPEAIALCSSLLEYTPSSRLSPLEACAHSFFD

>hGSK3b_231

QEVSYTDTKVIGNGSFGVVYQAKLCDSGELVAIKKVLQDKRFKNRELQIMRKLDHCNIVR

LRYFFYSSGEKKDEVYLNLVLDYVPETVYRVARHYSRAKQTLPVIYVKLYMYQLFRSLAY

IHSFGICHRDIKPQNLLLDPDTAVLKLCDFGSAKQLVRGEPNVSYICSRYYRAPELIFGA

TDYTSSIDVWSAGCVLAELLLGQPIFPGDSGVDQLVEIIKVLGTPTREQIREMNPNYTEF

KFPQIKAHPWTKVFRPRTPPEAIALCSRLLEYTPTARLTPLEACAHSFFD

>hHCK_2294

PRESLKLEKKLGAGQFGEVWMATYNKHTKVAVKTMKPGSMSVEAFLAEANVMKTLQHDKL

VKLHAVVTKEPIYIITEFMAKGSLLDFLKSDEGSKQPLPKLIDFSAQIAEGMAFIEQRNY

IHRDLRAANILVSASLVCKIADFGLARVIEDNEYTAREGAKFPIKWTAPEAINFGSFTIK

SDVWSFGILLMEIVTYGRIPYPGMSNPEVIRALERGYRMPRPENCPEELYNIMMRCWKNR

PEERPTFEYIQSVLDDF

>hHER2_1572

KETELRKVKVLGSGAFGTVYKGIWIPDGENVKIPVAIKVLRENTSPKANKEILDEAYVMA

GVGSPYVSRLLGICLTSTVQLVTQLMPYGCLLDHVRENRGRLGSQDLLNWCMQIAKGMSY

LEDVRLVHRDLAARNVLVKSPNHVKITDFGLARLLDIDETEYHADGGKVPIKWMALESIL

RRRFTHQSDVWSYGVTVWELMTFGAKPYDGIPAREIPDLLEKGERLPQPPICTIDVYMIM

VKCWMIDSECRPRFRELVSEFSRM

>hHER4_1536

KETELKRVKVLGSGAFGTVYKGIWVPEGETVKIPVAIKILNETTGPKANVEFMDEALIMA

SMDHPHLVRLLGVCLSPTIQLVTQLMPHGCLLEYVHEHKDNIGSQLLLNWCVQIAKGMMY

LEERRLVHRDLAARNVLVKSPNHVKITDFGLARLLEGDEKEYNADGGKMPIKWMALECIH

YRKFTHQSDVWSYGVTIWELMTFGGKPYDGIPTREIPDLLEKGERLPQPPICTIDVYMVM

VKCWMIDADSRPKFKELAAEFSRM

>hHIPK2_71

MTNTYEVLEFLGRGTFGQVVKCWKRGTNEIVAIKILKNHPSYARQGQIEVSILARLSTES

ADDYNFVRAYECFQHKNHTCLVFEMLEQNLYDFLKQNKFSPLPLKYIRPVLQQVATALMK

LKSLGLIHADLKPENIMLVDPSRQPYRVKVIDFGSASHVSKAVCSTYLQSRYYRAPEIIL

GLPFCEAIDMWSLGCVIAELFLGWPLYPGASEYDQIRYISQTQGLPAEYLLSAGTKTTRF

FNRDTDSPYPLWRLKTPDDHEAETGIKSKEARKYIFNCLDDMAQVNMTTDLEGSDMLVEK

ADRREFIDLLKKMLTIDADKRITPIETLNHPFVT

>hIGF1R_934

AREKITMSRELGQGSFGMVYEGVAKGVVKDEPETRVAIKTVNEAASMRERIEFLNEASVM

KEFNCHHVVRLLGVVSQGQPTLVIMELMTRGDLKSYLRSLRPEMENNPVLAPPSLSKMIQ

MAGEIADGMAYLNANKFVHRDLAARNCMVAEDFTVKIGDFGMTRDIYETDYYRKGGKGLL

PVRWMSPESLKDGVFTTYSDVWSFGVVLWEIATLAEQPYQGLSNEQVLRFVMEGGLLDKP

DNCPDMLFELMRMCWQYNPKMRPSFLEIISSIKEE

>hIKBKB_452

TCGAWEMKERLGTGGFGNVIRWHNQETGEQIAIKQCRQELSPRNRERWCLEIQIMRRLTH

PNVVAARDVPEGMQNLAPNDLPLLAMEYCQGGDLRKYLNQFENCCGLREGAILTLLSDIA

SALRYLHENRIIHRDLKPENIVLQQGEQRLIHKIIDLGYAKELDQGSLCTSFVGTLQYLA

PELLEQQKYTVTVDYWSFGTLAFECITGFRPFLPNWQPVQWHSKVRQKSEVDIVVSEDLN

GTVKFSSSLPYPNNLNSVLAERLEKWLQLMLMWHPRQRGTDPTYGPNGCFK

>hIKBKE_278

ANYLWHTDDLLGQGATASVYKARNKKSGELVAVKVFNTTSYLRPREVQVREFEVLRKLNH

QNIVKLFAVEETGGSRQKVLVMEYCSSGSLLSVLESPENAFGLPEDEFLVVLRCVVAGMN

HLRENGIVHRDIKPGNIMRLVGEEGQSIYKLTDFGAARELDDDEKFVSVYGTEEYLHPDM

YERAVLRKPQQKAFGVTVDLWSIGVTLYHAATGSLPFIPFGGPRRNKEIMYRITTEKPAG

AIAGAQRRENGPLEWSYTLPITCQLSLGLQSQLVPILANILEVEQAKCWGFDQFFAETSD

I

>hINSR_920

SREKITLLRELGQGSFGMVYEGNARDIIKGEAETRVAVKTVNESASLRERIEFLNEASVM

KGFTCHHVVRLLGVVSKGQPTLVVMELMAHGDLKSYLRSLRPEAENNPGRPPPTLQEMIQ

MAAEIADGMAYLNAKKFVHRDLAARNCMVAHDFTVKIGDFGMTRDIYETDYYRKGGKGLL

PVRWMAPESLKDGVFTTSSDMWSFGVVLWEITSLAEQPYQGLSNEQVLKFVMDGGYLDQP

DNCPERVTDLMRMCWQFNPKMRPTFLEIVNLLKDD

>hIRAK4_908

ERPISVGGNKMGEGGFGVVYKGYVNNTTVAVKKLAAMVDITTEELKQQFDQEIKVMAKCQ

HENLVELLGFSSDGDDLCLVYVYMPNGSLLDRLSCLDGTPPLSWHMRCKIAQGAANGINF

LHENHHIHRDIKSANILLDEAFTAKISDFGLARASEKFAQTVMTSRIVGTTAYMAPEALR

GEITPKSDIYSFGVVLLEIITGLPAVDEHREPQLLLDIKEEIEDEEKTIEDYIDKKMNDA

DSTSVEAMYSVASQCLHEKKNKRPDIKKVQQLLQEM

>hITK_2233

DPSELTFVQEIGSGQFGLVHLGYWLNKDKVAIKTIREGAMSEEDFIEEAEVMMKLSHPKL

VQLYGVCLEQAPICLVFEFMEHGCLSDYLRTQRGLFAAETLLGMCLDVCEGMAYLEEACV

IHRDLAARNCLVGENQVIKVSDFGMTRFVLDDQYTSSTGTKFPVKWASPEVFSFSRYSSK

SDVWSFGVLMWEVFSEGKIPYENRSNSEVVEDISTGFRLYKPRLASTHVYQIMNHCWKER

PEDRPAFSRLLRQLAEI

>hJAK1_d1_1114

LKKDLVQGEHLGRGTRTHIYSGTLMDYKDDEGTSEEKKIKVILKVLDPSHRDISLAFFEA

ASMMRQVSHKHIVYLYGVCVRDVENIMVEEFVEGGPLDLFMHRKSDVLTTPWKFKVAKQL

ASALSYLEDKDLVHGNVCTKNLLLAREGIDSECGPFIKLSDPGIPITVLSRQECIERIPW

IAPECVEDSKNLSVAADKWSFGTTLWEICYNGEIPLKDKTLIEKERFYESRCRPVTPSCK

ELADLMTRCMNYDPNQRPFFRAIMRDINKL

>hJAK1_d2_772

EKRFLKRIRDLGEGHFGKVELCRYDPEDNTGEQVAVKSLKPESGGNHIADLKKEIEILRN

LYHENIVKYKGICTEDGGNGIKLIMEFLPSGSLKEYLPKNKNKINLKQQLKYAVQICKGM

DYLGSRQYVHRDLAARNVLVESEHQVKIGDFGLTKAIETDKEYYTVKDDRDSPVFWYAPE

CLMQSKFYIASDVWSFGVTLHELLTYCDSDSSPMALFLKMIGPTHGQMTVTRLVNTLKEG

KRLPCPPNCPDEVYQLMRKCWEFQPSNRTSFQNLIEGFEAL

>hJAK2_d1_1212

RNEDLIFNESLGQGTFTKIFKGVRREVGDYGQLHETEVLLKVLDKAHRNYSESFFEAASM

MSKLSHKHLVLNYGVCVCGDENILVQEFVKFGSLDTYLKKNKNCINILWKLEVAKQLAWA

MHFLEENTLIHGNVCAKNILLIREEDRKTGNPPFIKLSDPGISITVLPKDILQERIPWVP

PECIENPKNLNLATDKWSFGTTLWEICSGGDKPLSALDSQRKLQFYEDRHQLPAPKWAEL

ANLINNCMDYEPDFRPSFRAIIRDLNSL

>hJAK2_d2_766

EERHLKFLQQLGKGNFGSVEMCRYDPLQDNTGEVVAVKKLQHSTEEHLRDFEREIEILKS

LQHDNIVKYKGVCYSAGRRNLKLIMEYLPYGSLRDYLQKHKERIDHIKLLQYTSQICKGM

EYLGTKRYIHRDLATRNILVENENRVKIGDFGLTKVLPQDKEYYKVKEPGESPIFWYAPE

SLTESKFSVASDVWSFGVVLYELFTYIEKSKSPPAEFMRMIGNDKQGQMIVFHLIELLKN

NGRLPRPDGCPDEIYMIMTECWNNNVNQRPSFRDLALRVDQI

>hJAK3_d1_2687

IPADSLEWHENLGHGSFTKIYRGCRHEVVDGEARKTEVLLKVMDAKHKNCMESFLEAASL

MSQVSYRHLVLLHGVCMAGDSTMVQEFVHLGAIDMYLRKRGHLVPASWKLQVVKQLAYAL

NYLEDKGLPHGNVSARKVLLAREGADGSPPFIKLSDPGVSPAVLSLEMLTDRIPWVAPEC

LREAQTLSLEADKWGFGATVWEVFSGVTMPISALDPAKKLQFYEDRQQLPAPKWTELALL

IQQCMAYEPVQRPSFRAVIRDLNSL

>hJAK3_d2_778

EERHLKYISQLGKGNFGSVELCRYDPLGDNTGALVAVKQLQHSGPDQQRDFQREIQILKA

LHSDFIVKYRGVSYGPGRQSLRLVMEYLPSGCLRDFLQRHRARLDASRLLLYSSQICKGM

EYLGSRRCVHRDLAARNILVESEAHVKIADFGLAKLLPLDKDYYVVREPGQSPIFWYAPE

SLSDNIFSRQSDVWSFGVVLYELFTYCDKSCSPSAEFLRMMGCERDVPALCRLLELLEEG

QRLPAPPACPAEVHELMKLCWAPSPQDRPSFSALGPQLDML

>hJNK1_294

VLKRYQNLKPIGSGAQGIVCAAYDAILERNVAIKKLSRPFQNQTHAKRAYRELVLMKCVN

HKNIIGLLNVFTPQKSLEEFQDVYIVMELMDANLCQVIQMELDHERMSYLLYQMLCGIKH

LHSAGIIHRDLKPSNIVVKSDCTLKILDFGLARTAGTSFMMTPYVVTRYYRAPEVILGMG

YKENVDLWSVGCIMGEMVCHKILFPGRDYIDQWNKVIEQLGTPCPEFMKKLQPTVRTYVE

NRPKYAGYSFEKLFPDVLFPADSEHNKLKASQARDLLSKMLVIDASKRISVDEALQHPYI

N

>hJNK2_271

VLKRYQQLKPIGSGAQGIVCAAFDTVLGISVAVKKLSRPFQNQTHAKRAYRELVLLKCVN

HKNIISLLNVFTPQKTLEEFQDVYLVMELMDANLCQVIHMELDHERMSYLLYQMLCGIKH

LHSAGIIHRDLKPSNIVVKSDCTLKILDFGLARTACTNFMMTPYVVTRYYRAPEVILGMG

YKENVDIWSVGCIMGELVKGCVIFQGTDHIDQWNKVIEQLGTPSAEFMKKLQPTVRNYVE

NRPKYPGIKFEELFPDWIFPSESERDKIKTSQARDLLSKMLVIDPDKRISVDEALRHPYI

T

>hJNK3_261

VLKRYQNLKPIGSGAQGIVCAAYDAVLDRNVAIKKLSRPFQNQTHAKRAYRELVLMKCVN

HKNIISLLNVFTPQKTLEEFQDVYLVMELMDANLCQVIQMELDHERMSYLLYQMLCGIKH

LHSAGIIHRDLKPSNIVVKSDCTLKILDFGLARTAGTSFMMTPYVVTRYYRAPEVILGMG

YKENVDIWSVGCIMGEMVRHKILFPGRDYIDQWNKVIEQLGTPCPEFMKKLQPTVRNYVE

NRPKYAGLTFPKLFPDSLFPADSEHNKLKASQARDLLSKMLVIDPAKRISVDDALQHPYI

N

>hKIT_40

PRNRLSFGKTLGAGAFGKVVEATAYGLIKSDAAMTVAVKMLKPSAHLTEREALMSELKVL

SYLGNHMNIVNLLGACTIGGPTLVITEYCCYGDLLNFLRRKRDSFICSKQEVRIGSYIER

DVTPAIMEDDELALDLEDLLSFSYQVAKGMAFLASKNCIHRDLAARNILLTHGRITKICD

FGLARDIKNDSNYVVKGNARLPVKWMAPESIFNCVYTFESDVWSYGIFLWELFSLGSSPY

PGMPVDSKFYKMIKEGFRMLSPEHAPAEMYDIMKTCWDADPLKRPTFKQIVQLIEKQ

>hLCK_2238

PRETLKLVERLGAGQFGEVWMGYYNGHTKVAVKSLKQGSMSPDAFLAEANLMKQLQHQRL

VRLYAVVTQEPIYIITEYMENGSLVDFLKTPSGIKLTINKLLDMAAQIAEGMAFIEERNY

IHRDLRAANILVSDTLSCKIADFGLARLIEDNEYTAREGAKFPIKWTAPEAINYGTFTIK

SDVWSFGILLTEIVTHGRIPYPGMTNPEVIQNLERGYRMVRPDNCPEELYQLMRLCWKER

PEDRPTFDYLRSVLEDF

>hLIMK1_983

RPSDLIHGEVLGKGCFGQAIKVTHRETGEVMVMKELIRFDEETQRTFLKEVKVMRCLEHP

NVLKFIGVLYKDKRLNFITEYIKGGTLRGIIKSMDSQYPWSQRVSFAKDIASGMAYLHSM

NIIHRDLNSHNCLVRENKNVVVADFGLARLMVDEKTQPEGLRSLKKPDRKKRYTVVGNPY

WMAPEMINGRSYDEKVDVFSFGIVLCEIIGRVNADPDYLPRTMDFGLNVRGFLDRYCPPN

CPPSFYPITVRCCDLDPEKRPSFVKLEHWLETL

>hLIMK2_839

RPCDLIHGEVLGKGFFGQAIKVTHKATGKVMVMKELIRCDEETQKTFLTEVKVMRSLDHP

NVLKFIGVLYKDKKLNLLTEYIEGGTLKDFLRSMDPFPWQQKVRFAKGIASGMAYLHSMC

IIHRDLNSHNCLIKLDKTVVVADFGLSRLIVEERKRAPMEKATTKKRTLRKNDRKKRYTV

VGNPYWMAPEMLNGKSYDETVDIFSFGIVLCEIIGQVYADPDCLPRTLDFGLNVKLFWEK

FVPTDCPPAFFPLAAICCRLEPESRPAFSKLEDSFEAL

>hLTK_955

SPANVTLLRALGHGAFGEVYEGLVIGLPGDSSPLQVAIKTLPELCSPQDELDFLMEALII

SKFRHQNIVRCVGLSLRATPRLILLELMSGGDMKSFLRHSRPHLGQPSPLVMRDLLQLAQ

DIAQGCHYLEENHFIHRDIAARNCLLSCAGPSRVAKIGDFGMARDIYRASYYRRGDRALL

PVKWMPPEAFLEGIFTSKTDSWSFGVLLWEIFSLGYMPYPGRTNQEVLDFVVGGGRMDPP

RGCPGPVYRIMTQCWQHEPELRPSFASILERLQYC

>hLYN_2111

PRESIKLVKRLGAGQFGEVWMGYYNNSTKVAVKTLKPGTMSVQAFLEEANLMKTLQHDKL

VRLYAVVTREEPIYIITEYMAKGSLLDFLKSDEGGKVLLPKLIDFSAQIAEGMAYIERKN

YIHRDLRAANVLVSESLMCKIADFGLARVIEDNEYTAREGAKFPIKWTAPEAINFGCFTI

KSDVWSFGILLYEIVTYGKIPYPGRTNADVMTALSQGYRMPRVENCPDELYDIMKMCWKE

KAEERPTFDYLQSVLDDF

>hMAP3K10_1243

PFHELQLEEIIGVGGFGKVYRALWRGEEVAVKAARLDPEKDPAVTAEQVCQEARLFGALQ

HPNIIALRGACLNPPHLCLVMEYARGGALSRVLAGRRVPPHVLVNWAVQVARGMNYLHND

APVPIIHRDLKSINILILEAIENHNLADTVLKITDFGLAREWHKTTKMSAAGTYAWMAPE

VIRLSLFSKSSDVWSFGVLLWELLTGEVPYREIDALAVAYGVAMNKLTLPIPSTCPEPFA

RLLEECWDPDPHGRPDFGSILKRLEVI

>hMAP3K11_1236

SFQELRLEEVIGIGGFGKVYRGSWRGELVAVKAARQDPDEDISVTAESVRQEARLFAMLA

HPNIIALKAVCLEEPNLCLVMEYAAGGPLSRALAGRRVPPHVLVNWAVQIARGMHYLHCE

ALVPVIHRDLKSNNILLLQPIESDDMEHKTLKITDFGLAREWHKTTQMSAAGTYAWMAPE

VIKASTFSKGSDVWSFGVLLWELLTGEVPYRGIDCLAVAYGVAVNKLTLPIPSTCPEPFA

QLMADCWAQDPHRRPDFASILQQLEAL

>hMAP3K1_1051

EDTEWLKGQQIGLGAFSSCYQAQDVGTGTLMAVKQVTYVRNTSSEQEEVVEALREEIRMM

SHLNHPNIIRMLGATCEKSNYNLFIEWMAGGSVAHLLSKYGAFKESVVINYTEQLLRGLS

YLHENQIIHRDVKGANLLIDSTGQRLRIADFGAAARLASKGTGAGEFQGQLLGTIAFMAP

EVLRGQQYGRSCDVWSVGCAIIEMACAKPPWNAEKHSNHLALIFKIASATTAPSIPSHLS

PGLRDVALRCLELQPQDRPPSRELLKHPVFR

>hMAP3K5_1466

EYDENGDRVVLGKGTYGIVYAGRDLSNQVRIAIKEIPERDSRYSQPLHEEIALHKHLKHK

NIVQYLGSFSENGFIKIFMEQVPGGSLSALLRSKWGPLKDNEQTIGFYTKQILEGLKYLH

DNQIVHRDIKGDNVLINTYSGVLKISDFGTSKRLAGINPCTETFTGTLQYMAPEIIDKGP

RGYGKAADIWSLGCTIIEMATGKPPFYELGEPQAAMFKVGMFKVHPEIPESMSAEAKAFI

LKCFEPDPDKRACANDLLVDEFLK

>hMAP3K8_2389

LTYRNIGSDFIPRGAFGKVYLAQDIKTKKRMACKLIPVDQFKPSDVEIQACFRHENIAEL

YGAVLWGETVHLFMEAGEGGSVLEKLESCGPMREFEIIWVTKHVLKGLDFLHSKKVIHHD

IKPSNIVFMSTKAVLVDFGLSVQMTEDVYFPKDLRGTEIYMSPEVILCRGHSTKADIYSL

GATLIHMQTGTPPWVKRYPRSAYPSYLYIIHKQAPPLEDIADDCSPGMRELIEASLERNP

NHRPRAADLLKHEALN

>hMAP3K9_1226

DFAELTLEEIIGIGGFGKVYRAFWIGDEVAVKAARHDPDEDISQTIENVRQEAKLFAMLK

HPNIIALRGVCLKEPNLCLVMEFARGGPLNRVLSGKRIPPDILVNWAVQIARGMNYLHDE

AIVPIIHRDLKSSNILILQKVENGDLSNKILKITDFGLAREWHRTTKMSAAGTYAWMAPE

VIRASMFSKGSDVWSYGVLLWELLTGEVPFRGIDGLAVAYGVAMNKLALPIPSTCPEPFA

KLMEDCWNPDPHSRPSFTNILDQLTTI

>hMAP4K2_1661

PRDRFELLQRVGAGTYGDVYKARDTVTSELAAVKIVKLDPGDDISSLQQEITILRECRHP

NVVAYIGSYLRNDRLWICMEFCGGGSLQEIYHATGPLEERQIAYVCRERLKGLHHLHSQG

KIHRDIKGANLLLTLQGDVKLADFGVSGELTASVAKRRSFIGTPYWMAPEVAAVERKGGY

NELCDVWALGITAIELGELQPPLFHLHPMRALMLMSKSSFQPPKLRDKTRWTQNFHHFLK

LALTKNPKKRPTAEKLLQHPFTT

>hMAP4K4_1081

PAGIFELVEVVGNGTYGQVYKGRHVKTGQLAAIKVMDVTEDEEEEIKLEINMLKKYSHHR

NIATYYGAFIKKSPPGHDDQLWLVMEFCGAGSITDLVKNTKGNTLKEDWIAYISREILRG

LAHLHIHHVIHRDIKGQNVLLTENAEVKLVDFGVSAQLDRTVGRRNTFIGTPYWMAPEVI

ACDENPDATYDYRSDLWSCGITAIEMAEGAPPLCDMHPMRALFLIPRNPPPRLKSKKWSK

KFFSFIEGCLVKNYMQRPSTEQLLKHPFIR

>hMAPKAPK3_1328

TDDYQLSKQVLGLGVNGKVLECFHRRTGQKCALKLLYDSPKARQEVDHHWQASGGPHIVC

ILDVYENMHHGKRCLLIIMECMEGGELFSRIQERGDQAFTEREAAEIMRDIGTAIQFLHS

HNIAHRDVKPENLLYTSKEKDAVLKLTDFGFAKETTQNALQTPCYTPYYVAPEVLGPEKY

DKSCDMWSLGVIMYILLCGFPPFYSNTGQAISPGMKRRIRLGQYGFPNPEWSEVSEDAKQ

LIRLLLKTDPTERLTITQFMNHPWIN

>hMAPKAPK5_580

EEYSINWTQKLGAGISGPVRVCVKKSTQERFALKILLDRPKARNEVRLHMMCATHPNIVQ

IIEVFANSVQFPHESSPRARLLIVMEMMEGGELFHRISQHRHFTEKQASQVTKQIALALR

HCHLLNIAHRDLKPENLLFKDNSLDAPVKLCDFGFAKIDQGDLMTPQFTPYYVAPQVLEA

QRRHQKEKSGIIPTSPTPYTYNKSCDLWSLGVIIYVMLCGYPPFYSKHHSRTIPKDMRRK

IMTGSFEFPEEEWSQISEMAKDVVRKLLKVKPEERLTIEGVLDHPWLN

>hMARK2_2180

HIGNYRLLKTIGKGNFAKVKLARHILTGKEVAVKIIDKTQLNSSSLQKLFREVRIMKVLN

HPNIVKLFEVIETEKTLYLVMEYASGGEVFDYLVAHGRMKEKEARAKFRQIVSAVQYCHQ

KFIVHRDLKAENLLLDADMNIKIADFGFSNEFTFGNKLDTFCGSPPYAAPELFQGKKYDG

PEVDVWSLGVILYTLVSGSLPFDGQNLKELRERVLRGKYRIPFYMSTDCENLLKKFLILN

PSKRGTLEQIMKDRWMN

>hMARK3_825

HIGNYRLLKTIGKGNFAKVKLARHILTGREVAIKIIDKTQLNPTSLQKLFREVRIMKILN

HPNIVKLFEVIETEKTLYLIMEYASGGEVFDYLVAHGRMKEKEARSKFRQGCQAGQTIKV

QVSFDLLSLMFTFIVSAVQYCHQKRIVHRDLKAENLLLDADMNIKIADFGFSNEFTVGGK

LDTFCGSPPYAAPELFQGKKYDGPEVDVWSLGVILYTLVSGSLPFDGQNLKELRERVLRG

KYRIPFYMSTDCENLLKRFLVLNPIKRGTLEQIMKDRWIN

>hMEK1_307

KDDDFEKISELGAGNGGVVFKVSHKPSGLVMARKLIHLEIKPAIRNQIIRELQVLHECNS

PYIVGFYGAFYSDGEISICMEHMDGGSLDQVLKKAGRIPEQILGKVSIAVIKGLTYLREK

HKIMHRDVKPSNILVNSRGEIKLCDFGVSGQLIDSMANSFVGTRSYMSPERLQGTHYSVQ

SDIWSMGLSLVEMAVGRYPIPPPDAKELELMFGCQVEGDAAETPPRPRTPGRPLSSYGMD

SRPPMAIFELLDYIVNEPPPKLPSGVFSLEFQDFVNKCLIKNPAERADLKQLMVHAFIK

>hMEK2_226

KDDDFERISELGAGNGGVVTKVQHRPSGLIMARKLIHLEIKPAIRNQIIRELQVLHECNS

PYIVGFYGAFYSDGEISICMEHMDGGSLDQVLKEAKRIPEEILGKVSIAVLRGLAYLREK

HQIMHRDVKPSNILVNSRGEIKLCDFGVSGQLIDSMANSFVGTRSYMAPERLQGTHYSVQ

SDIWSMGLSLVELAVGRYPIPPPDAKELEAIFGRPVVDGEEGEPHSISPRPRPPGRPVSG

HGMDSRPAMAIFELLDYIVNEPPPKLPNGVFTPDFQEFVNKCLIKNPAERADLKMLTNHT

FIK

>hMERTK_945

DRNLLILGKILGEGEFGSVMEGNLKQEDGTSLKVAVKTMKLDNSSHREIEEFLSEAACMK

DFSHPNVIRLLGVCIEMSSQGIPKPMVILPFMKYGDLHTYLLYSRLETGPKHIPLQTLLK

FMVDIALGMEYLSNRNFLHRDLAARNCMLRDDMTVCVADFGLSKKIYSGDYYRQGRIAKM

PVKWIAIESLADRVYTSKSDVWAFGVTMWEIRTRGMTPYPGVQNHEMYDYLLHGHRLKQP

EDCLDELYEIMYSCWRTDPLDRPTFSVLRLQLEKL

>hMET_1272

SSLIVHFNEVIGRGHFGCVYHGTLLDNDGKKIHCAVKSLNRITDIGEVSQFLTEGIIMKD

FSHPNVLSLLGICLRSEGSPLVVLPYMKHGDLRNFIRNETHNPTVKDLIGFGLQVAKGMK

YLASKKFVHRDLAARNCMLDEKFTVKVADFGLARDMYDKEYYSVHNKTGAKLPVKWMALE

SLQTQKFTTKSDVWSFGVVLWELMTRGAPPYPDVNTFDITVYLLQGRRLLQPEYCPDPLY

EVMLKCWHPKAEMRPSFSELVSRISAI

>hMK2_1275

IDDYKVTSQVLGLGINGKVLQIFNKRTQEKFALKMLQDCPKARREVELHWRASQCPHIVR

IVDVYENLYAGRKCLLIVMECLDGGELFSRIQDRGDQAFTEREASEIMKSIGEAIQYLHS

INIAHRDVKPENLLYTSKRPNAILKLTDFGFAKETTSHNSLTTPCYTPYYVAPEVLGPEK

YDKSCDMWSLGVIMYILLCGYPPFYSNHGLAISPGMKTRIRMGQYEFPNPEWSEVSEEVK

MLIRNLLKTEPTQRMTITEFMNHPWIM

>hMKK6_1284

KADDLEPIMELGRGAYGVVEKMRHVPSGQIMAVKRIRATVNSQEQKRLLMDLDISMRTVD

CPFTVTFYGALFREGDVWICMELMDTSLDKFYKQVIDKGQTIPEDILGKIAVSIVKALEH

LHSKLSVIHRDVKPSNVLINALGQVKMCDFGISGYLVDSVAKTIDAGCKPYMAPERINPE

LNQKGYSVKSDIWSLGITMIELAILRFPYDSWGTPFQQLKQVVEEPSPQLPADKFSAEFV

DFTSQCLKKNSKERPTYPELMQHPFFT

>hMKNK1_94

EDMYKLTSELLGEGAYAKVQGAVSLQNGKEYAVKIIEKQAGHSRSRVFREVETLYQCQGN

KNILELIEFFEDDTRFYLVFEKLQGGSILAHIQKQKHFNEREASRVVRDVAAALDFLHTK

DKVSLCHLGWSAMAPSGLTAAPTSLGSSDPPTSASQVAGTTGIAHRDLKPENILCESPEK

VSPVKICDFDLGSGMKLNNSCTPITTPELTTPCGSAEYMAPEVVEVFTDQATFYDKRCDL

WSLGVVLYIMLSGYPPFVGHCGADCGWDRGEVCRVCQNKLFESIQEGKYEFPDKDWAHIS

SEAKDLISKLLVRDAKQRLSAAQVLQHPWVQ

>hMSK1_d1_1076

GIENFELLKVLGTGAYGKVFLVRKISGHDTGKLYAMKVLKKATIVQKAKTTEHTRTERQV

LEHIRQSPFLVTLHYAFQTETKLHLILDYINGGELFTHLSQRERFTEHEVQIYVGEIVLA

LEHLHKLGIIYRDIKLENILLDSNGHVVLTDFGLSKEFVADETERAYSFCGTIEYMAPDI

VRGGDSGHDKAVDWWSLGVLMYELLTGASPFTVDGEKNSQAEISRRILKSEPPYPQEMSA

LAKDLIQRLLMKDPKKRLGCGPRDADEIKE

>hMSK1_d2_1253

HYDLDLKDKPLGEGSFSICRKCVHKKSNQAFAVKIISKRMEANTQKEITALKLCEGHPNI

VKLHEVFHDQLHTFLVMELLNGGELFERIKKKKHFSETEASYIMRKLVSAVSHMHDVGVV

HRDLKPENLLFTDENDNLEIKIIDFGFARLKPPDNQPLKTPCFTLHYAAPELLNQNGYDE

SCDLWSLGVILYTMLSGQVPFQSHDRSLTCTSAVEIMKKIKKGDFSFEGEAWKNVSQEAK

DLIQGLLTVDPNKRLKMSGLRYNEWLQ

>hMSK2_d1_1120

SVENFELLKVLGTGAYGKVFLVRKAGGHDAGKLYAMKVLRKAALVQRAKTQEHTRTERSV

LELVRQAPFLVTLHYAFQTDAKLHLILDYVSGGEMFTHLYQRQYFKEAEVRVYGGEIVLA

LEHLHKLGIIYRDLKLENVLLDSEGHIVLTDFGLSKEFLTEEKERTFSFCGTIEYMAPEI

IRSKTGHGKAVDWWSLGILLFELLTGASPFTLEGERNTQAEVSRRILKCSPPFPPRIGPV

AQDLLQRLLCKDPKKRLGAGPQGAQEVRN

>hMSK2_d2_1132

QYELDLREPALGQGSFSVCRRCRQRQSGQEFAVKILSRRLEANTQREVAALRLCQSHPNV

VNLHEVHHDQLHTYLVLELLRGGELLEHIRKKRHFSESEASQILRSLVSAVSFMHEEAGV

VHRDLKPENILYADDTPGAPVKIIDFGFARLRPQSPGVPMQTPCFTLQYAAPELLAQQGY

DESCDLWSLGVILYMMLSGQVPFQGASGQGGQSQAAEIMCKIREGRFSLDGEAWQGVSEE

AKELVRGLLTVDPAKRLKLEGLRGSSWLQ

>hMST1R_1266

ERVVTHSDRVIGKGHFGVVYHGEYIDQAQNRIQCAIKSLSRITEMQQVEAFLREGLLMRG

LNHPNVLALIGIMLPPEGLPHVLLPYMCHGDLLQFIRSPQRNPTVKDLISFGLQVARGME

YLAEQKFVHRDLAARNCMLDESFTVKVADFGLARDILDREYYSVQQHRHARLPVKWMALE

SLQTYRFTTKSDVWSFGVLLWELLTRGAPPYRHIDPFDLTHFLAQGRRLPQPEYCPDSLY

QVMQQCWEADPAVRPTFRVLVGEVEQI

>hMYLK_1825

VSDFYDIEERLGSGKFGQVFRLVEKKTRKVWAGKFFKAYSAKEKENIRQEISIMNCLHHP

KLVQCVDAFEEKANIVMVLEIVSGGELFERIIDEDFELTERECIKYMRQISEGVEYIHKQ

GIVHLDLKPENIMCVNKTGTRIKLIDFGLARRLENAGSLKVLFGTPEFVAPEVINYEPIG

YATDMWSIGVICYILVSGLSPFMGDNDNETLANVTSATWDFDDEAFDEISDDAKDFISNL

LKKDMKNRLDCTQCLQHPWLM

>hNEK2_1140

RAEDYEVLYTIGTGSYGRCQKIRRKSDGKILVWKELDYGSMTEAEKQMLVSEVNLLRELK

HPNIVRYYDRIIDRTNTTLYIVMEYCEGGDLASVITKGTKERQYLDEEFVLRVMTQLTLA

LKECHRRSDGGHTVLHRDLKPANVFLDGKQNVKLGDFGLARILNHDTSFAKTFVGTPYYM

SPEQMNRMSYNEKSDIWSLGCLLYELCALMPPFTAFSQKELAGKIREGKFRRIPYRYSDE

LNEIITRMLNLKDYHRPSVEEILENPLIA

>hNEK6_1308

SLADFQIEKKIGRGQFSEVYKATCLLDRKTVALKKVQIFEMMDAKARQDCVKEIGLLKQL

NHPNIIKYLDSFIEDNELNIVLELADAGDLSQMIKYFKKQKRLIPERTVWKYFVQLCSAV

EHMHSRRVMHRDIKPANVFITATGVVKLGDLGLGRFFSSETTAAHSLVGTPYYMSPERIH

ENGYNFKSDIWSLGCLLYEMAALQSPFYGDKMNLFSLCQKIEQCDYPPLPGEHYSEKLRE

LVSMCICPDPHQRPDIGYVHQVAKQM

>hNTRK1_826

KRRDIVLKWELGEGAFGKVFLAECHNLLPEQDKMLVAVKALKEASESARQDFQREAELLT

MLQHQHIVRFFGVCTEGRPLLMVFEYMRHGDLNRFLRSHGPDAKLLAGGEDVAPGPLGLG

QLLAVASQVAAGMVYLAGLHFVHRDLATRNCLVGQGLVVKIGDFGMSRDIYSTDYYRVGG

RTMLPIRWMPPESILYRKFTTESDVWSFGVVLWEIFTYGKQPWYQLSNTEAIDCITQGRE

LERPRACPPEVYAIMRGCWQREPQQRHSIKDVHARLQAL

>hNTRK2_860

KRHNIVLKRELGEGAFGKVFLAECYNLCPEQDKILVAVKTLKDASDNARKDFHREAELLT

NLQHEHIVKFYGVCVEGDPLIMVFEYMKHGDLNKFLRAHGPDAVLMAEGNPPTELTQSQM

LHIAQQIAAGMVYLASQHFVHRDLATRNCLVGENLLVKIGDFGMSRDVYSTDYYRVGGHT

MLPIRWMPPESIMYRKFTTESDVWSLGVVLWEIFTYGKQPWYQLSNNEVIECITQGRVLQ

RPRTCPQEVYELMLGCWQREPHMRKNIKGIHTLLQNL

>hNUAK1_2187

LKHRYELQETLGKGTYGKVKRATERFSGRVVAIKSIRKDKIKDEQDMVHIRREIEIMSSL

NHPHIISIYEVFENKDKIVIIMEYASKGELYDYISERRRLSERETRHFFRQIVSAVHYCH

KNGVVHRDLKLENILLDDNCNIKIADFGLSNLYQKDKFLQTFCGSPLYASPEIVNGRPYR

GPEVDSWALGVLLYTLVYGTMPFDGFDHKNLIRQISSGEYREPTQPSDARGLIRWMLMVN

PDRRATIEDIANHWWVN

>hPAK3_2254

PKKKYTRFEKIGQGASGTVYTALDIATGQEVAIKQMNLQQQPKKELIINEILVMRENKNP

NIVNYLDSYLVGDELWVVMEYLAGGSLTDVVTETCMDEGQIAAVCRECLQALDFLHSNQV

IHRDIKSDNILLGMDGSVKLTDFGFCAQITPEQSKRSTMVGTPYWMAPEVVTRKAYGPKV

DIWSLGIMAIEMVEGEPPYLNENPLRALYLIATNGTPELQNPERLSAVFRDFLNRCLEMD

VDRRGSAKELLQHPFLK

>hPAK4_2300

PRSYLDNFIKIGEGSTGIVCIATVRSSGKLVAVKKMDLRKQQRRELLFNEVVIMRDYQHE

NVVEMYNSYLVGDELWVVMEFLEGGALTDIVTHTRMNEEQIAAVCLAVLQALSVLHAQGV

IHRDIKSDSILLTHDGRVKLSDFGFCAQVSKEVPRRKSLVGTPYWMAPELISRLPYGPEV

DIWSLGIMVIEMVDGEPPYFNEPPLKAMKMIRDNLPPRLKNLHKVSPSLKGFLDRLLVRD

PAQRATAAELLKHPFLA

>hPAK6_2290

PRLLLDSYVKIGEGSTGIVCLAREKHSGRQVAVKMMDLRKQQRRELLFNEVVIMRDYQHF

NVVEMYKSYLVGEELWVLMEFLQGGALTDIVSQVRLNEEQIATVCEAVLQALAYLHAQGV

IHRDIKSDSILLTLDGRVKLSDFGFCAQISKDVPKRKSLVGTPYWMAPEVISRSLYATEV

DIWSLGIMVIEMVDGEPPYFSDSPVQAMKRLRDSPPPKLKNSHKVSPVLRDFLERMLVRD

PQERATAQELLDHPFLL

>hPDGFRa_27

PRDGLVLGRVLGSGAFGKVVEGTAYGLSRSQPVMKVAVKMLKPTARSSEKQALMSELKIM

THLGPHLNIVNLLGACTKSGPIYIITEYCFYGDLVNYLHKNRDSFLSHHPEYKKKSMLDS

EVKNLLSDDNSEGLTLLDLLSFTYQVARGMEFLASKNCVHRDLAARNVLLAQGKIVKICD

FGLARDIMHDSNYVSKGSTFLPVKWMAPESIFDNLYTTLSDVWSYGILLWEIFSLGGTPY

PGMMVDSTFYNKIKSGYRMAKPDHATSEVYEIMVKCWNSEPEKRPSFYHLSEIVENL

>hPDGFRb_23

PRDQLVLGRTLGSGAFGQVVEATAHGLSHSQATMKVAVKMLKSTARSSEKQALMSELKIM

SHLGPHLNVVNLLGACTKGGPIYIITEYCRYGDLVDYLHRNKHTFLQHHSDDNYVPSAPE

RTCRATLINESPVLSYMDLVGFSYQVANGMEFLASKNCVHRDLAARNVLICEGKLVKICD

FGLARDIMRDSNYISKGSTFLPLKWMAPESIFNSLYTTLSDVWSFGILLWEIFTLGGTPY

PELPMNEQFYNAIKRGYRMAQPAHASDEIYEIMQKCWEEKFEIRPPFSQLVLLLERL

>hPDPK1_1891

RPEDFKFGKILGEGSFSTVVLARELATSREYAIKILEKRHIIKENKVPYVTRERDVMSRL

DHPFFVKLYFTFQDDEKLYFGLSYAKNGELLKYIRKIGSFDETCTRFYTAEIVSALEYLH

GKGIIHRDLKPENILLNEDMHIQITDFGTAKVLSPESKQARANSFVGTAQYVSPELLTEK

SACKSSDLWALGCIIYQLVAGLPPFRAGNEYLIFQKIIKLEYDFPEKFFPKARDLVEKLL

VLDATKRLGCEEMEGYGPLK

>hPHKG1_963

FYENYEPKEILGRGVSSVVRRCIHKPTSQEYAVKVIDVTGGGSFSPEEVRELREATLKEV

DILRKVSGHPNIIQLKDTYETNTFFFLVFDLMKRGELFDYLTEKVTLSEKETRKIMRALL

EVICTLHKLNIVHRDLKPENILLDDNMNIKLTDFGFSCQLEPGERLREVCGTPSYLAPEI

IECSMNEDHPGYGKEVDMWSTGVIMYTLLAGSPPFWHRKQMLMLRMIMSGNYQFGSPEWD

DYSDTVKDLVSRFLVVQPQNRYTAEEALAHPFFQ

>hPHKG2_980

FYQKYDPKDVIGRGVSSVVRRCVHRATGHEFAVKIMEVTAERLSPEQLEEVREATRRETH

ILRQVAGHPHIITLIDSYESSSFMFLVFDLMRKGELFDYLTEKVALSEKETRSIMRSLLE

AVSFLHANNIVHRDLKPENILLDDNMQIRLSDFGFSCHLEPGEKLRELCGTPGYLAPEIL

KCSMDETHPGYGKEVDLWACGVILFTLLAGSPPFWHRRQILMLRMIMEGQYQFSSPEWDD

RSSTVKDLISRLLQVDPEARLTAEQALQHPFFE

>hPIM1_2146

LESQYQVGPLLGSGGFGSVYSGIRVSDNLPVAIKHVEKDRISDWGELPNGTRVPMEVVLL

KKVSSGFSGVIRLLDWFERPDSFVLILERPEPVQDLFDFITERGALQEELARSFFWQVLE

AVRHCHNCGVLHRDIKDENILIDLNRGELKLIDFGSGALLKDTVYTDFDGTRVYSPPEWI

RYHRYHGRSAAVWSLGILLYDMVCGDIPFEHDEEIIRGQVFFRQRVSSECQHLIRWCLAL

RPSDRPTFEEIQNHPWMQ

>hPIM2_1922

FEAEYRLGPLLGKGGFGTVFAGHRLTDRLQVAIKVIPRNRVLGWSPLSDSVTCPLEVALL

WKVGAGGGHPGVIRLLDWFETQEGFMLVLERPLPAQDLFDYITEKGPLGEGPSRCFFGQV

VAAIQHCHSRGVVHRDIKDENILIDLRRGCAKLIDFGSGALLHDEPYTDFDGTRVYSPPE

WISRHQYHALPATVWSLGILLYDMVCGDIPFERDQEILEAELHFPAHVSPDCCALIRRCL

APKPSSRPSLEEILLDPWMQ

>hPIM3_2057

FEKAYQVGAVLGSGGFGTVYAGSRIADGLPVAVKHVVKERVTEWGSLGGATVPLEVVLLR

KVGAAGGARGVIRLLDWFERPDGFLLVLERPEPAQDLFDFITERGALDEPLARRFFAQVL

AAVRHCHSCGVVHRDIKDENLLVDLRSGELKLIDFGSGALLKDTVYTDFDGTRVYSPPEW

IRYHRYHGRSATVWSLGVLLYDMVCGDIPFEQDEEILRGRLLFRRRVSPECQQLIRWCLS

LRPSERPSLDQIAAHPWML

>hPKAa_2422

HLDQFERIKTLGTGSFGRVMLVKHKETGNHYAMKILDKQKVVKLKQIEHTLNEKRILQAV

NFPFLVKLEFSFKDNSNLYMVMEYVPGGEMFSHLRRIGRFSEPHARFYAAQIVLTFEYLH

SLDLIYRDLKPENLLIDQQGYIQVTDFGFAKRVKGRTWTLCGTPEYLAPEIILSKGYNKA

VDWWALGVLIYEMAAGYPPFFADQPIQIYEKIVSGKVRFPSHFSSDLKDLLRNLLQVDLT

KRFGNLKNGVNDIKN

>hPKAb_2410

GLEDFERKKTLGTGSFGRVMLVKHKATEQYYAMKILDKQKVVKLKQIEHTLNEKRILQAV

NFPFLVRLEYAFKDNSNLYMVMEYVPGGEMFSHLRRIGRFSEPHARFYAAQIVLTFEYLH

SLDLIYRDLKPENLLIDHQGYIQVTDFGFAKRVKGRTWTLCGTPEYLAPEIILSKGYNKA

VDWWALGVLIYEMAAGYPPFFADQPIQIYEKIVSGKVRFPSHFSSDLKDLLRNLLQVDLT

KRFGNLKNGVSDIKT

>hPKAg_2407

SSDQFERLRTLGMGSFGRVMLVRHQETGGHYAMKILNKQKVVKMKQVEHILNEKRILQAI

DFPFLVKLQFSFKDNSYLYLVMEYVPGGEMFSRLQRVGRFSEPHACFYAAQVVLAVQYLH

SLDLIHRDLKPENLLIDQQGYLQVTDFGFAKRVKGRTWTLCGTPEYLAPEIILSKGYNKA

VDWWALGVLIYEMAVGFPPFYADQPIQIYEKIVSGRVRFPSKLSSDLKHLLRSLLQVDLT

KRFGNLRNGVGDIKN

>hPKCa_1986

KLTDFNFLMVLGKGSFGKVMLADRKGTEELYAIKILKKDVVIQDDDVECTMVEKRVLALL

DKPPFLTQLHSCFQTVDRLYFVMEYVNGGDLMYHIQQVGKFKEPQAVFYAAEISIGLFFL

HKRGIIYRDLKLDNVMLDSEGHIKIADFGMCKEHMMDGVTTRTFCGTPDYIAPEIIAYQP

YGKSVDWWAYGVLLYEMLAGQPPFDGEDEDELFQSIMEHNVSYPKSLSKEAVSICKGLMT

KHPAKRLGCGPEGERDVRE

>hPKCb1_2040

KLTDFNFLMVLGKGSFGKVMLSERKGTDELYAVKILKKDVVIQDDDVECTMVEKRVLALP

GKPPFLTQLHSCFQTMDRLYFVMEYVNGGDLMYHIQQVGRFKEPHAVFYAAEIAIGLFFL

QSKGIIYRDLKLDNVMLDSEGHIKIADFGMCKENIWDGVTTKTFCGTPDYIAPEIIAYQP

YGKSVDWWAFGVLLYEMLAGQAPFEGEDEDELFQSIMEHNVAYPKSMSKEAVAICKGLMT

KHPGKRLGCGPEGERDIKE

>hPKCd_2048

NINNFIFHKVLGKGSFGKVLLGELKGRGEYSAIKALKKDVVLIDDDVECTMVEKRVLTLA

AENPFLTHLICTFQTKDHLFFVMEFLNGGDLMYHIQDKGRFELYRATFYAAEIMCGLQFL

HSKGIIYRDLKLDNVLLDRDGHIKIADFGMCKENIFGESRASTFCGTPDYIAPEILQGLK

YTFSVDWWSFGVLLYEMLIGQSPFHGDDEDELFESIRVDTPHYPRWITKESKDILEKLFE

REPTKRLGMTGNIKIHPFF

>hPKCe_1997

GLDEFNFIKVLGKGSFGKVMLAELKGKDEVYAVKVLKKDVILQDDDVDCTMTEKRILALA

RKHPYLTQLYCCFQTKDRLFFVMEYVNGGDLMFQIQRSRKFDEPRSRFYAAEVTSALMFL

HQHGVIYRDLKLDNILLDAEGHCKLADFGMCKEGILNGVTTTTFCGTPDYIAPEILQELE

YGPSVDWWALGVLMYEMMAGQPPFEADNEDDLFESILHDDVLYPVWLSKEAVSILKAFMT

KNPHKRLGCVASQNGEDAI

>hPKCg_1533

HISDFSFLMVLGKGSFGKVMLAERRGSDELYAIKILKKDVIVQDDDVDCTLVEKRVLALG

GRGPGGRPHFLTQLHSTFQTPDRLYFVMEYVTGGDLMYHIQQLGKFKEPHAAFYAAEIAI

GLFFLHNQGIIYRDLKLDNVMLDAEGHIKITDFGMCKENVFPGTTTRTFCGTPDYIAPEI

IAYQPYGKSVDWWSFGVLLYEMLAGQPPFDGEDEEELFQAIMEQTVTYPKSLSREAVAIC

KGFLTKHPGKRLGSGPDGEPTIRA

>hPKCh_2002

GIDNFEFIRVLGKGSFGKVMLARVKETGDLYAVKVLKKDVILLDDDVECTMTEKRILSLA

RNHPFLTQLFCCFQTPDRLFFVMEFVNGGDLMFHIQKSRRFDEARARFYAAEIISALMFL

HDKGIIYRDLKLDNVLLDHEGHCKLADFGMCKEGICNGVTTATFCGTPDYIAPEILQEML

YGPAVDWWAMGVLLYEMLCGHAPFEAENEDDLFEAILNDEVVYPTWLHEDATGILKSFMT

KNPTMRLGSLTQGGEHAIL

>hPKCi_1189

GLQDFDLLRVIGRGSYAKVLLVRLKKTDRIYAMKVVKKELVNDDEDIDWVQTEKHVFEQA

SNHPFLVGLHSCFQTESRLFFVIEYVNGGDLMFHMQRQRKLPEEHARFYSAEISLALNYL

HERGIIYRDLKLDNVLLDSEGHIKLTDYGMCKEGLRPGDTTSTFCGTPNYIAPEILRGED

YGFSVDWWALGVLMFEMMAGRSPFDIVGSSDNPDQNTEDYLFQVILEKQIRIPRSLSVKA

ASVLKSFLNKDPKERLGCHPQTGFADIQ

>hPKCt_2008

KIEDFILHKMLGKGSFGKVFLAEFKKTNQFFAIKALKKDVVLMDDDVECTMVEKRVLSLA

WEHPFLTHMFCTFQTKENLFFVMEYLNGGDLMYHIQSCHKFDLSRATFYAAEIILGLQFL

HSKGIVYRDLKLDNILLDKDGHIKIADFGMCKENMLGDAKTNTFCGTPDYIAPEILLGQK

YNHSVDWWSFGVLLYEMLIGQSPFHGQDEEELFHSIRMDNPFYPRWLEKEAKDLLVKLFV

REPEKRLGVRGDIRQHPLF

>hPKCz_1323

GLQDFDLIRVIGRGSYAKVLLVRLKKNDQIYAMKVVKKELVHDDEDIDWVQTEKHVFEQA

SSNPFLVGLHSCFQTTSRLFLVIEYVNGGDLMFHMQRQRKLPEEHARFYAAEICIALNFL

HERGIIYRDLKLDNVLLDADGHIKLTDYGMCKEGLGPGDTTSTFCGTPNYIAPEILRGEE

YGFSVDWWALGVLMFEMMAGRSPFDIITDNPDMNTEDYLFQVILEKPIRIPRFLSVKASH

VLKGFLNKDPKERLGCRPQTGFSDIK

>hPKD1_1751

TVYQIFPDEVLGSGQFGIVYGGKHRKTGRDVAIKIIDKLRFPTKQESQLRNEVAILQNLH

HPGVVNLECMFETPERVFVVMEKLHGDMLEMILSSEKGRLPEHITKFLITQILVALRHLH

FKNIVHCDLKPENVLLASADPFPQVKLCDFGFARIIGEKSFRRSVVGTPAYLAPEVLRNK

GYNRSLDMWSVGVIIYVSLSGTFPFNEDEDIHDQIQNAAFMYPPNPWKEISHEAIDLINN

LLQVKMRKRYSVDKTLSHPWLQ

>hPKD2_1746

TVYQIFPDEVLGSGQFGVVYGGKHRKTGRDVAVKVIDKLRFPTKQESQLRNEVAILQSLR

HPGIVNLECMFETPEKVFVVMEKLHGDMLEMILSSEKGRLPERLTKFLITQILVALRHLH

FKNIVHCDLKPENVLLASADPFPQVKLCDFGFARIIGEKSFRRSVVGTPAYLAPEVLLNQ

GYNRSLDMWSVGVIMYVSLSGTFPFNEDEDINDQIQNAAFMYPASPWSHISAGAIDLINN

LLQVKMRKRYSVDKSLSHPWLQ

>hPKD3_1754

TVYQIFADEVLGSGQFGIVYGGKHRKTGRDVAIKVIDKMRFPTKQESQLRNEVAILQNLH

HPGIVNLECMFETPERVFVVMEKLHGDMLEMILSSEKSRLPERITKFMVTQILVALRNLH

FKNIVHCDLKPENVLLASAEPFPQVKLCDFGFARIIGEKSFRRSVVGTPAYLAPEVLRSK

GYNRSLDMWSVGVIIYVSLSGTFPFNEDEDINDQIQNAAFMYPPNPWREISGEAIDLINN

LLQVKMRKRYSVDKSLSHPWLQ

>hPKN1_1882

TLEDFKFLAVLGRGHFGKVLLSEFRPSGELFAIKALKKGDIVARDEVESLMCEKRILAAV

TSAGHPFLVNLFGCFQTPEHVCFVMEYSAGGDLMLHIHSDVFSEPRAIFYSACVVLGLQF

LHEHKIVYRDLKLDNLLLDTEGYVKIADFGLCKEGMGYGDRTSTFCGTPEFLAPEVLTDT

SYTRAVDWWGLGVLLYEMLVGESPFPGDDEEEVFDSIVNDEVRYPRFLSAEAIGIMRRLL

RRNPERRLGSSERDAEDVKK

>hPKN2_1873

NLQDFRCCAVLGRGHFGKVLLAEYKNTNEMFAIKALKKGDIVARDEVDSLMCEKRIFETV

NSVRHPFLVNLFACFQTKEHVCFVMEYAAGGDLMMHIHTDVFSEPRAVFYAACVVLGLQY

LHEHKIVYRDLKLDNLLLDTEGFVKIADFGLCKEGMGYGDRTSTFCGTPEFLAPEVLTET

SYTRAVDWWGLGVLIYEMLVGESPFPGDDEEEVFDSIVNDEVRYPRFLSTEAISIMRRLL

RRNPERRLGASEKDAEDVKK

>hPLK1_2140

SRRRYVRGRFLGKGGFAKCFEISDADTKEVFAGKIVPKSLLLKPHQREKMSMEISIHRSL

AHQHVVGFHGFFEDNDFVFVVLELCRRRSLLELHKRRKALTEPEARYYLRQIVLGCQYLH

RNRVIHRDLKLGNLFLNEDLEVKIGDFGLATKVEYDGERKKTLCGTPNYIAPEVLSKKGH

SFEVDVWSIGCIMYTLLVGKPPFETSCLKETYLRIKKNEYSIPKHINPVAASLIQKMLQT

DPTARPTINELLNDEFFT

>hPLK2_2107

TGKRYCRGKVLGKGGFAKCYEMTDLTNNKVYAAKIIPHSRVAKPHQREKIDKEIELHRIL

HHKHVVQFYHYFEDKENIYILLEYCSRRSMAHILKARKVLTEPEVRYYLRQIVSGLKYLH

EQEILHRDLKLGNFFINEAMELKVGDFGLAARLEPLEHRRRTICGTPNYLSPEVLNKQGH

GCESDIWALGCVMYTMLLGRPPFETTNLKETYRCIREARYTMPSSLLAPAKHLIASMLSK

NPEDRPSLDDIIRHDFFL

>hPLK3_2115

SGRTYLKGRLLGKGGFARCYEATDTETGSAYAVKVIPQSRVAKPHQREKILNEIELHRDL

QHRHIVRFSHHFEDADNIYIFLELCSRKSLAHIWKARHTLLEPEVRYYLRQILSGLKYLH

QRGILHRDLKLGNFFITENMELKVGDFGLAARLEPPEQRKKTICGTPNYVAPEVLLRQGH

GPEADVWSLGCVMYTLLCGSPPFETADLKETYRCIKQVHYTLPASLSLPARQLLAAILRA

SPRDRPSIDQILRHDFFT

>hPLK4_1972

KIEDFKVGNLLGKGSFAGVYRAESIHTGLEVAIKMIDKKAMYKAGMVQRVQNEVKIHCQL

KHPSILELYNYFEDSNYVYLVLEMCHNGEMNRYLKNRVKPFSENEARHFMHQIITGMLYL

HSHGILHRDLTLSNLLLTRNMNIKIADFGLATQLKMPHEKHYTLCGTPNYISPEIATRSA

HGLESDVWSLGCMFYTLLIGRPPFDTDTVKNTLNKVVLADYEMPTFLSIEAKDLIHQLLR

RNPADRLSLSSVLDHPFMS

>hPRKG1_1906

KLSDFNIIDTLGVGGFGRVELVQLKSEESKTFAMKILKKRHIVDTRQQEHIRSEKQIMQG

AHSDFIVRLYRTFKDSKYLYMLMEACLGGELWTILRDRGSFEDSTTRFYTACVVEAFAYL

HSKGIIYRDLKPENLILDHRGYAKLVDFGFAKKIGFGKKTWTFCGTPEYVAPEIILNKGH

DISADYWSLGILMYELLTGSPPFSGPDPMKTYNIILRGIDMIEFPKKIAKNAANLIKKLC

RDNPSERLGNLKNGVKDIQK

>hPRKG2_2052

PFQNLEIIATLGVGGFGRVELVKVKNENVAFAMKCIRKKHIVDTKQQEHVYSEKRILEEL

CSPFIVKLYRTFKDNKYVYMLLEACLGGELWSILRDRGSFDEPTSKFCVACVTEAFDYLH

RLGIIYRDLKPENLILDAEGYLKLVDFGFAKKIGSGQKTWTFCGTPEYVAPEVILNKGHD

FSVDFWSLGILVYELLTGNPPFSGVDQMMTYNLILKGIEKMDFPRKITRRPEDLIRRLCR

QNPTERLGNLKNGINDIKK

>hPRKX_2452

SLQDFDTLATVGTGTFGRVHLVKEKTAKHFFALKVMSIPDVIRLKQEQHVHNEKSVLKEV

SHPFLIRLFWTWHDERFLYMLMEYVPGGELFSYLRNRGRFSSTTGLFYSAEIICAIEYLH

SKEIVYRDLKPENILLDRDGHIKLTDFGFAKKLVDRTWTLCGTPEYLAPEVIQSKGHGRA

VDWWALGILIFEMLSGFPPFFDDNPFGIYQKILAGKIDFPRHLDFHVKDLIKKLLVVDRT

RRLGNMKNGANDVKH

>hRAF1_1428

EASEVMLSTRIGSGSFGTVYKGKWHGDVAVKILKVVDPTPEQFQAFRNEVAVLRKTRHVN

ILLFMGYMTKDNLAIVTQWCEGSSLYKHLHVQETKFQMFQLIDIARQTAQGMDYLHAKNI

IHRDMKSNNIFLHEGLTVKIGDFGLATVKSRWSGSQQVEQPTGSVLWMAPEVIRMQDNNP

FSFQSDVYSYGIVLYELMTGELPYSHINNRDQIIFMVGRGYASPDLSKLYKNCPKAMKRL

VADCVKKVKEERPLFPQILSSIELL

>hRET_523

PRKNLVLGKTLGEGEFGKVVKATAFHLKGRAGYTTVAVKMLKENASPSELRDLLSEFNVL

KQVNHPHVIKLYGACSQDGPLLLIVEYAKYGSLRGFLRESRKVGPGYLGSGSRNSSSLDH

PDERALTMGDLISFAWQISQGMQYLAEMKLVHRDLAARNILVAEGRKMKISDFGLSRDVY

EEDSYVKRSQGRIPVKWMAIESLFDHIYTTQSDVWSFGVLLWEIVTLGGNPYPGIPPERL

FNLLKTGHRMERPDNCSEEMYRLMLQCWKQEPDKRPVFADISKDLEKM

>hRIPK2_821

PYHKLADLRYLSRGASGTVSSARHADWRVQVAVKHLHIHTPLLDSERKDVLREAEILHKA

RFSYILPILGICNEPEFLGIVTEYMPNGSLNELLHRKTEYPDVAWPLRFRILHEIALGVN

YLHNMTPPLLHHDLKTQNILLDNEFHVKIADFGLSKWRMMSLSQSRSSKSAPEGGTIIYM

PPENYEPGQKSRASIKHDIYSYAVITWEVLSRKQPFEDVTNPLQIMYSVSQGHRPVINEE

SLPYDIPHRARMISLIESGWAQNPDERPSFLKCLIELEPV

>hROCK1_1441

KAEDYEVVKVIGRGAFGEVQLVRHKSTRKVYAMKLLSKFEMIKRSDSAFFWEERDIMAFA

NSPWVVQLFYAFQDDRYLYMVMEYMPGGDLVNLMSNYDVPEKWARFYTAEVVLALDAIHS

MGFIHRDVKPDNMLLDKSGHLKLADFGTCMKMNKEGMVRCDTAVGTPDYISPEVLKSQGG

DGYYGRECDWWSVGVFLYEMLVGDTPFYADSLVGTYSKIMNHKNSLTFPDDNDISKEAKN

LICAFLTDREVRLGRNGVEEIKRHL

>hROCK2_1454

KAEDYDVVKVIGRGAFGEVQLVRHKASQKVYAMKLLSKFEMIKRSDSAFFWEERDIMAFA

NSPWVVQLFYAFQDDRYLYMVMEYMPGGDLVNLMSNYDVPEKWAKFYTAEVVLALDAIHS

MGLIHRDVKPDNMLLDKHGHLKLADFGTCMKMDETGMVHCDTAVGTPDYISPEVLKSQGG

DGFYGRECDWWSVGVFLYEMLVGDTPFYADSLVGTYSKIMDHKNSLCFPEDAEISKHAKN

LICAFLTDREVRLGRNGVEEIRQHP

>hRPS6KB1_1707

RPECFELLRVLGKGGYGKVFQVRKVTGANTGKIFAMKVLKKAMIVRNAKDTAHTKAERNI

LEEVKHPFIVDLIYAFQTGGKLYLILEYLSGGELFMQLEREGIFMEDTACFYLAEISMAL

GHLHQKGIIYRDLKPENIMLNHQGHVKLTDFGLCKESIHDGTVTHTFCGTIEYMAPEILM

RSGHNRAVDWWSLGALMYDMLTGAPPFTGENRKKTIDKILKCKLNLPPYLTQEARDLLKK

LLKRNAASRLGAGPGDAGEVQA

>hRPS6KB2_1721

GPHCFELLRVLGKGGYGKVFQVRKVQGTNLGKIYAMKVLRKAKIVRNAKDTAHTRAERNI

LESVKHPFIVELAYAFQTGGKLYLILECLSGGELFTHLEREGIFLEDTACFYLAEITLAL

GHLHSQGIIYRDLKPENIMLSSQGHIKLTDFGLCKESIHEGAVTHTFCGTIEYMAPEILV

RSGHNRAVDWWSLGALMYDMLTGSPPFTAENRKKTMDKIIRGKLALPPYLTPDARDLVKK

FLKRNPSQRIGGGPGDAADVQR

>hRSK1_d1_1858

DPSQFELLKVLGQGSYGKVFLVRKVKGSDAGQLYAMKVLKKATLKVRDRVRSKMERDILA

EVNHPFIVKLHYAFQTEGKLYLILDFLRGGDLFTRLSKEVMFTEEDVKFYLAELALALDH

LHSLGIIYRDLKPENILLDEEGHIKITDFGLSKEAIDHDKRAYSFCGTIEYMAPEVVNRR

GHTQSADWWSFGVLMFEMLTGSLPFQGKDRKETMALILKAKLGMPQFLSGEAQSLLRALF

KRNPCNRLGAGIDGVEEIKR

>hRSK1_d2_1595

FTDGYEIKEDIGVGSYSVCKRCVHKATDTEYAVKIIDKSKRDPSEEIEILLRYGQHPNII

TLKDVYDDGKFVYLVMELMRGGELLDRILRQRYFSEREASDVLCTITKTMDYLHSQGVVH

RDLKPSNILYRDESGSPESIRVCDFGFAKQLRAGNGLLMTPCYTANFVAPEVLKRQGYDA

ACDIWSLGILLYTMLAGFTPFANGPDDTPEEILARIGSGKYALSGGNWDSISDAAKDVVS

KMLHVDPHQRLTAMQVLKHPWVV

>hRSK2_d1_1851

DPSQFELLKVLGQGSFGKVFLVKKISGSDARQLYAMKVLKKATLKVRDRVRTKMERDILV

EVNHPFIVKLHYAFQTEGKLYLILDFLRGGDLFTRLSKEVMFTEEDVKFYLAELALALDH

LHSLGIIYRDLKPENILLDEEGHIKLTDFGLSKESIDHEKKAYSFCGTVEYMAPEVVNRR

GHTQSADWWSFGVLMFEMLTGTLPFQGKDRKETMTMILKAKLGMPQFLSPEAQSLLRMLF

KRNPANRLGAGPDGVEEIKR

>hRSK2_d2_1610

FTDGYEVKEDIGVGSYSVCKRCIHKATNMEFAVKIIDKSKRDPTEEIEILLRYGQHPNII

TLKDVYDDGKYVYVVTELMKGGELLDKILRQKFFSEREASAVLFTITKTVEYLHAQGVVH

RDLKPSNILYVDESGNPESIRICDFGFAKQLRAENGLLMTPCYTANFVAPEVLKRQGYDA

ACDIWSLGVLLYTMLTGYTPFANGPDDTPEEILARIGSGKFSLSGGYWNSVSDTAKDLVS

KMLHVDPHQRLTAALVLRHPWIV

>hRSK3_d1_1864

DPSHFELLKVLGQGSFGKVFLVRKVTRPDSGHLYAMKVLKKATLKVRDRVRTKMERDILA

DVNHPFVVKLHYAFQTEGKLYLILDFLRGGDLFTRLSKEVMFTEEDVKFYLAELALGLDH

LHSLGIIYRDLKPENILLDEEGHIKLTDFGLSKEAIDHEKKAYSFCGTVEYMAPEVVNRQ

GHSHSADWWSYGVLMFEMLTGSLPFQGKDRKETMTLILKAKLGMPQFLSTEAQSLLRALF

KRNPANRLGSGPDGAEEIKR

>hRSK3_d2_1617

FSDGYVVKETIGVGSYSECKRCVHKATNMEYAVKVIDKSKRDPSEEIEILLRYGQHPNII

TLKDVYDDGKHVYLVTELMRGGELLDKILRQKFFSEREASFVLHTIGKTVEYLHSQGVVH

RDLKPSNILYVDESGNPECLRICDFGFAKQLRAENGLLMTPCYTANFVAPEVLKRQGYDE

GCDIWSLGILLYTMLAGYTPFANGPSDTPEEILTRIGSGKFTLSGGNWNTVSETAKDLVS

KMLHVDPHQRLTAKQVLQHPWVT

>hRSK4_d1_1869

DPAQFELLKVLGQGSFGKVFLVRKKTGPDAGQLYAMKVLKKASLKVRDRVRTKMERDILV

EVNHPFIVKLHYAFQTEGKLYLILDFLRGGDVFTRLSKEVLFTEEDVKFYLAELALALDH

LHQLGIVYRDLKPENILLDEIGHIKLTDFGLSKESVDQEKKAYSFCGTVEYMAPEVVNRR

GHSQSADWWSYGVLMFEMLTGTLPFQGKDRNETMNMILKAKLGMPQFLSAEAQSLLRMLF

KRNPANRLGSEGVEEIKRHL

>hRSK4_d2_1623

FGEVYELKEDIGVGSYSVCKRCIHATTNMEFAVKIIDKSKRDPSEEIEILMRYGQHPNII

TLKDVFDDGRYVYLVTDLMKGGELLDRILKQKCFSEREASDILYVISKTVDYLHCQGVVH

RDLKPSNILYMDESASADSIRICDFGFAKQLRGENGLLLTPCYTANFVAPEVLMQQGYDA

ACDIWSLGVLFYTMLAGYTPFANGPNDTPEEILLRIGNGKFSLSGGNWDNISDGAKDLLS

HMLHMDPHQRYTAEQILKHSWIT

>hSGK_1964

KPSDFHFLKVIGKGSFGKVLLARHKAEEVFYAVKVLQKKAILKKKEEKHIMSERNVLLKN

VKHPFLVGLHFSFQTADKLYFVLDYINGGELFYHLQRERCFLEPRARFYAAEIASALGYL

HSLNIVYRDLKPENILLDSQGHIVLTDFGLCKENIEHNSTTSTFCGTPEYLAPEVLHKQP

YDRTVDWWCLGAVLYEMLYGLPPFYSRNTAEMYDNILNKPLQLKPNITNSARHLLEGLLQ

KDRTKRLGAKDDFMEIKSH

>hSLK_1481

PEDFWEIIGELGDGAFGKVYKAQNKETSVLAAAKVIDTKSEEELEDYMVEIDILASCDHP

NIVKLLDAFYYENNLWILIEFCAGGAVDAVMLELERPLTESQIQVVCKQTLDALNYLHDN

KIIHRDLKAGNILFTLDGDIKLADFGVSAKNTRTIQRRDSFIGTPYWMAPEVVMCETSKD

RPYDYKADVWSLGITLIEMAEIEPPHHELNPMRVLLKIAKSEPPTLAQPSRWSSNFKDFL

KKCLEKNVDARWTTSQLLQHPFVT

>hSNF1LK_2229

RVGFYDIERTLGKGNFAVVKLARHRVTKTQVAIKIIDKTRLDSSNLEKIYREVQLMKLLN

HPHIIKLYQVMETKDMLYIVTEFAKNGEMFDYLTSNGHLSENEARKKFWQILSAVEYCHD

HHIVHRDLKTENLLLDGNMDIKLADFGFGNFYKSGEPLSTWCGSPPYAAPEVFEGKEYEG

PQLDIWSLGVVLYVLVCGSLPFDGPNLPTLRQRVLEGRFRIPFFMSQDCESLIRRMLVVD

PARRITIAQIRQHRWMR

>hSRC_2204

PRESLRLEVKLGQGCFGEVWMGTWNGTTRVAIKTLKPGTMSPEAFLQEAQVMKKLRHEKL

VQLYAVVSEEPIYIVTEYMSKGSLLDFLKGETGKYLRLPQLVDMAAQIASGMAYVERMNY

VHRDLRAANILVGENLVCKVADFGLARLIEDNEYTARQGAKFPIKWTAPEAALYGRFTIK

SDVWSFGILLTELTTKGRVPYPGMVNREVLDQVERGYRMPCPPECPESLHDLMCQCWRKE

PEERPTFEYLQAFLEDY

>hSTK17A_1337

DGYSLCPGRELGRGKFAVVRKCIKKDSGKEFAAKFMRKRRKGQDCRMEIIHEIAVLELAQ

DNPWVINLHEVYETASEMILVLEYAAGGEIFDQCVADREEAFKEKDVQRLMRQILEGVHF

LHTRDVVHLDLKPQNILLTSESPLGDIKIVDFGLSRILKNSEELREIMGTPEYVAPEILS

YDPISMATDMWSIGVLTYVMLTGISPFLGNDKQETFLNISQMNLSYSEEEFDVLSESAVD

FIRTLLVKKPEDRATAEECLKHPWLT

>hSTK4_2169

PEEVFDVLEKLGEGSYGSVYKAIHKETGQIVAIKQVPVESDLQEIIKEISIMQQCDSPHV

VKYYGSYFKNTDLWIVMEYCGAGSVSDIIRLRNKTLTEDEIATILQSTLKGLEYLHFMRK

IHRDIKAGNILLNTEGHAKLADFGVAGQLTDTMAKRNTVIGTPFWMAPEVIQEIGYNCVA

DIWSLGITAIEMAEGKPPYADIHPMRAIFMIPTNPPPTFRKPELWSDNFTDFVKQCLVKS

PEQRATATQLLQHPFVR

>hSYK_1667

RKLLTLEDKELGSGNFGTVKKGYYQMKKVVKTVAVKILKNEANDPALKDELLAEANVMQQ

LDNPYIVRMIGICEAESWMLVMEMAELGPLNKYLQQNRHVKDKNIIELVHQVSMGMKYLE

ESNFVHRDLAARNVLLVTQHYAKISDFGLSKALRADENYYKAQTHGKWPVKWYAPECINY

YKFSSKSDVWSFGVLMWEAFSYGQKPYRGMKGSEVTAMLEKGERMGCPAGCPREMYDLMN

LCWTYDVENRPGFAAVELRLRNY

>hTAOK3_2006

PEELFIGLHEIGHGSFGAVYFATNAHTSEVVAIKKMSYSGKQTHEKWQDILKEVKFLRQL

KHPNTIEYKGCYLKEHTAWLVMEYCLGSASDLLEVHKKPLQEVEIAAITHGALHGLAYLH

SHALIHRDIKAGNILLTEPGQVKLADFGSASMASPANSFVGTPYWMAPEVILAMDEGQYD

GKVDIWSLGITCIELAERKPPLFNMNAMSALYHIAQNDSPTLQSNEWTDSFRRFVDYCLQ

KIPQERPTSAELLRHDFVR

>hTGFbR1_361

IARTIVLQESIGKGRFGEVWRGKWRGEEVAVKIFSSREERSWFREAEIYQTVMLRHENIL

GFIAADNKDNGTWTQLWLVSDYHEHGSLFDYLNRYTVTVEGMIKLALSTASGLAHLHMEI

VGTQGKPAIAHRDLKSKNILVKKNGTCCIADLGLAVRHDSATDTIDIAPNHRVGTKRYMA

PEVLDDSINMKHFESFKRADIYAMGLVFWEIARRCSIGGIHEDYQLPYYDLVPSDPSVEE

MRKVVCEQKLRPNIPNRWQSCEALRVMAKIMRECWYANGAARLTALRIKKTLSQL

>hTGFbR2_256

ELLPIELDTLVGKGRFAEVYKAKLKQNTSEQFETVAVKIFPYEEYASWKTEKDIFSDINL

KHENILQFLTAEERKTELGKQYWLITAFHAKGNLQEYLTRHVISWEDLRKLGSSLARGIA

HLHSDHTPCGRPKMPIVHRDLKSSNILVKNDLTCCLCDFGLSLRLDPTLSVDDLANSGQV

GTARYMAPEVLESRMNLENAESFKQTDVYSMALVLWEMTSRCNAVGEVKDYEPPFGSKVR

EHPCVESMKDNVLRDRGRPEIPSFWLNHQGIQMVCETLTECWDHDPEARLTAQCVAERFS

EL

>hTIE2_876

DWNDIKFQDVIGEGNFGQVLKARIKKDGLRMDAAIKRMKEYASKDDHRDFAGELEVLCKL

GHHPNIINLLGACEHRGYLYLAIEYAPHGNLLDFLRKSRVLETDPAFAIANSTASTLSSQ

QLLHFAADVARGMDYLSQKQFIHRDLAARNILVGENYVAKIADFGLSRGQEVYVKKTMGR

LPVRWMAIESLNYSVYTTNSDVWSYGVLLWEIVSLGGTPYCGMTCAELYEKLPQGYRLEK

PLNCDDEVYDLMRQCWREKPYERPSFAQILVSLNRM

>hTNK2_1259

GEKDLRLLEKLGDGSFGVVRRGEWDAPSGKTVSVAVKCLKPDVLSQPEAMDDFIREVNAM

HSLDHRNLIRLYGVVLTPPMKMVTELAPLGSLLDRLRKHQGHFLLGTLSRYAVQVAEGMG

YLESKRFIHRDLAARNLLLATRDLVKIGDFGLMRALPQNDDHYVMQEHRKVPFAWCAPES

LKTRTFSHASDTWMFGVTLWEMFTYGQEPWIGLNGSQILHKIDKEGERLPRPEDCPQDIY

NVMVQCWAHKPEDRPTFVALRDFLLEA

>hTXK_2259

DPSELAFIKEIGSGQFGVVHLGEWRSHIQVAIKAINEGSMSEEDFIEEAKVMMKLSHSKL

VQLYGVCIQRKPLYIVTEFMENGCLLNYLRENKGKLRKEMLLSVCQDICEGMEYLERNGY

IHRDLAARNCLVSSTCIVKISDFGMTRYVLDDEYVSSFGAKFPIKWSPPEVFLFNKYSSK

SDVWSFGVLMWEVFTEGKMPFENKSNLQVVEAISEGFRLYRPHLAPMSIYEVMYSCWHEK

PEGRPTFAELLRAVTEI

>hTYK2_d1_687

DQKEITQLSHLGQGTRTNVYEGRLRVEGSGDPEEGKMDDEDPLVPGRDRGQELRVVLKVL

DPSHHDIALAFYETASLMSQVSHTHLAFVHGVCVRGPENIMVTEYVEHGPLDVWLRRERG

HVPMAWKMVVAQQLASALSYLENKNLVHGNVCGRNILLARLGLAEGTSPFIKLSDPGVGL

GALSREERVERIPWLAPECLPGGANSLSTAMDKWGFGATLLEICFDGEAPLQSRSPSEKE

HFYQRQHRLPEPSCPQLATLTSQCLTYEPTQRPSFRTILRDLTRL

>hTYK2_d2_819

HKRYLKKIRDLGEGHFGKVSLYCYDPTNDGTGEMVAVKALKADCGPQHRSGWKQEIDILR

TLYHEHIIKYKGCCEDQGEKSLQLVMEYVPLGSLRDYLPRHSIGLAQLLLFAQQICEGMA

YLHAQHYIHRDLAARNVLLDNDRLVKIGDFGLAKAVPEGHEYYRVREDGDSPVFWYAPEC

LKEYKFYYASDVWSFGVTLYELLTHCDSSQSPPTKFLELIGIAQGQMTVLRLTELLERGE

RLPRPDKCPCEVYHLMKNCWETEASFRPTFENLIPILKTV

>hTYRO3_897

PEQQFTLGRMLGKGEFGSVREAQLKQEDGSFVKVAVKMLKADIIASSDIEEFLREAACMK

EFDHPHVAKLVGVSLRSRAKGRLPIPMVILPFMKHGDLHAFLLASRIGENPFNLPLQTLI

RFMVDIACGMEYLSSRNFIHRDLAARNCMLAEDMTVCVADFGLSRKIYSGDYYRQGCASK

LPVKWLALESLADNLYTVQSDVWAFGVTMWEIMTRGQTPYAGIENAEIYNYLIGGNRLKQ

PPECMEDVYDLMYQCWSADPKQRPSFTCLRMELENI

>hVEGFR1_61

ARERLKLGKSLGRGAFGKVVQASAFGIKKSPTCRTVAVKMLKEGATASEYKALMTELKIL

THIGHHLNVVNLLGACTKQGGPLMVIVEYCKYGNLSNYLKSKRDLFFLNKDAQEDKSLSD

VEEEEDSDGFYKEPITMEDLISYSFQVARGMEFLSSRKCIHRDLAARNILLSENNVVKIC

DFGLARDIYKNPDYVRKGDTRLPLKWMAPESIFDKIYSTKSDVWSYGVLLWEIFSLGGSP

YPGVQMDEDFCSRLREGMRMRAPEYSTPEIYQIMLDCWHRDPKERPRFAELVEKLGDL

>hVEGFR2_66

PRDRLKLGKPLGRGAFGQVIEADAFGIDKTATCRTVAVKMLKEGATHSEHRALMSELKIL

IHIGHHLNVVNLLGACTKPGGPLMVIVEFCKFGNLSTYLRSKRNEFVPYKTKVEEKSLSD

VEEEEAPEDLYKDFLTLEHLICYSFQVAKGMEFLASRKCIHRDLAARNILLSEKNVVKIC

DFGLARDIYKDPDYVRKGDARLPLKWMAPETIFDRVYTIQSDVWSFGVLLWEIFSLGASP

YPGVKIDEEFCRRLKEGTRMRAPDYTTPEMYQTMLDCWHGEPSQRPTFSELVEHLGNL

>hVEGFR3_87

PRERLHLGRVLGYGAFGKVVEASAFGIHKGSSCDTVAVKMLKEGATASEHRALMSELKIL

IHIGNHLNVVNLLGACTKPQGPLMVIVEFCKYGNLSNFLRAKRDAFSPCAEKKTEGGARR

ASPDQEAEDLWLSPLTMEDLVCYSFQVARGMEFLASRKCIHRDLAARNILLSESDVVKIC

DFGLARDIYKDPDYVRKGSARLPLKWMAPESIFDKVYTTQSDVWSFGVLLWEIFSLGASP

YPGVQINEEFCQRLRDGTRMRAPELATPAIRRIMLNCWSGDPKARPAFSELVEILGDL

>hWEE1_913

YTTEFHELEKIGSGEFGSVFKCVKRLDGCIYAIKRSKKPLAGSVDEQNALREVYAHAVLG

QHSHVVRYFSAWAEDDHMLIQNEYCNGGSLADAISENYRIMSYFKEAELKDLLLQVGRGL

RYIHSMSLVHMDIKPSNIFISRTSIPNAASEEGDEDDWASNKVMFKIGDLGHVTRISSPQ

VEEGDSRFLANEVLQENYTHLPKADIFALALTVVCAAGAEPLPRNGDQWHEIRQGRLPRI

PQVLSQEFTELLKVMIHPDPERRPSAMALVKHSVLL

>hYES1_2223

PRESLRLEVKLGQGCFGEVWMGTWNGTTKVAIKTLKPGTMMPEAFLQEAQIMKKLRHDKL

VPLYAVVSEEPIYIVTEFMSKGSLLDFLKEGDGKYLKLPQLVDMAAQIADGMAYIERMNY

IHRDLRAANILVGENLVCKIADFGLARLIEDNEYTARQGAKFPIKWTAPEAALYGRFTIK

SDVWSFGILQTELVTKGRVPYPGMVNREVLEQVERGYRMPCPQGCPESLHELMNLCWKKD

PDERPTFEYIQSFLEDY

>hZAK_2482

KFDDLQFFENCGGGSFGSVYRAKWISQDKEVAVKKLLKIEKEAEILSVLSHRNIIQFYGV

ILEPPNYGIVTEYASLGSLYDYINSNRSEEMDMDHIMTWATDVAKGMHYLHMEAPVKVIH

RDLKSRNVVIAADGVLKICDFGASRFHNHTTHMSLVGTFPWMAPEVIQSLPVSETCDTYS

YGVVLWEMLTREVPFKGLEGLQVAWLVVEKNERLTIPSSCPRSFAELLHQCWEADAKKRP

SFKQIISILESM

>hZAP70_1646

RDNLLIADIELGCGNFGSVRQGVYRMRKKQIDVAIKVLKQGTEKADTEEMMREAQIMHQL

DNPYIVRLIGVCQAEALMLVMEMAGGGPLHKFLVGKREEIPVSNVAELLHQVSMGMKYLE

EKNFVHRDLAARNVLLVNRHYAKISDFGLSKALGADDSYYTARSAGKWPLKWYAPECINF

RKFSSRSDVWSYGVTMWEALSYGQKPYKKMKGPEVMAFIEQGKRMECPPECPPELYALMS

DCWIYKWEDRPDFLTVEQRMRAC

>hp38a_494

VPERYQNLSPVGSGAYGSVCAAFDTKTGLRVAVKKLSRPFQSIIHAKRTYRELRLLKHMK

HENVIGLLDVFTPARSLEEFNDVYLVTHLMGADLNNIVKCQKLTDDHVQFLIYQILRGLK

YIHSADIIHRDLKPSNLAVNEDCELKILDFGLARHTDDEMTGYVATRWYRAPEIMLNWMH

YNQTVDIWSVGCIMAELLTGRTLFPGTDHIDQLKLILRLVGTPGAELLKKISSESARNYI

QSLTQMPKMNFANVFIGANPLAVDLLEKMLVLDSDKRITAAQALAHAYFA

>hp38b_321

VPQRLQGLRPVGSGAYGSVCSAYDARLRQKVAVKKLSRPFQSLIHARRTYRELRLLKHLK

HENVIGLLDVFTPATSIEDFSEVYLVTTLMGADLNNIVKCQAGAHQGARLALDEHVQFLV

YQLLRGLKYIHSAGIIHRDLKPSNVAVNEDCELRILDFGLARQADEEMTGYVATRWYRAP

EIMLNWMHYNQTVDIWSVGCIMAELLQGKALFPGSDYIDQLKRIMEVVGTPSPEVLAKIS

SEHARTYIQSLPPMPQKDLSSIFRGANPLAIDLLGRMLVLDSDQRVSAAEALAHAYFS

>hp38d_568

LPKTYVSPTHVGSGAYGSVCSAIDKRSGEKVAIKKLSRPFQSEIFAKRAYRELLLLKHMQ

HENVIGLLDVFTPASSLRNFYDFYLVMPFMQTDLQKIMGMEFSEEKIQYLVYQMLKGLKY

IHSAGVVHRDLKPGNLAVNEDCELKILDFGLARHADAEMTGYVVTRWYRAPEVILSWMHY

NQTVDIWSVGCIMAEMLTGKTLFKGKDYLDQLTQILKVTGVPGTEFVQKLNDKAAKSYIQ

SLPQTPRKDFTQLFPRASPQAADLLEKMLELDVDKRLTAAQALTHPFFE

>hp38g_474

VRAVYRDLQPVGSGAYGAVCSAVDGRTGAKVAIKKLYRPFQSELFAKRAYRELRLLKHMR

HENVIGLLDVFTPDETLDDFTDFYLVMPFMGTDLGKLMKHEKLGEDRIQFLVYQMLKGLR

YIHAAGIIHRDLKPGNLAVNEDCELKILDFGLARQADSEMTGYVVTRWYRAPEVILNWMR

YTQTVDIWSVGCIMAEMITGKTLFKGSDHLDQLKEIMKVTGTPPAEFVQRLQSDEAKNYM

KGLPELEKKDFASILTNASPLAVNLLEKMLVLDAEQRVTAGEALAHPYFE
