# Supplementary material for: Prediction of Chemical-Protein Interactions Network with Weighted Network-Based Inference Method
Source: PLoS One. 2012 Jul 16;7(7):e41064. doi: 10.1371/journal.pone.0041064 (PMC3397956; doi:10.1371/journal.pone.0041064)
Supplement: Table S4 — The performance of difference inference methods on the new external validation set. The new external validation set was constructed after removing 50% high similar compounds with top Tanimoto similarity using MACCS keys on the original external validation set (Table S3) of GPCRs and kinases. (PDF) [file pone.0041064.s006.pdf]

Table S4. The performance of difference inference methods in the new external validation set after removing 50% high similar compounds with top tanimoto similarity using MACCS keys on the original external validation set (Table S3) for GPCRs and kinases.

Targets	Methods	C_i (P_a, P_b, …, P_m)			P_j (C_a, C_b, …, C_n)		
		R	ER	AUC	R	ER	AUC
GPCRs	NBI	0.402	1.95	0.664	0.668	1.54	0.574
	NWNBI	0.453	2.20	0.653	0.668	1.54	0.574
	EWNBI	0.441	2.14	0.657	0.646	1.48	0.576
	DBSI-T	0.336	1.63	0.686	0.685	1.58	0.621
	DBSI-C	0.344	1.67	0.680	0.706	1.62	0.620
	DBSI-F	0.346	1.68	0.679	0.716	1.65	0.625
	DBSI-R	0.347	1.68	0.685	0.714	1.64	0.618
	TBSI	0.448	2.17	0.675	0.566	1.30	0.543
Kinome	NBI	0.462	4.76	0.818	0.374	1.76	0.587
	NWNBI	0.494	5.08	0.833	0.374	1.76	0.587
	EWNBI	0.504	5.19	0.826	0.301	1.41	0.582
	DBSI-T	0.564	5.81	0.855	0.233	1.09	0.550
	DBSI-C	0.543	5.59	0.855	0.225	1.06	0.537
	DBSI-F	0.555	5.72	0.855	0.222	1.05	0.537
	DBSI-R	0.543	5.59	0.855	0.226	1.06	0.536
	TBSI	0.068	0.700	0.347	0.163	0.77	0.499

All performances were evaluated based on top 20 predicted lists. NBI, network-based inference; NWNBI, node weighted network-based inference; EWNBI, edge weighted network-based inference; DBSI-T, drug-based similarity inference with Tanimoto similarity score; C, Cosine similarity score; F, Forbes similarity score; R, Russell-rao similarity score; TBSI, target-based similarity inference; R, recall; ER, recall enhancement; AUC, the area under the receiver operating characteristic curve; C_i (P_a, P_b, …, P_m) represents the prioritization of new targets for a given chemical; P_j (C_a, C_b, …, C_n) represents the prioritization of new chemicals for a given protein.
